# A mechanistic integrative computational model of macrophage polarization: Implications in human pathophysiology

**DOI:** 10.1371/journal.pcbi.1007468

**Published:** 2019-11-18

**Authors:** Chen Zhao, Adam C. Mirando, Richard J. Sové, Thalyta X. Medeiros, Brian H. Annex, Aleksander S. Popel

**Affiliations:** 1 Department of Biomedical Engineering, Johns Hopkins University School of Medicine, Baltimore, Maryland, United States of America; 2 Robert M. Berne Cardiovascular Research Center, University of Virginia, Charlottesville, Virginia, United States of America; 3 Divison of Cardiovascular Medicine, Department of Medicine, University of Virginia, Charlottesville, Virginia, United States of America; Purdue University, UNITED STATES

## Abstract

Macrophages respond to signals in the microenvironment by changing their functional phenotypes, a process known as polarization. Depending on the context, they acquire different patterns of transcriptional activation, cytokine expression and cellular metabolism which collectively constitute a continuous spectrum of phenotypes, of which the two extremes are denoted as classical (M1) and alternative (M2) activation. To quantitatively decode the underlying principles governing macrophage phenotypic polarization and thereby harness its therapeutic potential in human diseases, a systems-level approach is needed given the multitude of signaling pathways and intracellular regulation involved. Here we develop the first mechanism-based, multi-pathway computational model that describes the integrated signal transduction and macrophage programming under M1 (IFN-γ), M2 (IL-4) and cell stress (hypoxia) stimulation. Our model was calibrated extensively against experimental data, and we mechanistically elucidated several signature feedbacks behind the M1-M2 antagonism and investigated the dynamical shaping of macrophage phenotypes within the M1-M2 spectrum. Model sensitivity analysis also revealed key molecular nodes and interactions as targets with potential therapeutic values for the pathophysiology of peripheral arterial disease and cancer. Through simulations that dynamically capture the signal integration and phenotypic marker expression in the differential macrophage polarization responses, our model provides an important computational basis toward a more quantitative and network-centric understanding of the complex physiology and versatile functions of macrophages in human diseases.

## Author summary

As essential regulators of the immune system, macrophages can be polarized to acquire distinct phenotypes in response to a wide range of signals in the tissue microenvironment, such as bacterial products, endogenous cytokines, cell damage and stress. Decades of research has shown that a number of signaling pathways can regulate this process and determine the functional phenotypes of macrophages in physiology as well as various disease scenarios, and recent studies suggest that macrophage polarization is indeed a dynamic process and that the canonical dichotomous notion with only classical (M1) and alternative (M2) activation states is oversimplifying the continuous spectrum of polarized macrophage phenotypes observed in health and disease. To investigate the mechanistic and therapeutic aspects associated with differentially polarized macrophages, we formulated and calibrated a multi-pathway computational model based on literature knowledge and quantitative experimental datasets to systematically describe the integrative regulation of macrophage transcriptional programs and phenotype markers under different stimuli combinations. Our systems-level model is a key building block of a potential “virtual macrophage” simulation platform that can enable researchers to efficiently generate mechanistic hypotheses and assess macrophage-based therapeutic strategies for human diseases.

## Introduction

Macrophages are a class of innate immune cells that play essential roles in the progression and resolution of inflammatory responses, which are key to a variety of major human diseases [1]. As monocyte-derived macrophages that are recruited to the site of disease from the circulation or as local tissue-resident macrophages, these phagocytic cells perform versatile biological functions in addition to clearing out dying cells and tissues. They interact with other cellular components within the tissue (e.g. T cells, fibroblasts, endothelial cells, cancer cells), through the expression and secretion of various cytokines and signals, to modulate crucial cell-level responses (e.g. proliferation, T-helper type 1/2 polarization, antigen presentation) that collectively regulate tissue-level events such as inflammation, tissue remodeling, angiogenesis, arteriogenesis, tumor growth and metastasis [1, 2]. A wealth of studies has investigated the differential phenotypes and corresponding regulatory functions of macrophages in disease settings including in major human diseases such as cancer, infectious and inflammatory disease, cardiovascular disease, and metabolic disease; evidence from *in vitro* and *in vivo* experiments confirmed the highly plastic nature of monocytes-macrophages, which suggest that cells of this lineage can be flexibly programmed by disease-driven environmental cues to exhibit a wide spectrum of activation and functional states [1–5]. Pursuing this idea, in the last decade there have been tremendous efforts from both academia and pharmaceutical industry to develop therapeutics that aim to treat human diseases by modulating and reversing the polarized macrophage phenotypes induced by the disease pathology, most notably in a multitude of cancer indications; besides, rich preclinical and clinical evidence also suggested that cardiovascular disease may be another promising field that can benefit from similar strategies [6–10].

The concept of differential macrophage polarization and phenotypes can be described in terms of the activation of different signaling pathways and transcription factors, together with the expression and secretion of a set of markers and cytokines. The canonically activated macrophages (CAM, or M1) and the alternatively activated macrophages (AAM or M2) represent the extremes of the total macrophage polarization spectrum, while in physiology and pathology most macrophages display “M1-like” or “M2-like” phenotypes. M1 (or M1-like) phenotypes are often induced by pro-inflammatory cytokines such as IFN-γ (interferon gamma), TNF-α (tumor necrosis factor alpha) and IL-1β (interleukin 1 beta) as well as certain pathogen- and damage-associated molecular patterns (PAMP, DAMP) such as LPS (lipopolysaccharides) from gram-negative bacteria and HMGB1 (high mobility group box 1) which is a nuclear protein highly secreted by damaged or necrotic cells [11, 12]. M1-like macrophages are typically characterized by their antibacterial and antitumor functions, along with the high production of various pro-inflammatory cytokines as well as reactive nitrogen and oxygen species (RNS, ROS). On the other hand, cytokines such as IL-4/IL-13, IL-10 and TGF-β (transforming growth factor beta) will contribute to M2 (or M2-like), anti-inflammatory phenotypes which are broadly involved in immunosuppression, angiogenesis, and tissue repair [1, 13].

Quite a large number of signal transduction details in the major M1-M2 ligand-induced pathways have been characterized by the numerous biochemical and biophysical studies over the past two decades. A major family of transcription factors that controls the M1-M2 polarization is the STAT proteins (signal transducer and activator of transcription), which are generally activated by ligand-receptor induced mechanisms through the associated JAKs (Janus kinases) in response to a number of M1 and M2 inducers such as type I and II interferons and several interleukins [14]. IFN-γ is known to drive STAT1 activation and downstream M1 marker expression such as iNOS (inducible nitric oxide synthase) [15], IL-12 [16] and TNFα [17], while IL-4/IL-13 will primarily induce STAT6 activation and M2 markers such as Arg-1 (arginase 1) [18], MRC1 (CD206) and IL-10 [19, 20]. Other STATs such as STAT2 (activated by IFN-α/β), STAT3 (by IL-10), and STAT4 (by IL-12 plus IL-18) also participate in the polarization of macrophages [21, 22], and it was shown that certain STATs can directly (e.g. via binding and sequestration) or indirectly (e.g. via induction of SOCS proteins–suppressor of cytokine signaling) influence the activation of other STATs, thereby forming positive and negative feedback loops during the programming of M1-M2 phenotypes [23, 24]. Apart from the STATs, various other transcription factors and signaling hubs such as NF-κB, MAPKs (mitogen-activated protein kinases), AKT, and HIFs (hypoxia inducible factors) as well as post-transcriptional regulators including a number of microRNAs (miRs), can also direct the M1-M2 polarization process in response to pathological stimuli [25]. Plus the findings that sequential autocrine induction and signaling of certain M1 and M2 cytokines such as interferons and IL-10 were critical for the phenotypic functions of polarized macrophages [26–28], it again points to the continuum hypothesis that simultaneous and sequential activation of multiple cellular pathways, instead of a single stimulus activating a single pathway, is more likely the underlying biological mechanisms of the dynamic macrophage phenotypes observed experimentally [1, 29, 30]. Therefore, a systems-level approach which allows investigation of both the complex multi-modal signal transduction and cross-talks as well as the temporal expression of phenotypic cytokines and markers is key to the integrative understanding of macrophage polarization and functions in health and disease.

To address this complexity at the cell level, previous modeling studies have primarily utilized Boolean networks to evaluate the gene expression outcomes modulated by multiple ligand-induced signaling pathways during macrophage polarization in a general context [29, 31–33]. Due to the discrete nature of Boolean models, a detailed, quantitative description of the time-course activation of key intermediate signaling hubs and transcription factors within the M1-M2 spectrum, especially in pathological contexts, is still lacking. In addition, the influence of hypoxia and metabolic changes, which are crucial drivers and signatures in major diseases such as peripheral arterial disease (PAD), myocardial infarction, and cancer [34], on the polarization of macrophage functions has not been systematically characterized as a core component within the M1-M2 signaling network. Therefore, in this paper, we computationally formulate and analyze a novel mass-action based mechanistic model that can dynamically and quantitatively describe the complex pathway regulation and phenotypic marker expression initiated by M1 (IFN-γ), M2 (IL-4) and cell stress (hypoxia) inducers. This data-driven model not only can reproduce experimental time-course observations relating to different macrophage phenotype perturbations (e.g. 70+ conditions), but also suggested novel insights regarding the hierarchical and temporal control of M1-M2 features through an integrative analysis of direct cytokine signaling, hypoxic response, transcriptional and post-transcriptional regulation, and autocrine feedbacks. In addition, we simulated the model in contexts that mimicked pathological signatures in tissue ischemia and tumor and tested different strategies to modulate therapeutically favorable macrophage polarization against these conditions. The mechanistic development of our *in silico* model itself, together with the findings presented in this study, serves as an important basis towards a more advanced, quantitative systems-level understanding of macrophage polarization and macrophage-based therapeutic interventions in human disease settings.

## Results

### Overview of the computational model and its mechanistic formulation

**Fig 1:**
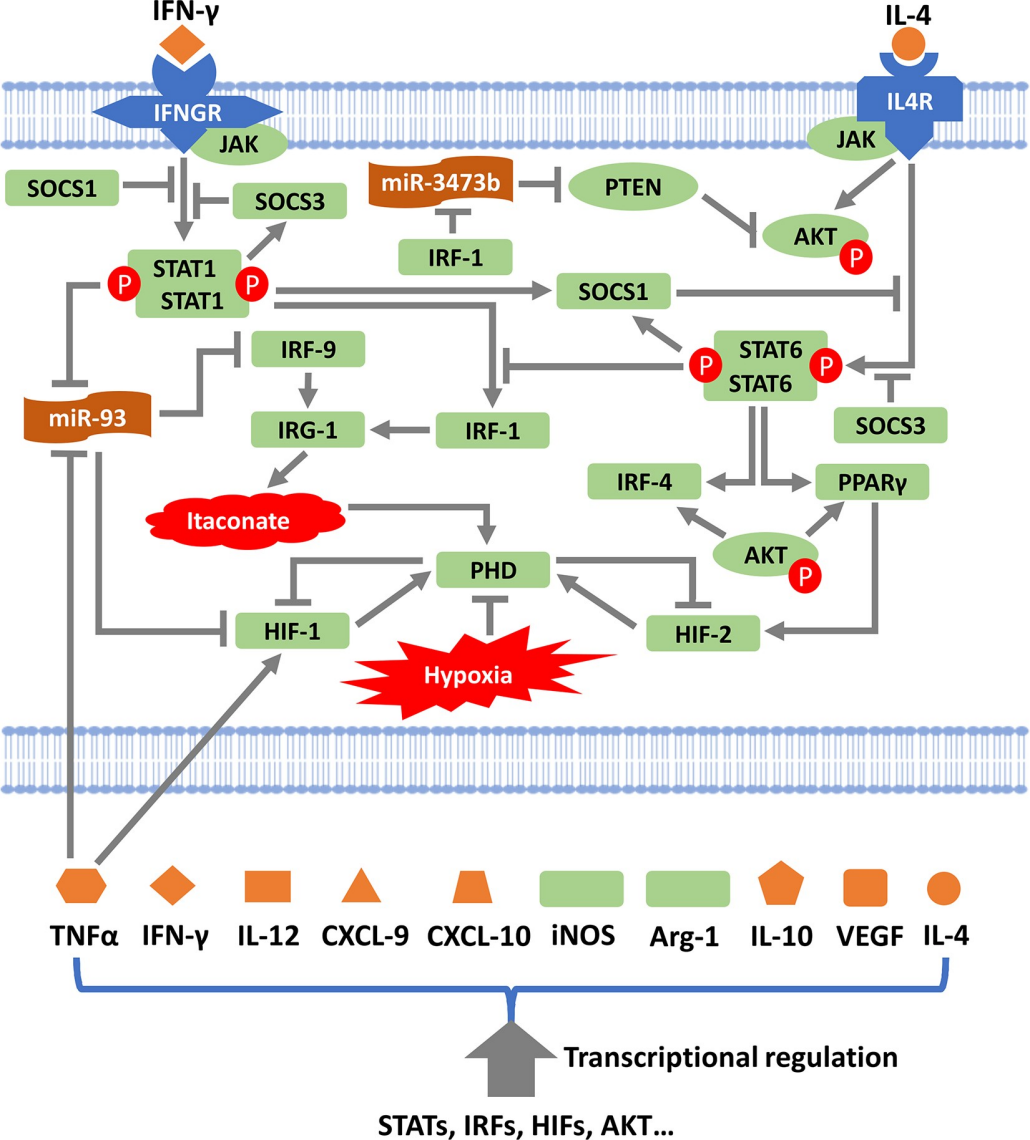
Multiple signaling axes regulate macrophage phenotype and polarization. Macrophage polarization is dynamically controlled by different receptor-mediated signaling pathways, cell stress, transcriptional and post-transcriptional regulators (e.g. miRs), which collectively lead to differential expression of a panel of macrophage phenotype markers (including both intracellular and secreted proteins). Arrow indicates activation,–| symbol indicates inhibition. Green shapes indicate intracellular proteins, orange shapes indicate secreted products. This figure is an overview of model formulation; full mechanistic details of the computational model are presented in S1 Fig and S1 and S2 Tables. It should also be noted that this figure only describes a subset of the M1- and M2-related pathways and markers.

The basic framework of our computational model (Figs 1 and S1) provides a physiology-based and literature data-driven description of macrophage polarization, which can be divided into three subparts: (i) IFN-γ-driven pathway, (ii) IL-4-driven pathway, and (iii) hypoxia-driven pathway. IFN-γ is known as a potent activator of the T-helper type 1 (Th1) immune response and it also strongly induces the inflammatory phenotypes in macrophages, as characterized by the high production and secretion of an array of pro-inflammatory cytokines and chemokines [35]. The model describes the mechanistic activation of two major signaling mediators downstream of IFN-γ mediated receptor activation, STAT1 and IRF-1 (interferon regulatory factor 1), which further leads to direct transcriptional activation and subsequent protein production of canonical M1 markers including iNOS, TNFα, IL-12, CXCL9 (C-X-C motif chemokine ligand 9) and CXCL10 [11]. The Th2 cytokine IL-4, on the other hand, skews macrophages toward anti-inflammatory and pro-angiogenic phenotypes primarily through the activation of STAT6 and IRF-4: STAT6 was shown to influence IRF-4 abundance in macrophages upon IL-4 stimulation, while both factors can contribute to the production of key M2 markers (e.g. Arg-1) [18, 36]. STAT6 also upregulates the cellular expression of PPARγ (peroxisome proliferator-activated receptor γ) which is a signature of oxidative metabolism and transcriptional regulation associated with M2-like macrophages [37, 38], and STAT6 can counteract the IFN-γ-induced upregulation of IRF-1 by directly suppressing STAT1 transcriptional activities [39]. In addition, IL-4 mediated receptor signaling in macrophages is capable of switching on the phosphoinositide 3-kinase (PI3K)/AKT pathway, which in turn would promote IRF-4 and PPARγ activation as well as IL-10 and VEGF (vascular endothelial growth factor) synthesis to reduce inflammation and enhance angiogenesis [36, 40, 41]. On the contrary, IFN-γ signaling would reduce AKT activation presumably by inducing the negative regulator PTEN (phosphatase and tensin homolog) through post-transcriptional regulation (e.g. via miR-3473b) [42]. IFN-γ and IL-4 can signal through the associated STATs (STAT1 for IFN-γ, STAT6 for IL-4) to transcriptionally induce the expression of SOCS proteins, which in turn will negatively feedback to deactivate both STATs [24]. Details of the signal transduction and regulation discussed above are described mechanistically in the model subparts (i) and (ii).

Hypoxia, an essential pathological feature in numerous human diseases, can potently drive macrophage phenotypic polarizations, and in our model the influence of hypoxia is assumed to be represented through its two major effectors, HIF-1 and HIF-2 (Fig 1) [43]. The oxygen sensing module is an adapted version of a previous model developed by our group [44]; in the current model, the synthesis of HIF-2α is further linked to the IL-4 axis and is controlled by PPARγ [45], while the synthesis of HIF-1α is positively modulated by TNFα [46]. The HIF-1/2 alpha subunits, when stabilized in hypoxia and associated with HIF-1β in the nucleus, can upregulate the production of both M1 and M2 markers including IFN-γ, iNOS, Arg-1 and the pro-angiogenic factor VEGF [47–49]. As described by the model, miR-93 can directly inhibit HIF-1α at the mRNA level [50, 51], while its cellular abundance is negatively regulated by pro-inflammatory stimuli, namely IFN-γ and TNFα [52]. IRF-9 mRNA is also directly targeted by miR-93 [53], and IRF-9 together with IRF-1 will upregulate IRG-1 (immunoresponsive gene 1/aconitate decarboxylase 1) which then leads to increased production and accumulation of itaconate (or itaconic acid), an endogenous metabolite in macrophages with immune-modulatory functions [54, 55]. High levels of itaconate was shown to limit ROS production, and in turn this is linked to the decreased production of HIFs as our model assumed that ROS would block PHD (prolyl hydroxylase) activity [56, 57].

As discussed above, the overall model is comprised of 34 “unique” species (including functionally unique mRNA, miR and protein products, plus non-gene compounds such as oxygen and itaconate) divided into three interconnected subparts/pathways. To present the model results, we will first focus on the immediate model behaviors specifically under the influence of the three pathways, followed by a systematic analysis of the macrophage signaling network in disease-related pathological contexts (in both cases, all model components and reactions are active simultaneously during simulations, instead of single subparts being isolated and tested for each scenario). More mechanistic details about the model formulation can be found in the Materials and Methods section and in S1 and S2 Tables and S1 Fig (including a comprehensive list and diagram with all model nodes and biochemical reactions).

### Calibration and analysis of model subparts

To reflect the physiology of unpolarized macrophages, the initial concentrations of most modeled species (e.g. proteins, mRNAs, miRs), which equal to the model-generated equilibrium levels without any external treatment, are calibrated (through parameter optimization) against absolute copy number measurements from literature. From there, the model generates simulations in response to various stimuli and is calibrated extensively against literature experimental data, including a total of more than 70 sets of experimental measurements (both time-course and single timepoint) with over 300 datapoints. Details about model calibration are described in the following modules (and also in Materials and Methods and S2–S4 Figs).

#### IFN-γ-driven pathway

**Fig 2:**
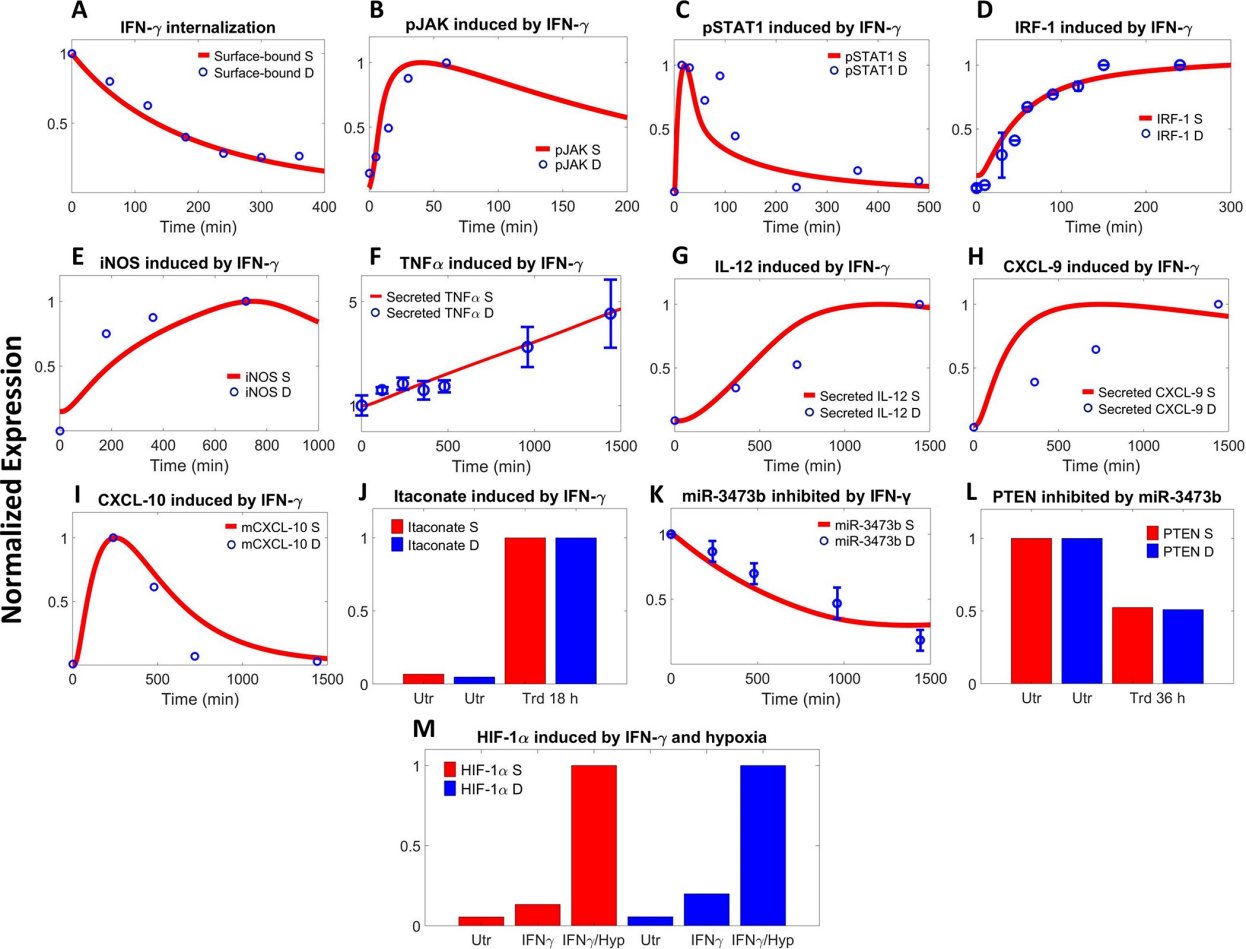
IFN-γ-mediated signaling controls macrophage phenotype. In response to different doses of IFN-γ treatment, the model simulations are compared with corresponding literature time-course data including (A) degradation of receptor-bound IFN-γ [59], (B) phosphorylation of receptor-associated JAK [15], (C) phosphorylation of STAT1 [60], (D) expression of IRF-1 [61], (E) expression of iNOS [62], (F) levels of secreted TNFα [17], (G) levels of secreted IL-12 [16], (H) levels of secreted CXCL-9 [16], (I) intracellular mRNA expression of CXCL-10 [63], (K) expression of miR-3473b [42], plus single timepoint measurements including intracellular levels of (J) itaconic acid at 18 h [64], (L) PTEN at 36 h (in response to miR-3473b mimic transfection, see also S2H Fig) [42], and (M) HIF-1α (in response to IFN-γ with or without hypoxia) [48]. (A-M) All literature data are measured in macrophage cell lines and values are for protein levels unless noted otherwise. Y-axes show normalized expression respectively (A-E, G-I, K: simulations and data are normalized to the maximum expression; F, L: normalized to the no-treatment/time 0 expression; J: normalized to the expression at 18 h; M: normalized to the expression under IFN-γ treatment with hypoxia). S–simulation, D–literature data, Utr–untreated, Trd–IFN-γ treated, Hyp–hypoxia.

The simulated dynamics of various nodes within the IFN-γ pathway agree well with quantitative literature experimental data (Figs 2 and S2). In macrophages, binding of IFN-γ with its receptors on the cell surface (Fig 2A) will lead to rapid activation (e.g. by phosphorylation) of receptor-associated JAK proteins (Fig 2B), and this in turn will activate STAT1 through phosphorylation, which tends to peak transiently early on and then decay rapidly (Figs 2C and S2A–S2C). Activated STAT1 proteins dimerize and translocate into the nucleus where they, at the transcriptional level, upregulate IRF-1 (Figs 2D and S2F) and subsequently several downstream M1 phenotypic markers (Figs 2E–2J and S2G); the time-course expression of these targets is more sustained over a prolonged period compared to that of phosphorylated STAT1. Activation of IFN-γ/STAT1 axis also induces the protein expression of two negative feedback regulators SOCS1 and SOCS3 (S2D–S2E Fig), which lasts rather briefly given their short half-lives [58]. In the meantime, IFN-γ signaling significantly inhibits the expression of miR-3473b (Fig 2K), which in turn would de-suppress its target PTEN (Fig 2L) to downregulate AKT activation. In addition, IFN-γ stimulation induces significant HIF-1α stabilization in macrophages even in normoxia (the induction is further boosted in hypoxia, Fig 2M), while its influence on HIF-2α expression is much less evident (S2I Fig).

#### IL-4-driven pathway

**Fig 3:**
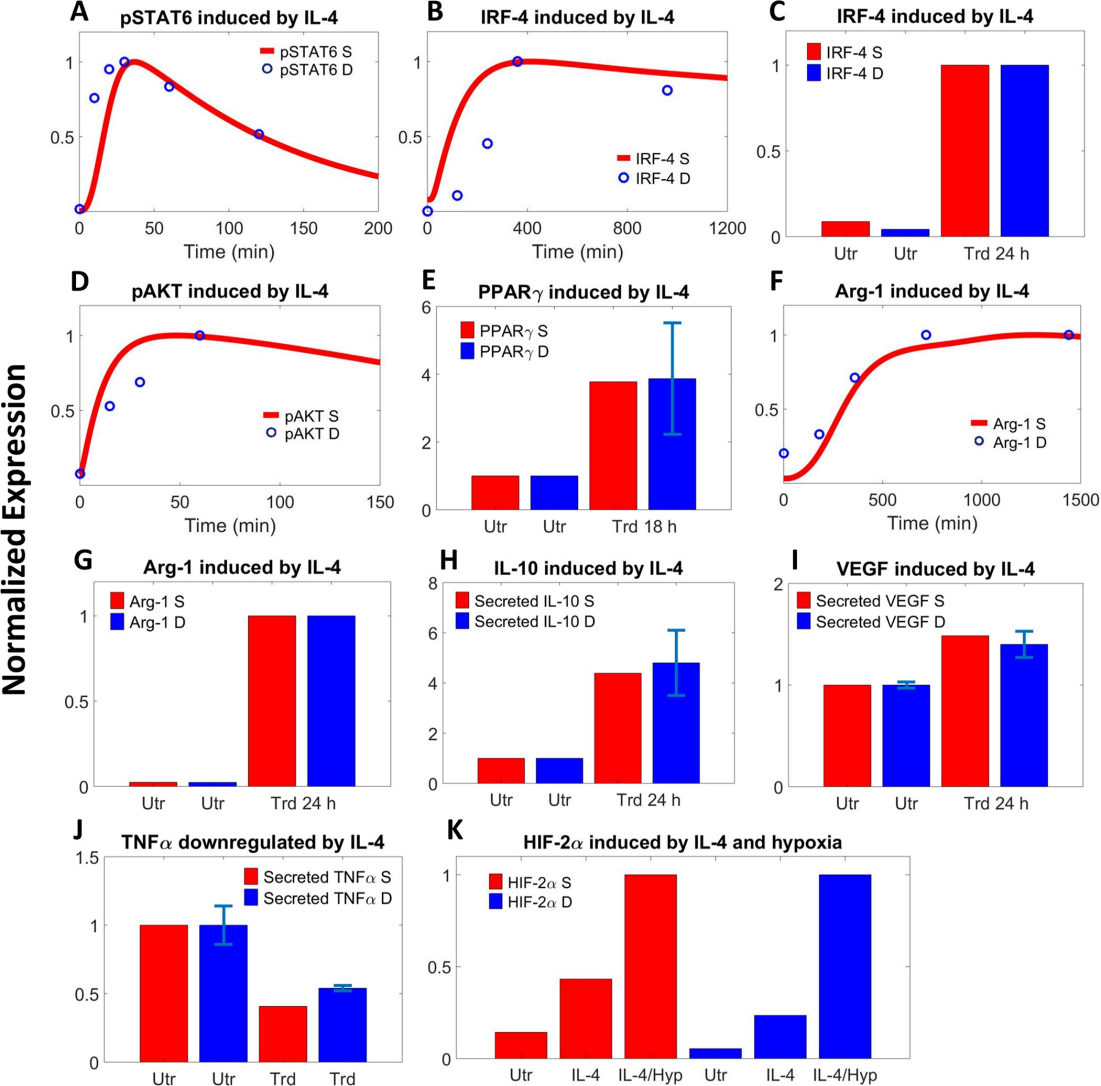
IL-4-mediated signaling controls macrophage phenotype. Comparison between model simulations and literature experimental data on IL-4 induced (A) STAT6 phosphorylation [65], (B-C) IRF-4 upregulation (time-course and at 24 h) [66, 67], (D) AKT activation [68], (E) PPARγ expression at 18 h [69, 70], (F-G) Arg-1 expression (time-course and at 24 h) [67, 71], (H) IL-10 secretion at 24 h [72], (I) VEGF secretion at 24 h [73], (J) downregulation of TNFα secretion at 24 h [72], and (K) HIF-2α stabilization (in response to IL-4 with or without hypoxia) [48]. (A-K) All literature data are measured in macrophage cell lines and values are for protein levels unless noted otherwise. Y-axes show normalized expression respectively (A, B, D, F: simulations and data are normalized to the maximum expression; C, G: normalized to the expression at 24 h post-treatment; E, H, I, J: normalized to the no-treatment/time 0 expression; K: normalized to the expression under IL-4 treatment with hypoxia). S–simulation, D–literature data, Utr–untreated, Trd–IL-4 treated, Hyp–hypoxia.

Similar to STAT1 under IFN-γ treatment, when macrophages are treated with high doses of IL-4, simulation and data both show early phosphorylation peaks followed by rapid dephosphorylation for intracellular STAT6 (Figs 3A and S3A–S3C); the dynamics of phosphorylated STAT6 within the nucleus also follows the trend (S3D Fig). Other signaling mediators activated by IL-4 such as IRF-4 (Figs 3B, 3C and S3E), PPARγ (Fig 3E) and AKT (Figs 3D, S3F and S3G), together with STAT6, will promote the expression and secretion of M2 markers including Arg-1 (Figs 3F, 3G, S3H and S3I), IL-10 (Figs 3H and S3J) and VEGF (Figs 3I and S3K). SOCS1, but not SOCS3, is a transcriptional target of STAT6 and is therefore upregulated transiently upon IL-4 stimulation (S3L Fig). IL-4 signaling can also downregulate M1-like features such as TNFα secretion (Figs 3J and S3M), and IL-4 selectively induces HIF-2α protein expression in normoxia and hypoxia (Fig 3K) with minimal impact on HIF-1α (S3N Fig).

#### Hypoxia-driven pathway

**Fig 4:**
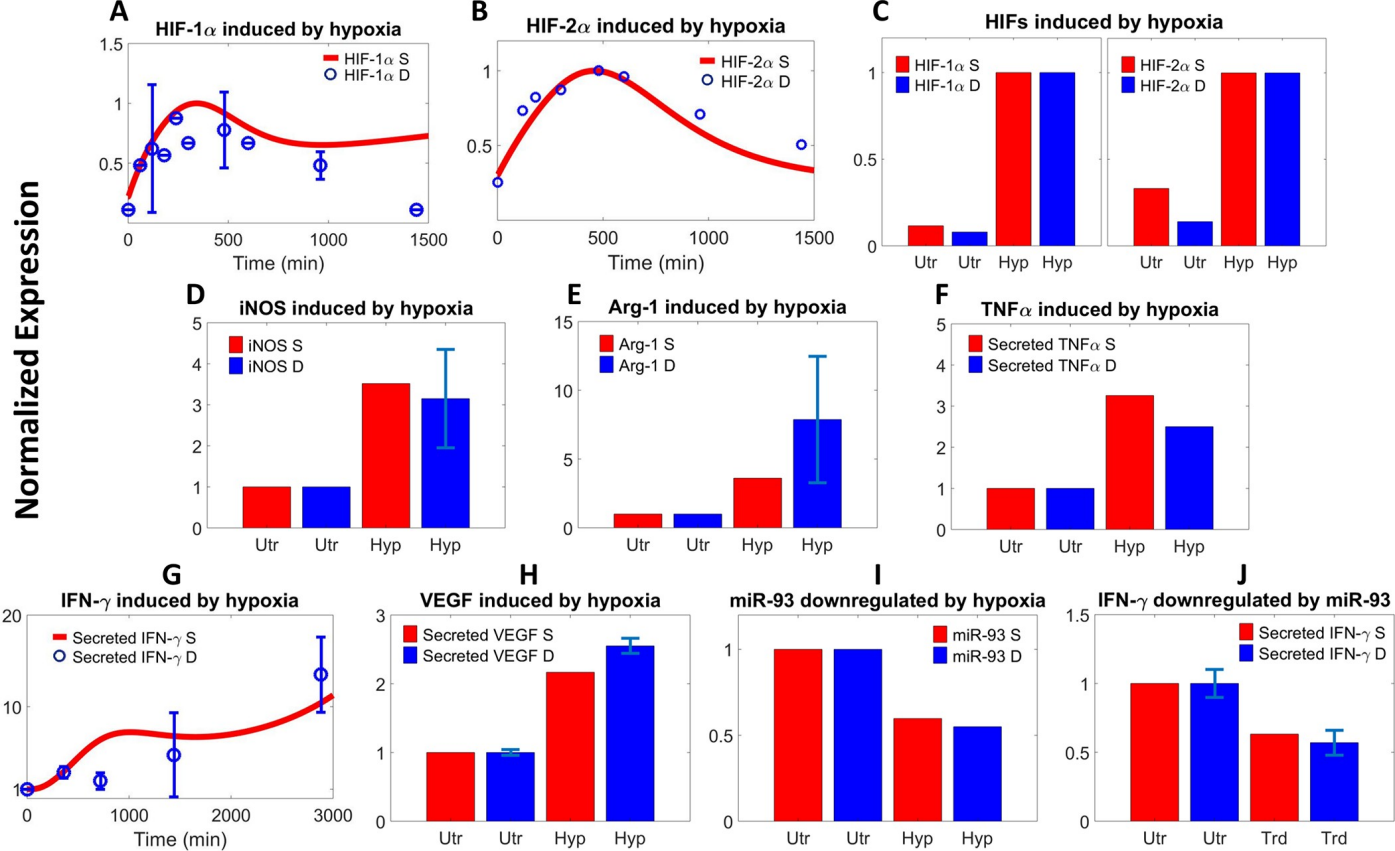
Hypoxia promotes M1 and M2 marker expression. Model simulation and literature experimental data from macrophages on hypoxia-induced (A) time-course stabilization of HIF-1α and (B) HIF-2α under 3% O_2_ [75, 76], (C) sustained stabilization of HIF-1/2α at 24 h under 0.5% O_2_ [77], (D) upregulation of iNOS and (E) Arg-1 proteins at 8 h under 1% O_2_ [78], (F) increase in TNFα secretion at 24 h under 0.3% O_2_ [79], (G) increase in IFN-γ secretion over time under 1% O_2_ [47], (H) increase in VEGF secretion at 24 h under 1% O_2_ [80], and (I) inhibition of miR-93 abundance at 12 h under 2% O_2_ [53]. (J) Enforced overexpression of miR-93 (see also S4G Fig) leads to decreased IFN-γ secretion at 12 h under 2% O_2_ [53]. (A-J) All literature data are measured in macrophage cell lines and results are for protein levels unless noted otherwise. Y-axes show normalized expression respectively (A, B: simulations and data are normalized to the maximum expression; C: normalized to the expression at 24 h under hypoxia; D-I: normalized to the normoxic/time 0 expression; J: normalized to the hypoxia-induced expression at 12 h without miR-93 mimic treatment). S–simulation, D–literature data, Utr–normoxia/untreated, Trd–treated with miR-93 mimic, Hyp–hypoxia, O_2_ –oxygen.

In hypoxia, HIF-1 and HIF-2 proteins are rapidly stabilized (Figs 4A–4C and S4A) due to decreased hydroxylation and degradation. The sustained expression of HIFs would lead to transcriptional activation and synthesis of both iNOS and Arg-1 (Fig 4D and 4E) in macrophages, and as described by the model, HIFs can significantly induce the production and secretion of pro-inflammatory cytokines TNFα and IFN-γ (Fig 4F–4G) as well as the pro-angiogenic factor VEGF (Figs 4H and S4D) through both direct and indirect mechanisms. The drastic rise in intracellular HIFs would also trigger feedback mechanisms such as increased PHD expression (S4B Fig) to prevent unrestrained HIF-mediated signaling. For hypoxia-driven post-transcriptional regulation, an example is that hypoxia can cause downregulation of miR-93 (Fig 4I) in macrophages which in turn would free IRF-9 from translational inhibition (S4E Fig), and IRF-9 plus hypoxia-induced IRF-1 (S4C Fig) can subsequently potentiate the expression of IRG-1 (S4F Fig), which is a highly expressed gene particularly in M1-like, pro-inflammatory macrophages [74]. Following this axis, introduction of miR-93 mimic would decrease the cellular expression of IRF-9 which would lead to downregulation of IRG-1 (S4G and S4H Fig). Overexpression of miR-93 by mimic transfection is also capable of suppressing the hypoxia-driven secretion of pro-inflammatory cytokines such as IFN-γ and TNFα (Figs 4J and S4I) by macrophages.

### Pathway feedback through SOCS proteins and IL-4/IFN-γ mutual antagonism

**Fig 5:**
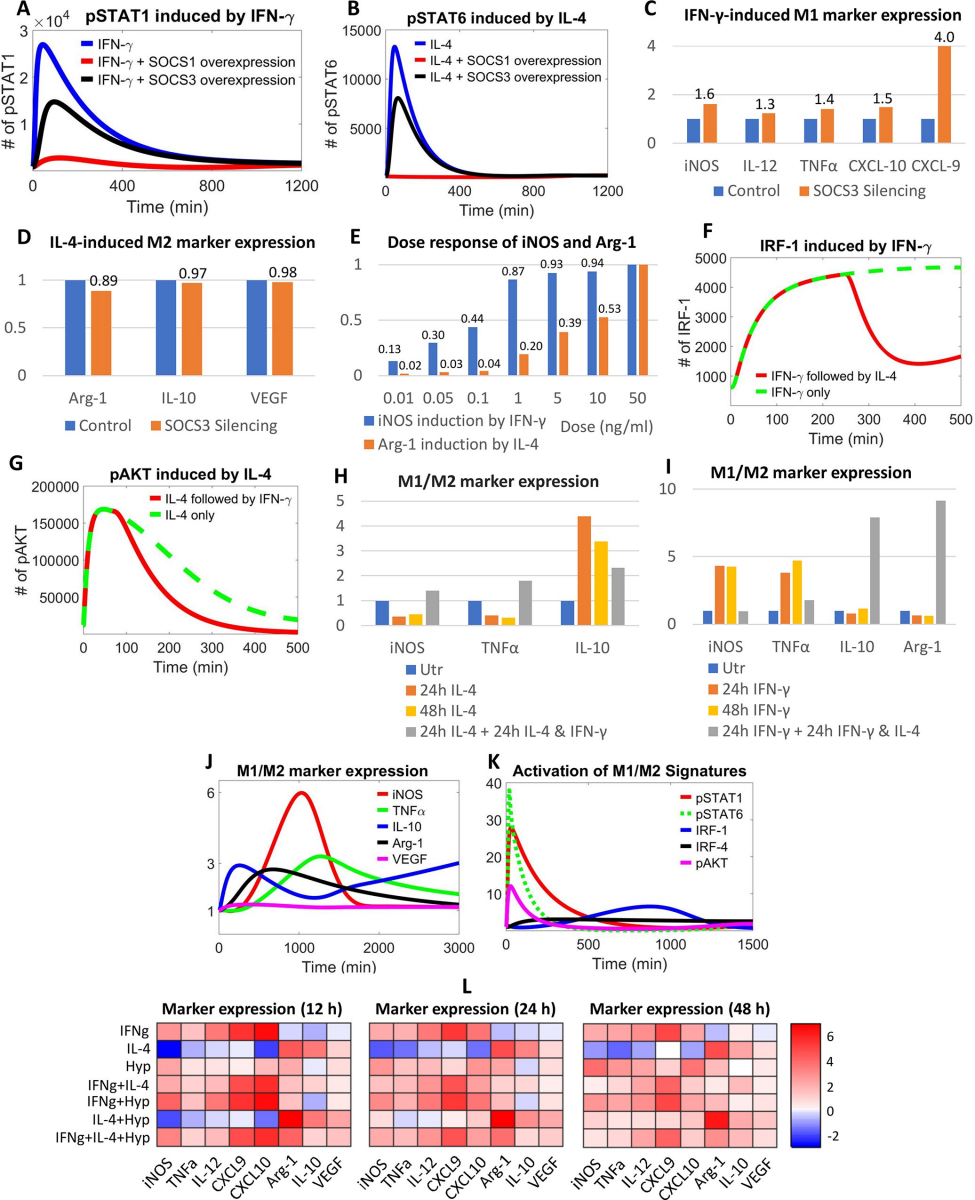
Pathway feedbacks and cross-talks in M1-M2 regulatory network. Overexpression of SOCS1 and SOCS3 in macrophages can downregulate activation of (A) STAT1 by IFN-γ and (B) STAT6 by IL-4. Silencing of SOCS3 promotes (C) IFN-γ-induced M1 marker expression while it minimally affects (D) IL-4-induced M2 marker expression (relative fold changes are labeled). (A-D) Overexpression is modeled as 50x initial level with normal (1x) production, and silencing is modeled as 0 initial level with 0 production. (E) Simulated dose response of iNOS and Arg-1; relative protein levels measured at 12 h are plotted and labeled (the baseline condition is represented by the 0.01 ng/ml case). (F) Upon IFN-γ stimulation followed by the addition of IL-4 (at 4 h), cellular IRF-1 level is downregulated compared to IFN-γ only; (G) Upon IL-4 stimulation followed by the addition of IFN-γ (at 1 hr), cellular activation of AKT is downregulated compared to IL-4 only. (H) The addition of a second stimulus IFN-γ (after 24 h of IL-4 stimulation) would antagonize the expression pattern of M1 and M2 markers induced by IL-4 (see also S5G Fig). (I) Similarly, IL-4 added after 24 h of IFN-γ stimulation would antagonize the marker expression pattern induced by IFN-γ. When macrophages are stimulated with IFN-γ and IL-4 simultaneously, the simulated expression of (J) M1 and M2 markers as well as (K) the activation of a number of M1 and M2 signature proteins (see also S5I Fig) are collectively induced with distinct temporal profiles. (L) Dynamic protein expression patterns (after 12, 24 and 48 h of stimulation) of M1 and M2 markers in macrophages under seven different stimulation conditions (A+B means simultaneous stimulation, expression levels are normalized to the untreated/time 0 levels and then log2 transformed). (A-L) All simulation results are protein levels (except CXCL10 is mRNA level). (C-E, H-K) Y-axes show relative expression respectively (C-D, H-K: normalized to untreated/control/time 0 levels; E: normalized to maximum levels at 50 ng/ml). Simulated treatment doses are 10 ng/ml IFN-γ and 10 ng/ml IL-4 for (A-D), 10 ng/ml IFN-γ and 20 ng/ml IL-4 for (F-G), 20 ng/ml IFN-γ and 20 ng/ml IL-4 for (H-I), 10 ng/ml IFN-γ and 5 ng/ml IL-4 for (J-L). Utr–untreated, hyp–hypoxia (2% oxygen for L).

SOCS proteins are considered key regulators of JAK/STAT signaling and therefore contribute profoundly to the dynamic polarization of macrophages [81]. Fig 5A and 5B show that overexpression of SOCS1 and SOCS3 can potently suppress the activation (e.g. phosphorylation) of STAT1 (when stimulated with IFN-γ) and STAT6 (when stimulated with IL-4), and model simulations indicate that SOCS1 is a stronger inhibitor than SOCS3 in both cases, which agrees qualitatively with previous experimental observations [24, 82]. Silencing either SOCS1 or SOCS3, on the other hand, will selectively augment STAT1 and STAT6 activation (S5A and S5B Fig). These simulations also suggest that different levels of SOCS proteins seem to affect the peak level but not the long-term activation of STAT1 and STAT6, except for the case of SOCS1/3 silencing in IL-4-induced STAT6 activation (S5B Fig); a possible mechanistic explanation is that both SOCS1 and SOCS3 are inducible by IFN-γ and thus act redundantly as regulators of STAT1, while only SOCS1 is inducible by IL-4 so it becomes the dominant regulator of STAT6 upon IL-4 stimulation [24]. Following this reasoning, we simulated the influence of SOCS3 deficiency on the production of several macrophage phenotype markers (Fig 5C and 5D). The absence of SOCS3 markedly increases M1 marker expression in response to IFN-γ (Fig 5C), while changes in the level of M2 markers in response to IL-4 are relatively minimal (Fig 5D), which is consistent with experimental findings by Qin et al. that showed macrophages without SOCS3 are likely more sensitive to M1 stimuli (e.g. LPS, IFN-γ) but not the M2 phenotype inducer IL-4 [83]. Additional simulations on SOCS1 suggest that knockdown of SOCS1 would boost IL-4-driven M2 marker expression (e.g. Arg-1) which agrees with previous macrophage studies [24, 84], but it could have a mixed impact on the expression of M1 markers upon IFN-γ stimulation (S5C and S5D Fig). We also explore the potential utility of our model beyond *in vitro* conditions, since the cytokine doses used to stimulate macrophages *in vitro* often vastly exceed the physiological tissue concentrations. The dose response simulations using iNOS and Arg-1 as examples (Fig 5E) suggest evident induction of iNOS (by IFN-γ) and Arg-1 (by IL-4) in macrophages (compared to baseline) even under very low levels of cytokine stimulation (high pg/ml to low ng/ml range), which is consistent with prior experimental findings [15, 85] and this dose range also agrees well with the reported tissue concentrations of cytokines in health and disease [86]. Therefore, our model can also be used to offer mechanistic insights for the investigation of macrophage polarization and function *in vivo*.

In addition to SOCS proteins and STATs, the mutually antagonistic regulation of macrophage polarization by IL-4 and IFN-γ involves the tuning of several other key signaling modulators. According to model simulations, induction of IRF-1 expression in IFN-γ-stimulated macrophages would be strongly downregulated by the addition of IL-4 (Fig 5F), which is supported by literature evidence [87]. Similarly, AKT activation (e.g. phosphorylation) would be impaired by IFN-γ in IL-4-polarized macrophages (Fig 5G). It is also suggested that despite the antagonistic effect on IRF-1 and AKT by IFN-γ/IL-4, there are no obvious changes in the respective activation of STAT1 (by IFN-γ versus IFN-γ followed by IL-4) and STAT6 (by IL-4 versus IL-4 followed by IFN-γ) (S5E and S5F Fig). Furthermore, model simulations (Figs 5H, 5I and S5G) show that delayed exposure to IFN-γ could antagonize the expression pattern of M1 and M2 markers that are induced by IL-4 in the first place (similarly, delayed exposure to IL-4 can antagonize the effect of IFN-γ on marker expression), which is corroborated at the mRNA level by our experimental data in THP-1 cells (S5H Fig) and aligns with the argument that macrophage polarization is dynamic and reversible [1, 25]. In addition, when a macrophage is stimulated with IFN-γ and IL-4 simultaneously, Arg-1 and iNOS are both upregulated, but the duration of high iNOS expression is relatively short while Arg-1 expression is elevated in a more prolonged manner; the changes in TNFα production seem to be opposite to that of IL-10 throughout the simulated timespan, and VEGF remain modestly upregulated (Fig 5J). Simulations from our systems-level model suggest that, when exposed to a combination of M1 and M2 stimuli (e.g. IFN-γ and IL-4), macrophages at the single-cell level tend to activate multiple signaling modules (Figs 5K and S5I) and upregulate both M1 and M2 markers (Fig 5J) with differential expression strengths and temporal patterns, instead of being polarized exclusively toward one end or the other. We further explored the cell level response of macrophages in seven different conditions with single and combined stimulation (Fig 5L), and the simulated expression of a set of M1 and M2 markers suggest that not only the different stimulation strategies but also the temporal aspect itself (e.g. time) are determinants of the observed dynamic phenotypic variability in the response, which is also reflected in the activation patterns of various M1 and M2 signature transcription factors (S5J Fig). This could have important implications for the mechanistic understanding of the macrophage polarization spectrum *in vitro* and particularly *in vivo*, since physiological and pathological environments usually contain a multitude of M1 and M2 drivers, thus the dynamic profiles of not just one or two but an array of relevant M1 and M2 markers should be systematically taken into account in order to fully evaluate the phenotypic function of macrophages under such conditions.

### Model sensitivity analysis and potential strategies to direct therapeutic macrophage polarization in diseases

**Fig 6:**
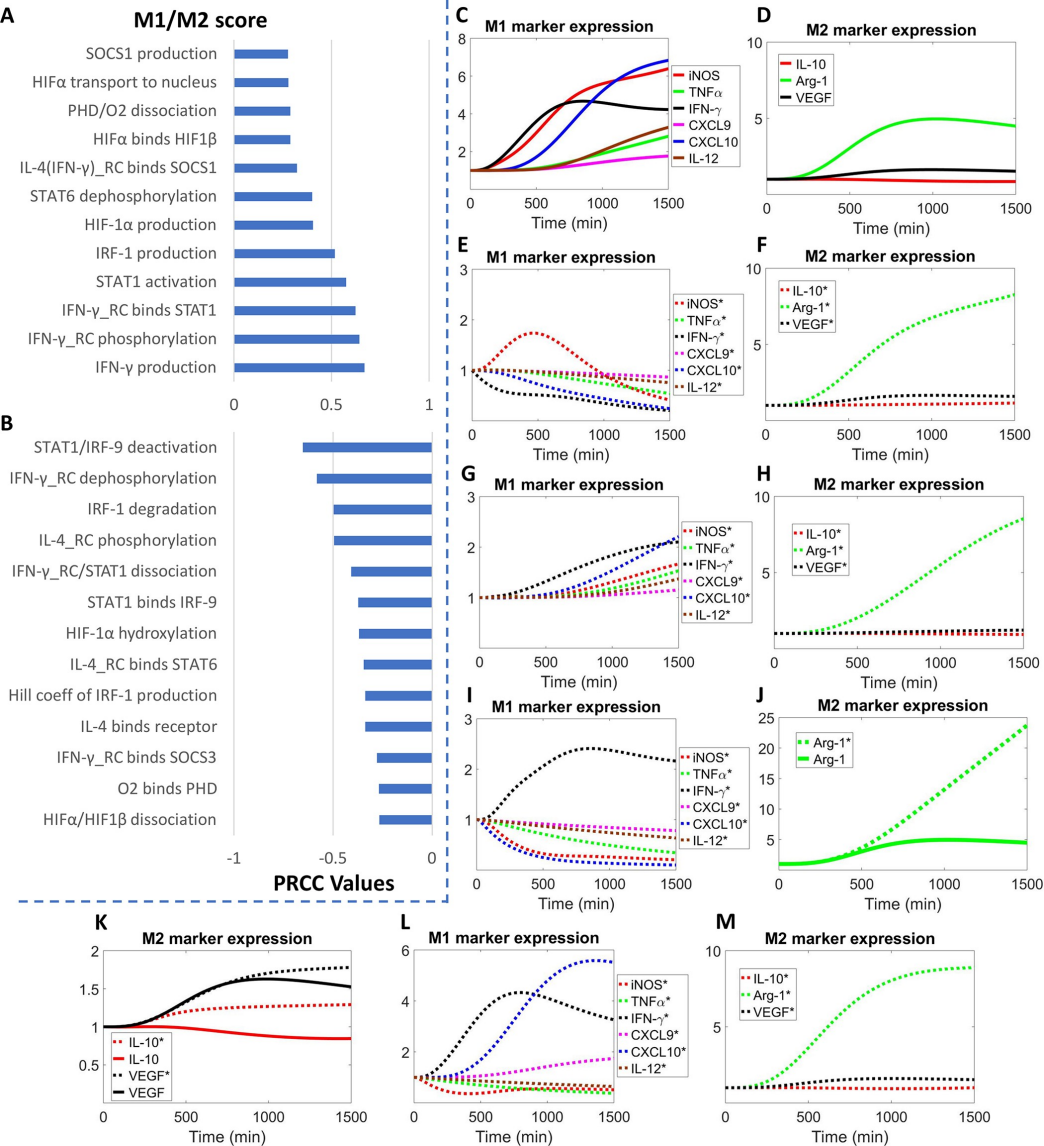
Global sensitivity analysis and simulated therapeutic strategies to repolarize macrophages in hypoxia. (A-B) Sensitivity indices (top 25 positive and negative PRCC values with p<0.05) of model parameters that control M1 and M2 marker expression in terms of the M1/M2 score (a ratio-based estimate of M1 phenotypes relative to M2 phenotypes, see Materials and Methods for more details) in hypoxia (2% O_2_). In the parameter descriptions, ‘X_RC’ means receptor complex formed by ligand X, receptor and JAK, ‘X/Y’ means complex formed by X and Y. Simulated time-course expression of M1 and M2 markers when macrophages are subjected to (C-D) hypoxia, (E-F) hypoxia with IFN-γ inhibition, (G-H) hypoxia with HIF-1α inhibition, (I-K) hypoxia with STAT1 inhibition, and (L-M) hypoxia with IRF-1 inhibition. Inhibition of IFN-γ, HIF-1α and IRF-1 is simulated by setting the respective production rates to 10% of their original values (STAT1 inhibition is simulated as a 90% decrease in the binding rate between STAT1 and activated IFN-γ receptor complex). Species name denoted with * means expression in hypoxia plus treatment (species name without * means expression in hypoxia alone). (C-M) Marker expression levels are normalized to their respective t = 0 values (e.g. normoxia, unstimulated). (A-B) More details about the parameters listed can be found in S1 Table using the labels (positive–*k127*, *kf63*, *kr70*, *kf64*, *kf17*, *k33*, *k61*, *k77*, *k45*, *kf44*, *kf42*, *k37*; negative–*k99*, *kr42*, *k78*, *kf8*, *kr44*, *kf95*, *k71*, *kf13*, *ka77*, *kf7*, *kf52*, *kf70*, *kr64*; order is from top to bottom as displayed). (C-M) All simulation results are protein levels (except CXCL10 is mRNA level).

In order to identify the parameters that most significantly influence the model outputs of interest (e.g. M1 and M2 marker expression) and thereby propose novel targets that can therapeutically repolarize macrophages under disease conditions, we performed global sensitivity analysis (see Materials and Methods section for more details) using the PRCC algorithm (partial rank correlation coefficient) [88]. We first focus on PAD, a highly-prevalent cardiovascular problem characterized by reduced blood flow and ischemia that most commonly affects the lower limbs [89]. Under *in vitro* hypoxia, which is a plausible reflection of the adverse cellular environment found in ischemic tissue in PAD [53, 90], sensitivity analysis shows that the most influential parameters are closely related to the activation (mostly through direct control of phosphorylation, binding and synthesis) of IFN-γ/STAT1/IRF-1 axis, IL-4/STAT6 axis and O_2_/HIF-1 axis, as expected (Fig 6A and 6B). Since hypoxia can induce M1-like phenotypes in macrophages which may thwart angiogenesis and perfusion recovery in the ischemic limb tissue [53, 91], we simulated several therapeutic interventions that are potentially translatable based on the processes described by the high-sensitivity parameters, namely to inhibit the synthesis (through miRs or siRNAs) of IFN-γ, IRF-1, HIF-1α and activation of STAT1 (through selective inhibitors) in order to revert the M1-like phenotypes. Our simulations show that hypoxia alone can upregulate the expression of a number of M1 and M2 markers (Fig 6C and 6D), while inhibition of either IFN-γ (Fig 6E and 6F), HIF-1α (Fig 6G and 6H) or STAT1 (Fig 6I–6K) can potently limit the upregulated expression of all M1 markers and further boost Arg-1 production in hypoxia. The overall impact of IFN-γ inhibition is stronger than that of HIF-1α inhibition, and in the latter scenario VEGF production is reduced (Fig 6H) compared to control (hypoxia only, Fig 6D), while inhibiting IFN-γ (S6A Fig) or STAT1 (Fig 6K) can both promote IL-10 and VEGF production in hypoxia as a result of enhanced baseline activation of IL-4 pathway (S6B and S6C Fig) due to downregulation of SOCS proteins (S6D and S6E Fig). In contrast, simulations suggest that inhibition of IRF-1 can only suppress part of the six M1 markers examined (in addition to its positive influence on Arg-1 production) (Fig 6L and 6M), which can be explained by the topology of IRF-1 within the M1-M2 signaling network. Although the potential functions of IFN-γ/STAT1/IRF-1 axis have not been assessed so far in experimental PAD models, prior research has reported that PAD patients have significantly higher levels of circulating IFN-γ, and that silencing of STAT1 as well as IRF-1 has been associated with increased outcome and recovery following ischemic injury in other vital organs (e.g. kidney, brain, liver) [92–95]. HIF-1α is an inducible gene following tissue ischemia in PAD and our model suggests that it can also contribute to the pro-inflammatory, M1-like phenotypes in macrophages [96, 97]. Therefore, its regulation of the M1-like macrophage response, in addition to its well-established effect on cellular VEGF production, might provide another mechanistic explanation for the clinical failure of prior therapies that aimed to treat PAD by overexpressing HIF-1α [98, 99].

**Fig 7:**
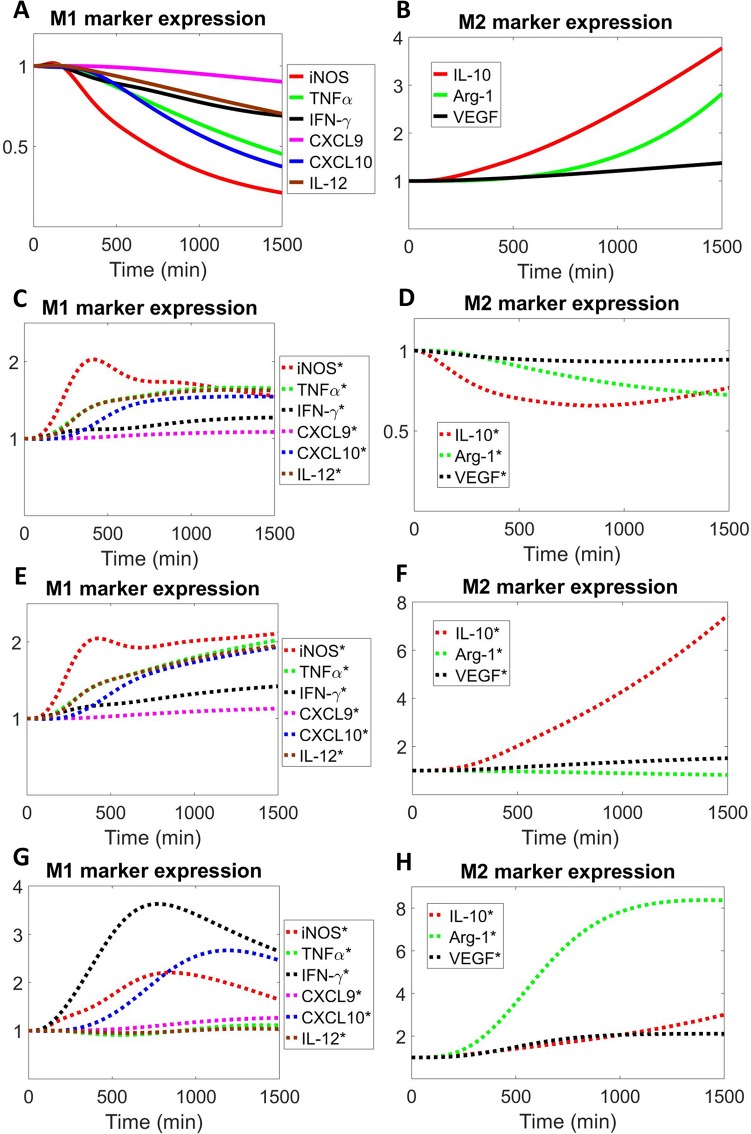
Targeting IL-4 signaling axis in macrophages in tumor. Simulated time-course expression of M1 and M2 markers when macrophages are subjected to (A-B) high IL-4 production (10x of original value), (C-D) high IL-4 production with IL-4/receptor blockade (90% decrease in the binding rate between IL-4 and its receptor), (E-F) high IL-4 production with STAT6 inhibition (90% decrease in the binding rate between STAT6 and activated IL-4 receptor complex), and (G-H) high IL-4 production with PHD inhibition (90% decrease in the binding rate between PHD and O_2_). Species name denoted with * means expression in high IL-4 production plus treatment (species name without * means expression in high IL-4 production alone). (A-H) Marker expression levels are normalized to their respective t = 0 values (e.g. normoxia, unstimulated). All simulation results are protein levels (except CXCL10 is mRNA level).

We also explored different strategies *in silico* to elicit pro-inflammatory macrophage polarization when IL-4 levels are abnormally elevated, a feature often observed in the tumor microenvironment and associated negatively with patient survival [100–102]. Since high IL-4 production and signaling skew macrophages toward M2-like, anti-inflammatory phenotypes which could be pro-tumorigenic (Fig 7A and 7B), based on the parameter sensitivities calculated in this pathological scenario (S7A and S7B Fig), several therapeutic targets were compared in terms of their effects on the promotion of M1 markers and inhibition of M2 markers. Direct blockade of the interaction between IL-4 and its receptor can most effectively achieve this goal (Fig 7C and 7D) by completely reverting the original expression pattern (decreased M1 markers plus increased M2 markers) induced by high IL-4 production. However, targeting the downstream nodes, namely STAT6 (Fig 7E and 7F), HIFs (through PHD) (Fig 7G and 7H) and AKT (S7C and S7D Fig), failed to exert consistent repolarizing effects on the expression of both M1 and M2 markers, which can be mechanistically explained by the pathway topology as well as the activation of compensatory signaling (S7E Fig). Taken together, our model simulations suggest the value of targeting IL-4 axis in tumor, especially the therapeutic blockade of IL-4/receptor interaction (e.g. inhibiting IL-4Rα, a receptor subunit utilized in both IL-4 and IL-13 signaling) as a potential approach to modulate macrophage-mediated immune response to combat tumor progression.

As a continuation of the sensitivity analysis, we further performed uncertainty analysis using bootstrap procedures (S8 Fig) with a focus on the identifiability of a subset of model parameters that have the highest sensitivity values [103]. With the large amount of experimental data used in model calibration and the relatively small degree of freedom allowed, the results suggested relatively good clustering of those high-sensitivity parameters (S8 Fig). However, we acknowledge that practical unidentifiability associated with the model parameters would likely emerge once we take into account all the parametric freedom and uncertainty embedded in the mechanistic details of our model (with the current dataset), and thus additional experimental measurements on the rates of relevant reaction processes and trajectories of model species behaviors would further empower the model predictions.

## Materials and methods

### Model formulation and simulation

The model was constructed based on ordinary differential equations (ODEs) with a total of 80 model nodes (from the 34 “unique” species) and 130 reactions (more details about reaction descriptions, equations, parameter and initial condition values are summarized in S1 and S2 Tables). For IL-4 and IFN-γ receptor dynamics, we coarse-grained the entire process into key sequential steps such as receptor ligation, phosphorylation, receptor complex internalization (only for IL-4 but not IFN-γ), degradation and recycling [104, 105]. For the activation cascades of STAT1 and STAT6, both proteins undergo phosphorylation, dimerization and dimer translocation (from cytoplasm into nucleus) in the model in order to transcriptionally regulate downstream genes. For SOCS-mediated feedback, SOCS1/3 can bind with both internalized and uninternalized receptor complexes to induce JAK inhibition and sequestration (followed by JAK degradation) as well as receptor complex degradation [106]. AKT activation is modeled as a one-step process downstream of IL-4 receptor activation, and in the current version we do not distinguish between the effects by different phosphorylation sites (this also applies to STAT1 and STAT6). The current hypoxia module is based on a previous model and we further simplified the PHD activation and HIF hydroxylation steps, added HIF de-ubiquitination, and lumped PHD and FIH (factor inhibiting HIF-1) into one species (under transcriptional control by HIF-1/2) [44]. For miR functions, we simplified the description from previous models so that miRs will directly bind mRNAs and induce target mRNA degradation [107–109]. Additional rationale regarding model formulation is described in S1 Protocol. All model data (e.g. reaction rules, species and nodes, parameters values) are compiled in MATLAB SimBiology Toolbox (MathWorks, Natick, MA) and we used the ode15s solver in MATLAB for model simulations. All model reactions are encoded in deterministic mass action and Hill-type algebraic kinetics; although stochasticity could play an important role in gene transcription which is part of the model, we have demonstrated that the simulated average behaviors of model outputs of interest (e.g. expression of various proteins) agree reasonably well with experimental data, therefore we believe that the deterministic approach would be sufficient for our major purposes. For the comparisons between simulation and experimental data in response to IL-4/IFN-γ stimulation at different doses, we calculated average exposure per cell (using dose information from the experimental source, plus molecular weight and unit conversion information supplied by R&D Systems, with the assumption that macrophages are plated at a million cells per ml of culture media [110]) and set the computed values as the new initial conditions for IL-4/IFN-γ. For experimental sources that used a combination of IL-4 and IL-13, we made a simplification by assuming functional equivalence between the two cytokines and considered both as IL-4 during model calibration. We used ImageJ software (NIH) to perform blot densitometry analysis and other image measurements in order to quantify the experimental data which are subsequently used in model calibration. Model SBML code is also submitted with the Supporting Information to ensure reproducibility.

### Model initialization and calibration

Since our mechanistic model is the first kinetics-based computational platform to describe the complex dynamics of macrophage polarization under the influence of M1 and M2 inducers and hypoxia, we conducted extensive literature search to collect relevant experimental data for parameter estimation and model calibration. We used several sources that globally measured absolute copy numbers and half-lives of different proteins and RNAs (in macrophages and other cell lines) to confine the initial conditions and degradation rates of most model species [111–115], and this is also complemented by various literature studies that individually measured the properties of specific proteins that are described in the model. In addition, we also derived the numerical values for a handful of parameters associated with other reactions such as receptor binding, protein phosphorylation and transport from published quantitative data. A detailed summary of all the parameter and initial condition values with corresponding sources is shown in S1 and S2 Tables.

As shown in Figs 2–4 and S2–S4, we were able to compile over 70 pieces of relevant quantitative experimental data from literature (summarized in S3 Table) that comprehensively described the kinetics of almost all the functionally “unique” species in the model (for data display, means ± SEM or SD were calculated and used when possible). While all these data are fed into model calibration simultaneously to ensure that they are in good agreement with their simulation counterparts, a second check is that when the model is simulated under the control condition (normoxia without externally added stimuli) to acquire the physiological states of unpolarized macrophages (the initial conditions), the final output copy numbers at steady state for all the “unique” species should be within reasonable ranges (e.g. 0.5x-2x) of literature reported values (as described above). We used the *patternsearch* function provided in MATLAB for model parameter optimization (the algorithm runs iteratively to minimize the weighted sum of square errors between simulations and experimental data as well as to pass the initial condition check). For the calculations of square errors, experimental datasets are normalized and averaged to obtain mean and standard deviation values when possible. More technical detail regarding model calibration is described in S1 Protocol.

### Model sensitivity and uncertainty analyses

Sensitivity analysis was performed using the PRCC method [88]. We introduced the M1/M2 score which is the multiplication of six M1 marker levels divided by the multiplication of three M2 marker levels to quantify the relative changes within the M1-M2 spectrum over time (as outlined in Figs 6 and 7), and the M1/M2 score (e.g. evaluated at 24 hours) was used as the output of interest for the sensitivity calculations together with a sample size of 5000 for the results shown in Figs 6A, 6B, S7A and S7B. Bootstrapping was used to resample all the calibration data and we re-optimized the model repeatedly to obtain 50 sets of new parameter estimates. A subset of 11 parameters was chosen for the 50 optimization runs (based on the overall pattern of parameters with the highest sensitivity indices in the cases of IL-4 stimulation, IFN-γ stimulation, and hypoxia) (S8 Fig). More technical detail regarding model sensitivity analysis and uncertainty quantification is described in S1 Protocol.

### THP-1 polarization and quantitative reverse transcription PCR

THP-1 cells were maintained at 37°C in RPMI supplemented with 10% FBS, L-glutamine, penicillin/streptomycin, and 55 μM β-mercaptoethanol. For polarization experiments, 1*10^6^ cells were transferred into wells of a 6-well plate in 5 ml of growth media and induced into macrophages with 150 nM phorbyl-12-myristate-13-acetate (PMA; Sigma). After 24h the media was replaced with fresh growth media without PMA for another 24 hours. The media was then replaced with additional growth media containing polarization factors for the indicated amounts of time. M1-type polarization was induced using 20 ng/ml IFN-γ (Peprotech) and M2-type with 20 ng/ml IL-4 (Biolegend). For dual treatments, cells were treated with the first factor for 24 hours followed by the addition of the second for another 24 hours without changing media. The RNA was then extracted using an RNeasy kit (Qiagen) according to the manufacturer’s protocol. For lysis, cells were lysed in 350 μl of the supplied kit lysis buffer per well and transferred to QIAshredders (Qiagen) before continuing with the rest of the protocol. cDNA was synthesized from 750 ng of RNA using the High Capacity cDNA Reverse Transcription kit (Applied Biosystems) according to the manufacturer’s protocol. qPCR was performed using the Taqman Gene Expression Mastermix (Applied Biosystems), 25 ng of cDNA, and Taqman assay (Applied Biosystems) probes with FAM labels described below. Data were collected and analyzed using the Quantstudio 12K Flex instrument and software with untreated cells as treatment controls and GAPDH and β-actin as internal expression controls. The Taqman assays that were used in this study are as follows: IL-10 (Assay#: Hs00961622_m1); TNF (Assay#: Hs00174128_m1); GAPDH (Assay#: Hs02786624_g1); β-actin (Assay#: Hs01060665_g1).

## Discussion

In this study, we have developed and presented the first kinetics-based, mechanistic multi-pathway computational model of macrophage polarization in the context of hypoxia and canonical M1-M2 stimulation (IFN-γ, IL-4). Within this scope, our systems-level integrative model based on the current knowledge is calibrated extensively against quantitative experimental data and captures the dynamical regulation and expression of essential transcription factors and markers that are associated with macrophage phenotypes in response to physiological and pharmacological perturbations. Motivated by the fact that prior attempts that used systems-level approaches to model multi-pathway cancer signaling have generated crucial insights for the integrative understanding of cancer cell dynamics and drug targeting [116, 117], we consider the current model an important first step toward a more comprehensive characterization of a “virtual macrophage” assembled through systems-level modeling techniques that is able to suggest translational insights for cardiovascular and cancer research. The goal of the “virtual macrophage” is to quantitatively describe the continuous spectrum of macrophage polarization in terms of cross regulation and activation within a network of multiple pathways leading to time-dependent up- and down-regulation of an array of phenotypic markers which are able to reflect real macrophage physiology. Therefore, our current model setup can be expanded to further incorporate the autocrine effects by other key cytokines secreted by macrophages (in addition to IFN-γ and IL-4 as described in this work). For example, IL-1β and TNFα are both macrophage products that can potentially function through autocrine and paracrine signaling to regulate macrophage-mediated inflammatory responses in disease settings [118–120]. Activation of pro-inflammatory pathways (e.g. TLR4, toll-like receptor 4) in macrophages can also trigger delayed synthesis of IL-10 through sequential signaling and this could negatively affect the production of various pro-inflammatory cytokines in the long run [28, 121]; furthermore, IL-10 can induce its own production through the autocrine IL-10/STAT3 axis which could help to sustain an immuno-suppressive phenotype [122]. Recently, it has also been discovered that the pro-angiogenic factor VEGF may contribute to M2-like polarization through receptor-mediated signaling (e.g. via VEGFR1) on macrophages, and that the two isoforms of VEGF-A (165a and 165b) can differentially regulate macrophage phenotypes to influence perfusion recovery in PAD [91, 123]. Such evidence again points to the demanding mechanistic complexity of macrophage polarization at the cell level, which suggests that a systems-level description of macrophage signaling pathways and marker regulation, like we proposed through this work, should be the appropriate angle to devote future modeling efforts to provide a better understanding of the therapeutic values behind the full M1-M2 spectrum. In addition, a few computational models that focus on macroscopic, tissue-level macrophage dynamics and phenotypic patterns have also been developed, which are ideal *in silico* platforms that can be potentially merged with and enriched by the mechanistic insights from our multi-pathway model to demonstrate greater model utilities in the investigation of macrophage-disease interactions [124, 125].

While we formulated our model based on the decades of scientific knowledge derived from experiments done in macrophages in general, it is important to recognize that different macrophage cell lines are far from being equivalent with respect to their genetic background and innate cellular dynamics. Macrophages from human and mice are known to differ in their signature genes that represent the canonical M1 and M2 phenotypes (e.g. the controversy on iNOS and Arg-1 expression in human versus mouse macrophages) [126]. Macrophages derived from BALB/c and C57BL/6 mice are shown to secrete vastly different amounts of IFN-γ at rest and they also respond differently to IFN-γ and LPS in terms of the induction strengths and temporal trends of iNOS production [127, 128]. Likewise, common human macrophage cell lines such as monocyte-derived macrophages, THP-1, and U937 cells can show inconsistent marker expression patterns (e.g. increased, decreased, unchanged) following canonical M1 and M2 stimulation [129, 130]. In addition, agents such GM-CSF (granulocyte-macrophage colony-stimulating factor) and M-CSF (macrophage colony-stimulating factor) which are widely used in experimental preparations for monocyte-to-macrophage differentiation and proliferation are able to pre-condition macrophages towards amplified M1 and M2 responses respectively [131]. Tissue-resident macrophages, compared to monocyte-derived macrophages, may also respond differently to cellular perturbations given their distinct functional states shaped by the tissue microenvironments [132]. Therefore, given the above reasoning and the fact that the majority of our model calibration data were obtained from mouse bone marrow derived macrophages, it makes sense to acknowledge that sometimes the qualitative aspects of our model simulations might weigh more than the predicted quantitative fold changes, especially when the simulations are tested experimentally across different types of macrophages. Still, our mechanistic model is formulated in a way that can be easily adapted to accommodate the genetic background of more than one macrophage cell line, provided that the cell-line specific gene and protein expression data are available.

Our model included two signature molecular species of macrophages metabolism that have been previously associated with the M1-M2 phenotypes (itaconate– M1-like, PPARγ– M2-like) [133]. Although the current model, given its scope, considered both species primarily as marker outputs downstream of IFN-γ and IL-4 with limited regulatory functions, research has shown that they do play critical roles in macrophage metabolic processes to influence phagocytosis and inflammatory cytokine production [134]. Recent studies have discovered that itaconate, besides its pro-inflammatory properties in bacterial infection and hypoxic settings, can exert anti-inflammatory effects in M1-like macrophages by limiting succinate oxidation, a critical step in the citric acid cycle, to inhibit mitochondria ROS production and downstream M1-like marker expression; itaconate can also induce cysteine alkylation and activate Nrf2 (nuclear factor erythroid 2-related factor 2), a sensor of oxidative stress, to suppress transcription of pro-inflammatory genes in macrophages [53, 135–137]. On the other hand, accumulation of PPARγ in response to IL-4 can affect the production of itaconate and various macrophage cytokines, presumably through the control of glutamine metabolism and other signal transduction mechanisms that have not yet been fully elucidated [133]. Furthermore, the downstream metabolic products regulated by itaconate such as succinate and ROS can also modulate the stabilization of HIFs through distinct mechanisms [57, 138], which again connects the macrophage metabolic reprogramming initiated by itaconate with hypoxia- and HIF-mediated signal transduction. Taken together, more knowledge needs to be gained in order to fully decode the mechanistic wiring between macrophage metabolism and phenotypic M1-M2 signal transduction, and we believe that our model as well as future efforts that further expands our work (e.g. the “virtual macrophage”) can potentially speed up this process by efficient hypothesis generation and testing in parallel with targeted experimental validation and feedback.

## Supporting information

S1 File Compiled file of all supporting information documents. (PDF)

S1 Fig Complete model diagram with all nodes and reactions. (PDF)

S2 Fig (A-I) Additional model calibration data of IFN-γ-driven pathway.
(PDF)

S3 Fig (A-N) Additional model calibration data of IL-4-driven pathway.
(PDF)

S4 Fig (A-I) Additional model calibration data of hypoxia-driven pathway.
(PDF)

S5 Fig (A-J) Additional *in silico* investigation of pathway feedback within M1-M2 network.
(PDF)

S6 Fig (A-E) Temporal response of M2 markers and transcription factors under hypoxia.
(PDF)

S7 Fig (A-E) Parameter sensitivities under high IL-4 production.
(PDF)

S8 Fig Parameter distribution after bootstrapping. (PDF)

S1 Table Complete list of model reactions and parameter values. (PDF)

S2 Table Differential equations and initial conditions of all model nodes. (PDF)

S3 Table Summary of literature sources used in model calibration. (PDF)

S1 Protocol Additional information regarding model formulation and analysis. (PDF)

S1 Model Code Model code in SBML format (submitted as a separate .xml file). (XML)
