# Supplementary material for: A mechanistic integrative computational model of macrophage polarization: Implications in human pathophysiology
Source: PLoS Comput Biol. 2019 Nov 18;15(11):e1007468. doi: 10.1371/journal.pcbi.1007468 (PMC6860420; doi:10.1371/journal.pcbi.1007468)
Supplement: S1 File — (PDF) [file pcbi.1007468.s001.pdf]

Supporting Information File S1

A mechanistic integrative computational model of macrophage polarization: implications in human pathophysiology

Chen Zhao, Adam C. Mirando, Richard J. Sové, Thalyta X. Medeiros, Brian H. Annex, Aleksander S. Popel

Figure S1: Complete model diagram with all nodes and reactions

Figure S2: Additional model calibration data of IFN-γ-driven pathway

Figure S3: Additional model calibration data of IL-4-driven pathway

Figure S4: Additional model calibration data of hypoxia-driven pathway

Figure S5: Additional *in silico* investigation of pathway feedback within M1-M2 network

Figure S6: Temporal response of M2 markers and transcription factors under hypoxia

Figure S7: Parameter sensitivities under high IL-4 production

Figure S8: Parameter distribution after bootstrapping

Table S1: Complete list of model reactions and parameter values

Table S2: Differential equations and initial conditions of all model nodes

Table S3: Summary of literature sources used in model calibration

Protocol S1: Additional information regarding model formulation and analysis

Figure S1

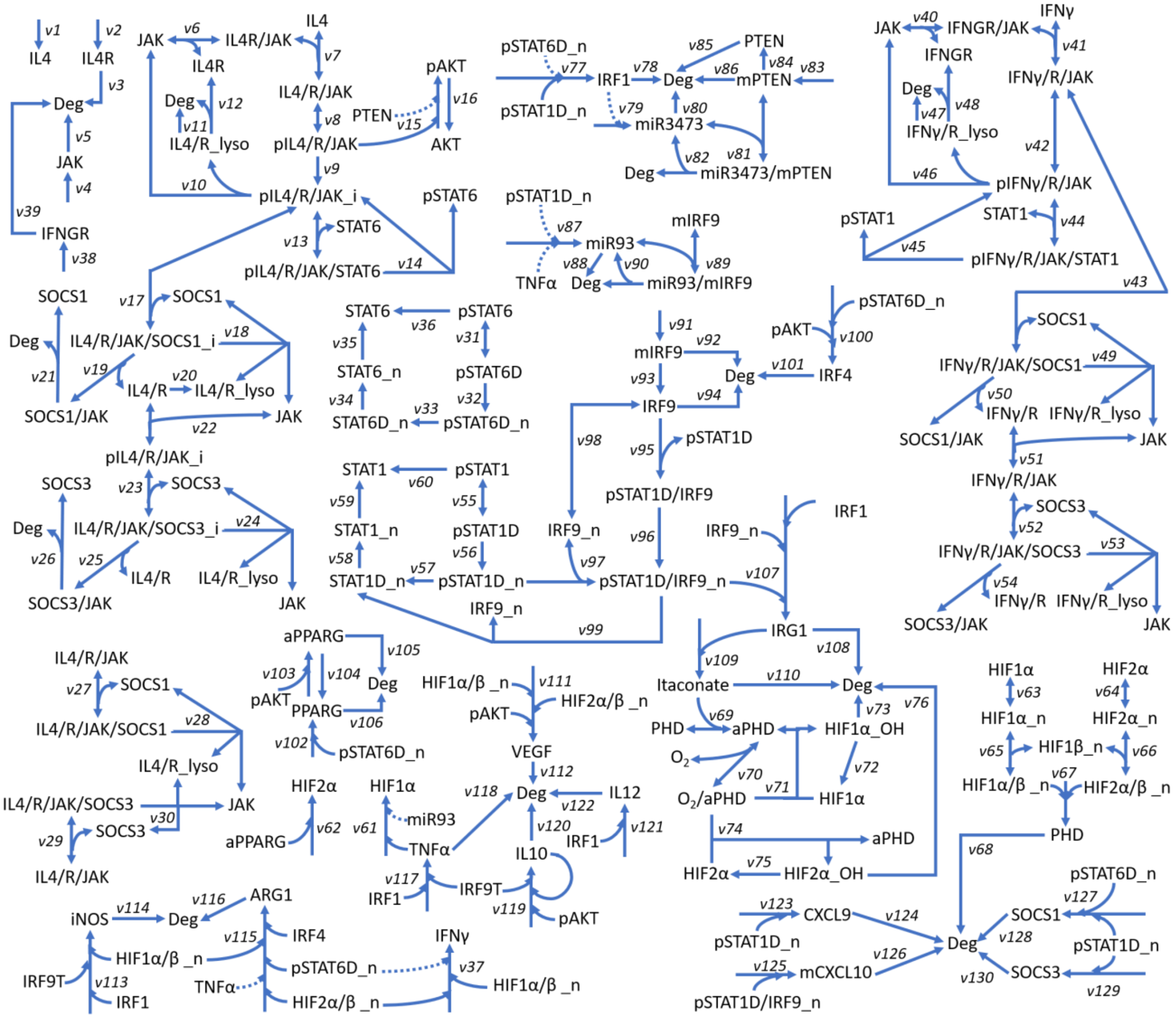

Figure S1. Complete model diagram with all nodes and reactions. Reaction rates are labeled (*v#*) and correspond to the rate laws described in detail in Table S1. For the names of model nodes (reaction species): 'X/Y' – complex formed by X and Y, 'pX' – phosphorylated form of species X, 'X_n' – species X in nucleus, 'mX' – mRNA of species X, 'aX' – species X in its activated form, 'Deg' – degradation, dashed arrows – inhibition. Reaction descriptions, parameters, rates and differential equations for all model nodes are summarized in Tables S1 and S2.

Figure S2

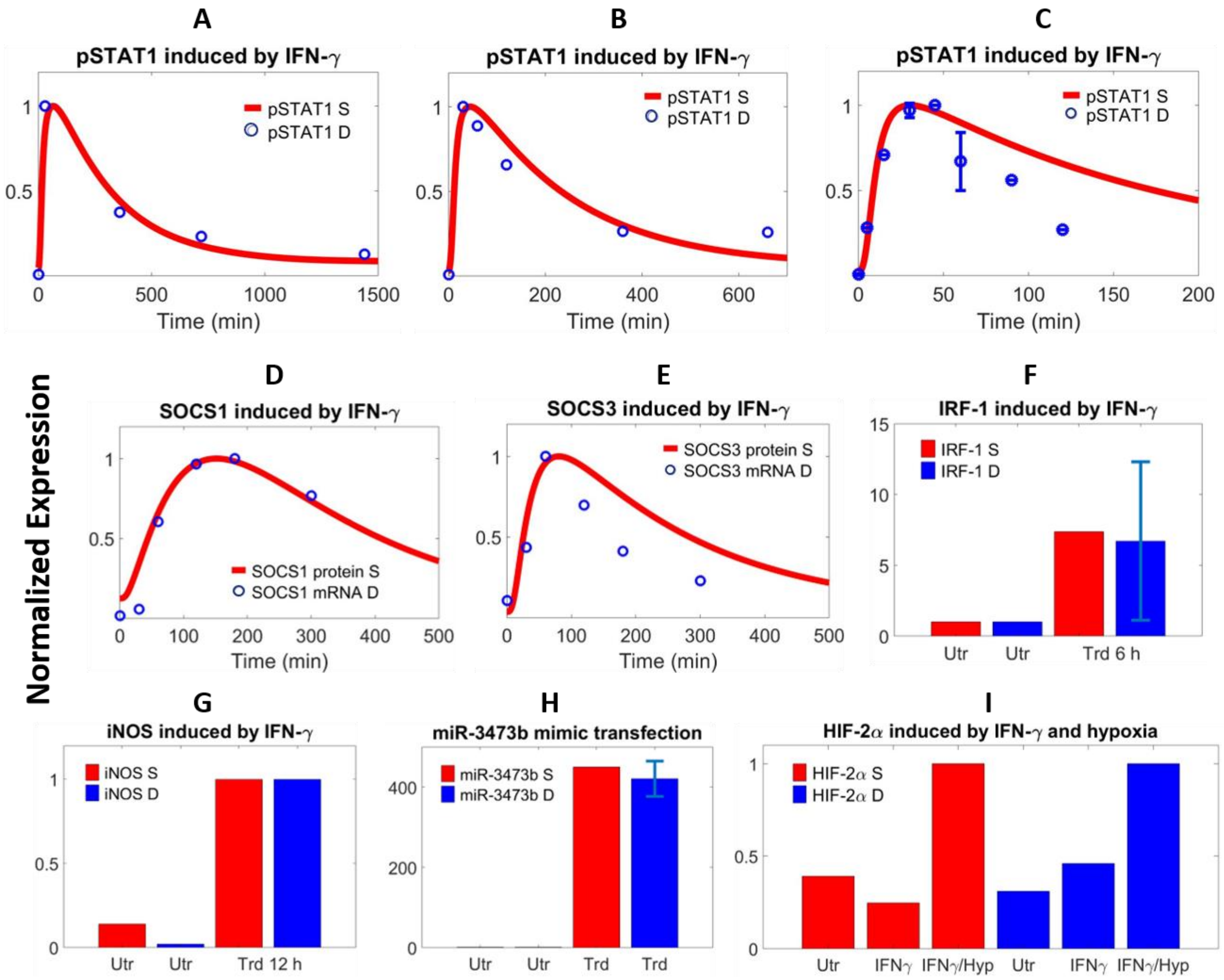

Figure S2. Additional model calibration data of IFN-γ-driven pathway. Comparisons between model simulations and literature experimental data of IFN-γ-induced (A-C) STAT1 activation at 5 ng/ml (1), 10 ng/ml (2), and 20 ng/ml of IFN-γ (3, 4), (D-E) upregulation of SOCS1 and SOCS3 (data is mRNA expression) (5), (F) increase in IRF-1 expression at 6 h (6), and (G) increase in iNOS expression at 12 h (7). (H) Data and simulated expression level of miR-3473b at 24 h after mimic transfection (8). (I) Hypoxia in combination with IFN-γ can significantly induce HIF-2α expression (for IFN-γ alone, simulation suggested a mild decrease while data suggested an insignificant increase in HIF-2α) (9). (A-I) All experimental data are measured in macrophage cell lines and values are for protein levels unless noted otherwise. Y-axes show normalized expression respectively (A-E: simulations and data are normalized to the maximum expression; F, H: normalized to the no-treatment/time 0 expression; G: normalized to the expression at 12 h; I: normalized to the expression under IFN-γ treatment with hypoxia). (D-E) For induction of SOCS1/3, data in terms of SOCS1/3 mRNA expression are compared with simulation (SOCS1/3 protein level), given that SOCS proteins are highly labile (direct protein measurements are scarce) and that Wormald et al. reported a tight temporal correlation between signaling-induced expression of SOCS1/3 protein and mRNA (10). S – simulation, D – literature data, Utr – untreated, Trd – IFN-γ treated, Hyp – hypoxia.

Figure S3

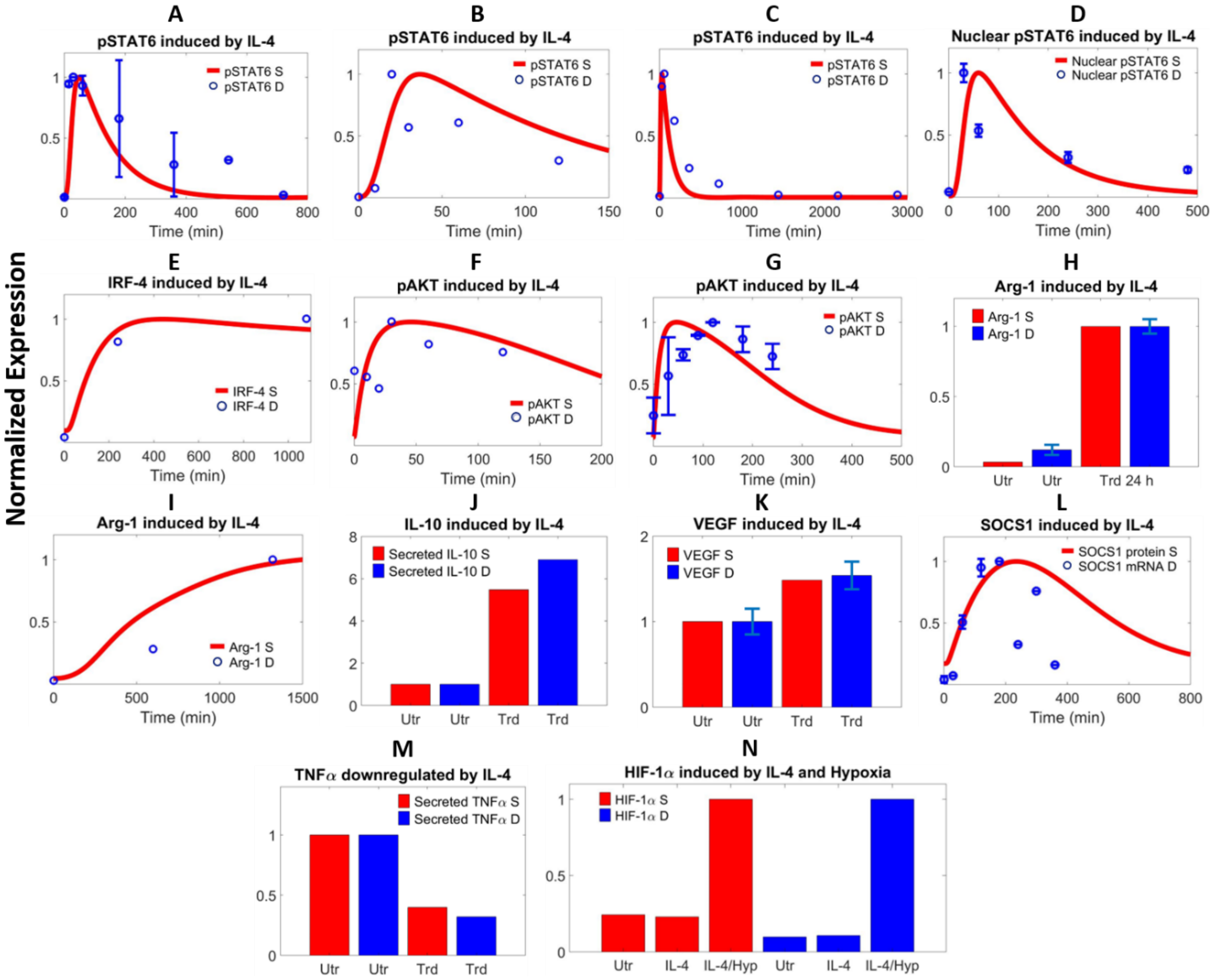

Figure S3. Additional model calibration data of IL-4-driven pathway. Comparisons between model simulations and published experimental data of IL-4-induced (A-C) STAT6 activation at 10 ng/ml (5, 11, 12), 50 ng/ml (13) and 100 nM IL-4 (14), (D) phosphorylated STAT6 in nucleus (15), (E) increase in IRF-4 expression (16), (F-G) AKT activation at 50 ng/ml (17) and 20 ng/ml IL-4 (18), (H-I) increase in Arg-1 production (15, 19), (J) increase in IL-10 production at 24 h (20), (K) increase in VEGF production (21), (L) SOCS1 induction (5), and (M) inhibition of TNFα production at 24 h (20). (N) Simulation and data show that hypoxia can stabilize HIF-1α protein while IL-4 stimulation has no effect (9). (A-N) All experimental data are measured in macrophage cell lines and values are for protein levels unless noted otherwise. Y-axes show normalized expression respectively (A-G, I, L: simulations and data are normalized to the maximum expression; J, K, M: normalized to the no-treatment/time 0 expression; H: normalized to the expression at 24 h; N: normalized to the expression under IL-4 treatment with hypoxia). (H) For Arg-1 production, data in terms of Arg-1 activity (formation of urea from arginine) is compared with simulation (Arg-1 protein level). (K) For VEGF production, data in terms of intracellular VEGF level is compared with simulation (secreted VEGF level). (L) For SOCS1 induction, data in terms of SOCS1 mRNA level is compared with simulation (SOCS1 protein level), given the reasoning stated in Figures S2D-E. S – simulation, D – literature data, Utr – untreated, Trd – IL-4 treated, Hyp – hypoxia.

Figure S4

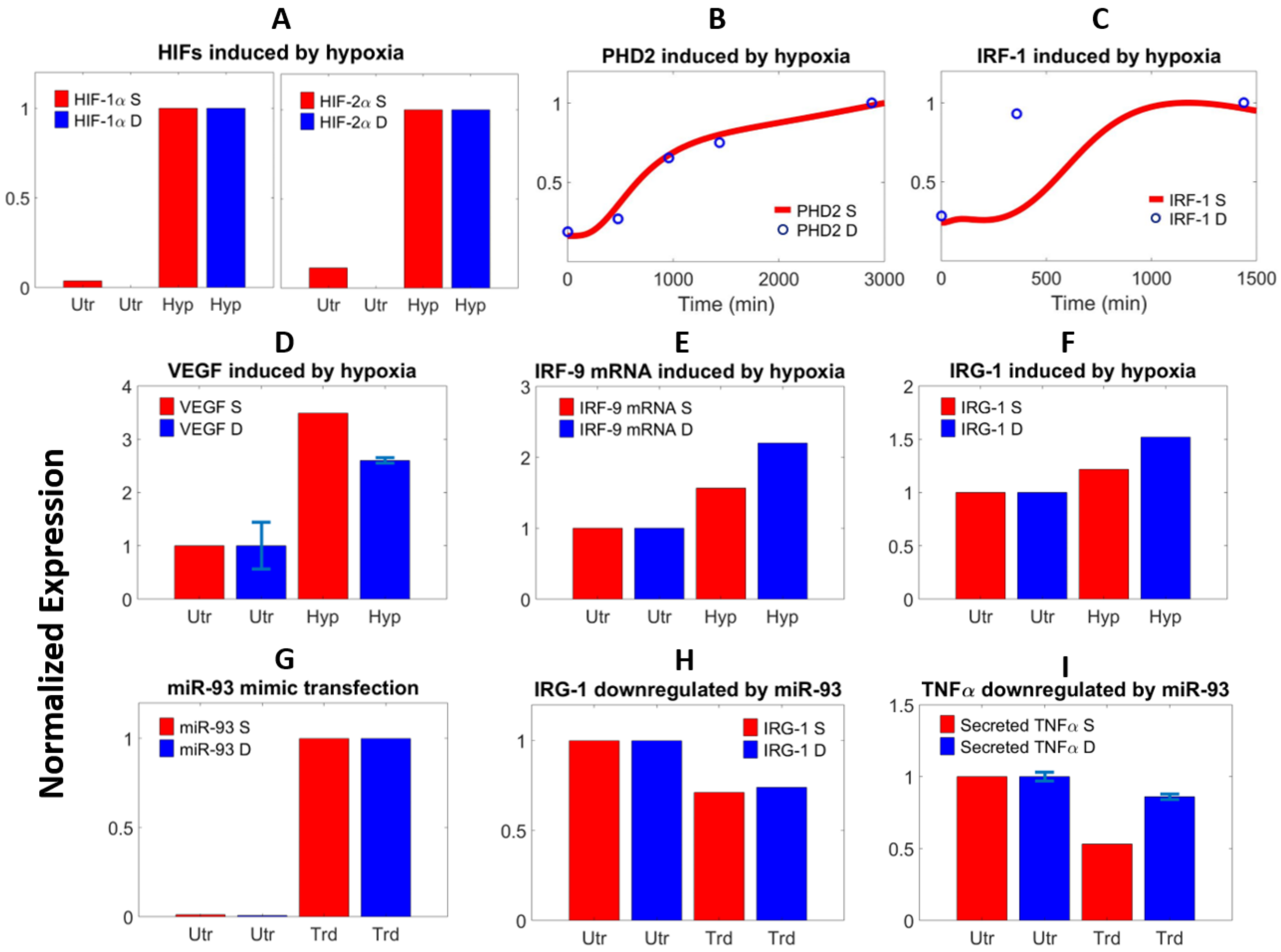

Figure S4. Additional model calibration data of hypoxia-driven pathway. Comparisons between model simulations and literature experimental data of hypoxia-induced (A) stabilization of HIF-1α and HIF-2α at 18 h (22), (B) PHD upregulation (23), (C) IRF-1 induction (24), (D) VEGF production at 24 h (25), (E) de-suppression of IRF-9 (26) and (F) downstream upregulation of IRG-1 at 12 h (26). (G) Data and simulated expression level of miR-93 before and after 24 h of mimic transfection, which leads to downregulation of (H) IRG-1 abundance at 24 h (26). (I) Transfection of miR-93 mimic decreases TNFα production at 12 h under hypoxia (26). (A-I) All experimental data are measured in macrophage cell lines (except for B, which is in Hela cells) and values are for protein levels unless noted otherwise. Y-axes show normalized expression respectively (A: simulation and data are normalized to the expression under hypoxia; B, C: normalized to the maximum expression; D, E, F, H: normalized to the no-treatment expression; G: normalized to the miR-93 expression at 24 h after transfection; I: normalized to the expression under 12 h of hypoxia without miR-93 mimic). (D) For VEGF production, data in terms of intracellular VEGF level is compared with simulation (secreted VEGF level). (F and H) For IRG-1 regulation, data in terms of IRG-1 mRNA level is compared with simulation (IRG-1 protein level). S – simulation, D – literature data, Utr – untreated, Trd – treated with miR-93 mimic, Hyp – hypoxia.

Figure S5

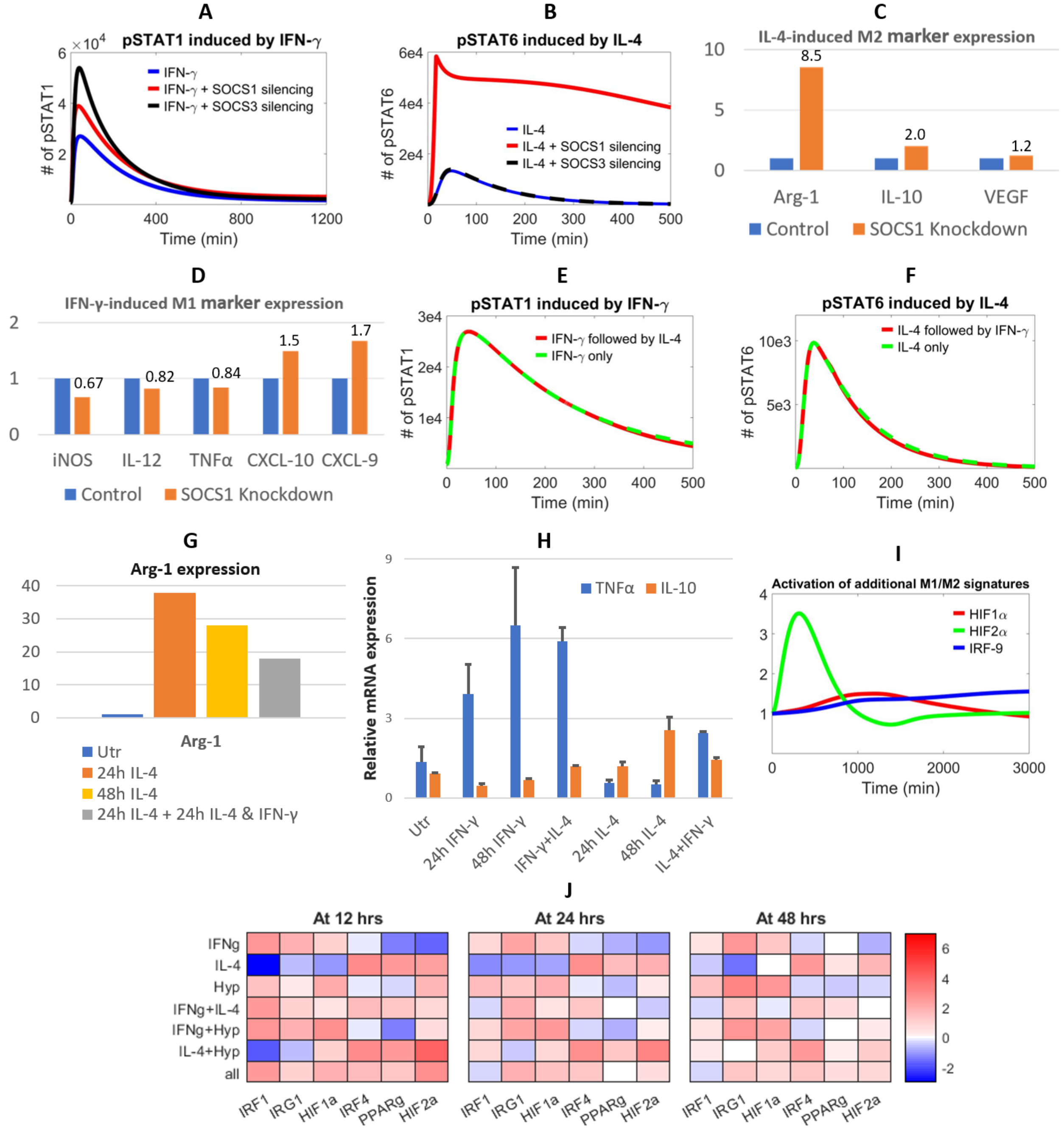

Figure S5. Additional *in silico* investigation of pathway feedback within M1-M2 network. (A) Silencing of SOCS1 or SOCS3 in macrophages can promote activation of STAT1 by IFN-γ. (B) SOCS1 silencing, but not SOCS3 silencing, can markedly boost STAT6 activation by IL-4. (A-B) Silencing is modeled as 0x initial level with 0x production. Knockdown of SOCS1 (modeled as 0.3x initial level with 0.3x production) (C) promotes IL-4-induced M2 marker expression and (D) differentially influences M1 marker expression in response to IFN-γ. In the scenarios of IFN-γ stimulation followed by the addition of IL-4 (at 4 h), or IL-4 stimulation followed by the addition of IFN-γ (at 1 hr), there is no obvious change of (E) STAT1 or (F) STAT6 activation. (G) The addition of a second stimulus IFN-γ (at 24 h post IL-4 stimulation) can antagonize the expression pattern of Arg-1 induced by IL-4. (H) RT-qPCR analysis

(results presented as mean ± SEM, n=3) of TNFα and IL-10 gene expression in THP-1 cells stimulated with 24 and 48 h of IFN-γ (or IL-4), and 24 h of IFN-γ (or IL-4) then another 24 h of IFN-γ plus IL-4 (labeled as IFNγ+IL-4 or IL-4+IFNγ). (I) Temporal expression profiles of HIFs and IRF-9 when macrophages are stimulated with IFN-γ and IL-4 simultaneously. (J) Temporal relative protein expression patterns of six M1-M2 signature transcription factors in macrophages under seven different stimulation conditions (‘A+B’ means simultaneous stimulation, ‘all’ means IFN-γ+IL-4+hypoxia, all expression levels are normalized to the untreated/time 0 levels and then log2 transformed). (A-J) All simulation results are protein levels (except CXCL10 is mRNA level). (C,D,G,I) Y-axes show relative expression (normalized to untreated/control/time 0 levels). Simulated treatment doses are 10 ng/ml IFN-γ and 10 ng/ml IL-4 for (A-D), 10 ng/ml IFN-γ and 20 ng/ml IL-4 for (E-F), 20 ng/ml IFN-γ and 20 ng/ml IL-4 for (G), 10 ng/ml IFN-γ and 5 ng/ml IL-4 for (I-J). Utr – untreated, Hyp – hypoxia (2% oxygen for J).

Figure S6

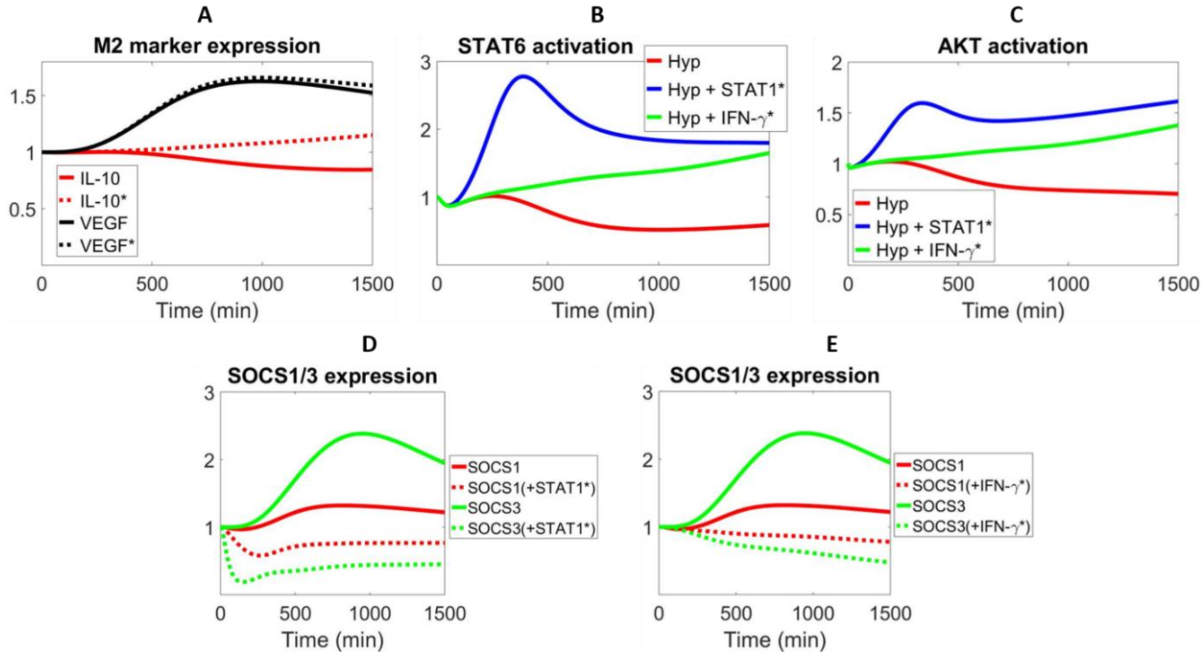

Figure S6. Temporal response of M2 markers and transcription factors under hypoxia. (A) Inhibition of IFN-γ production under hypoxia (dashed lines) can upregulation the expression of M2 markers IL-10 and VEGF (marginal effect), compared to hypoxia alone (solid lines). Simulations show stronger temporal activation of (B) pSTAT6 and (C) pAKT under hypoxia in combination with IFN-γ inhibition or STAT1 inhibition, compared to hypoxia alone. SOCS1 and SOCS3 expression are reduced under hypoxia with either (D) STAT1 or (E) IFN-γ inhibition (dashed lines), compared to hypoxia alone (solid lines). STAT1* means inhibition of STAT1 activation, IFN-γ* means inhibition of IFN-γ production. Inhibition of IFN-γ is simulated by setting its production rate to 10% of the original value; STAT1 inhibition is simulated as a 90% decrease in the binding rate between STAT1 and activated IFN-γ receptor complex. (A-E) Expression levels are normalized to their respective t=0 values (e.g. normoxia, unstimulated). Hyp – hypoxia. All simulation results are protein levels.

Figure S7

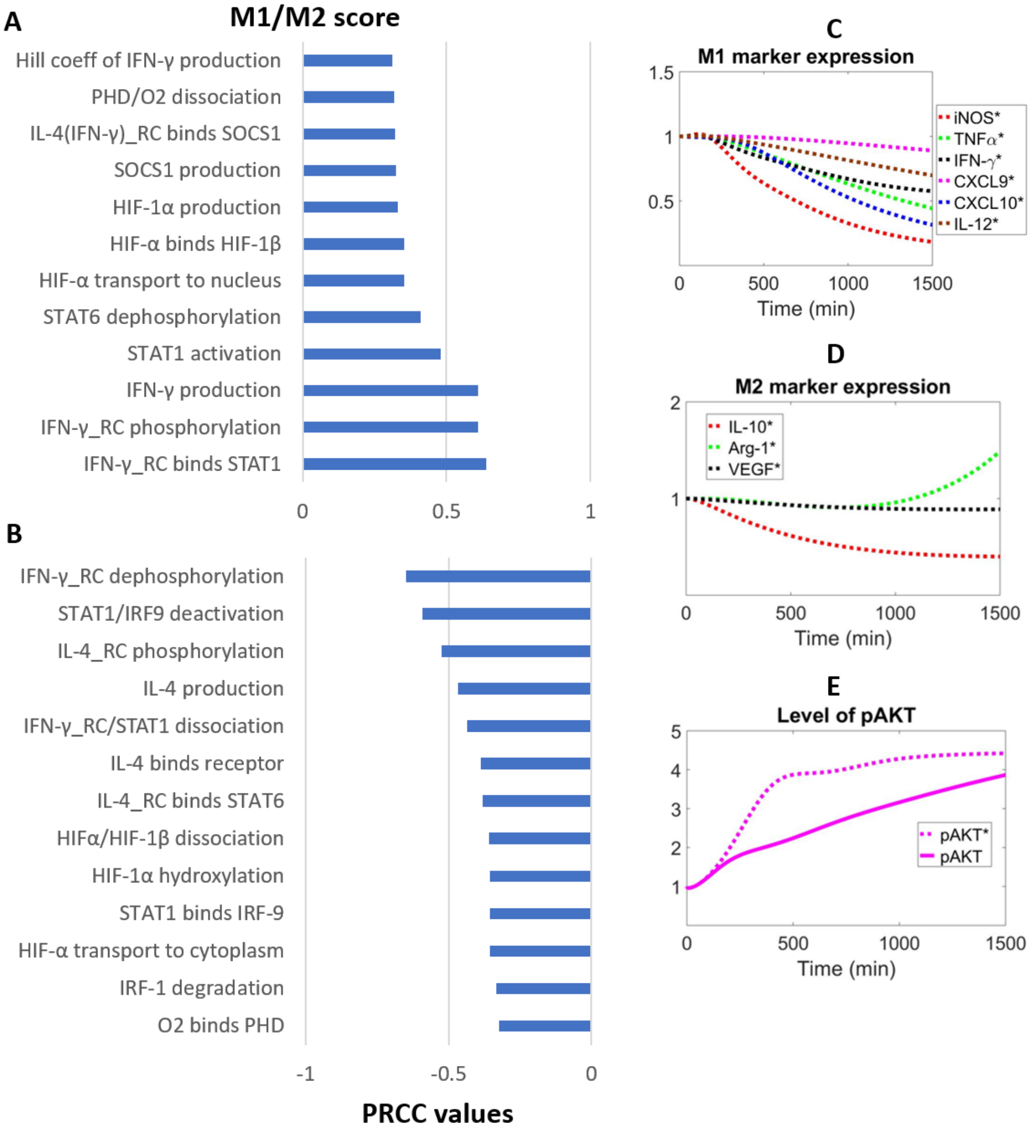

Figure S7. Parameter sensitivities under high IL-4 production. (A-B) Sensitivity indices (top 25 positive and negative PRCC values with $p<0.05$) of model parameters that control M1 and M2 marker expression in terms of the M1/M2 score under high IL-4 production (10x). In the parameter descriptions, 'X_RC' means receptor complex formed by ligand X, receptor and JAK, 'X/Y' means complex formed by X and Y. (C-D) Simulated relative time-course expression (dashed lines) of M1 and M2 markers when macrophages are subjected to AKT inhibition (simulated as a 90% decrease in the AKT activation rate) under high IL-4 production. (E) Under the scenario of high IL-4 production, inhibition of STAT6 triggers increased activation of AKT as a compensatory mechanism to further upregulate M2 marker expression. (C-E) Marker expression levels are normalized to their respective t=0 values (e.g. normal IL-4 production, unstimulated). All simulation results are protein levels (except CXCL10 is mRNA level). (A-B) More details about the parameters listed can be found in Table S1 using the labels (positive – *ka37, kr70, kf17, k127, k61, kf64, kf63, k33, k45, k37, kf42, kf44*; negative – *kr42, k99, kf8, k1, kr44, kf7, kf13, kr64, k71, kf95, kr63, k78, kf70*; order is from top to bottom as displayed).

Figure S8

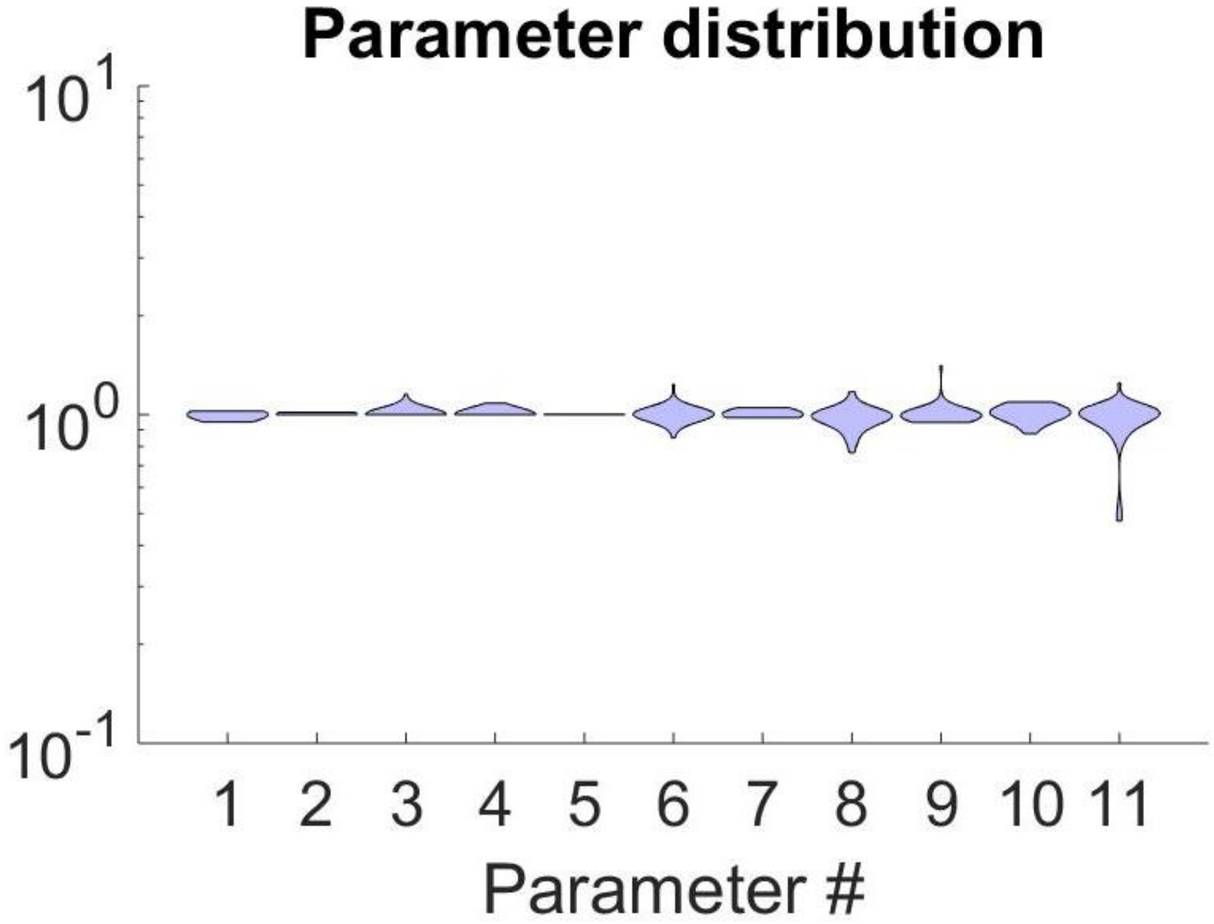

Figure S8. Parameter distribution after bootstrapping. Parameter estimate distributions (represented by violin plots) of the top 11 most sensitive parameters. The 11 parameters were re-estimated 50 times following the resampling procedures described in the Materials and Methods section (all parameter values are normalized to their respective original values for display, y-axis in log scale). During bootstrapping, parameter values are allowed to vary from 0.1x to 10x (of their original values). Parameter descriptions – #1. IFN-γ receptor dephosphorylation rate; 2. forward binding rate of activated IFN-γ receptor complex with STAT1; 3. deactivation rate of STAT1/IRF9 complex in nucleus; 4. IFN-γ receptor phosphorylation rate; 5. reverse binding (e.g. dissociation) rate of activated IFN-γ receptor complex with STAT1; 6. forward binding rate of IL-4 (or IFN-γ) receptor complex with SOCS1; 7. dephosphorylation rate of STAT6; 8. degradation rate of SOCS1/3-bound IL-4 (or IFN-γ) receptor complex; 9. rate of IL-4 receptor phosphorylation; 10. STAT1 activation rate; 11. forward binding rate of IFN-γ receptor complex with SOCS3. The 11 parameters are chosen based on the overall ranking of their absolute PRCC values (from high to low) derived from the sensitivity analysis in three scenarios (IL-4 stimulation, IFN-γ stimulation, and hypoxia).

Table S1

No.	Reaction descriptions	Reaction flux details (v=)	Parameter values	Refs.
v1	IL-4 production	*k1*	*k1*=0.2 min^{-1}	Fitted
v2	IL-4R production	*k2*	*k2*=0.16 min^{-1}	Fitted
v3	IL-4R constitutive degradation	*k3**[IL4R]	*k3*=0.007 min^{-1}	Fitted
v4	JAK production	*k4*	*k4*=50 min^{-1}	Fitted
v5	JAK constitutive degradation	*k5**[JAK]	*k5*=0.0005 min^{-1}	(27)
v6	IL-4R pre-associates with JAK	*kf6**[JAK]*[IL4R]-*kr6**[IL4R/JAK]	*kf6*=4e-6 min^{-1}, *kr6*=0.0018 min^{-1}	Fitted
v7	IL-4 binds receptor	*kf7**[IL4]*[IL4R/JAK]-*kr7**[IL4/R/JAK]	*kf7*=2e-7 min^{-1}, *kr7*=0.01 min^{-1}	(28)
v8	IL-4 receptor complex phosphorylation	*kf8**[IL4/R/JAK]-*kr8**[pIL4/R/JAK]	*kf8*=0.6 min^{-1}, *kr8*=0.1 min^{-1}	Fitted
v9	Internalization of phosphorylated IL-4 receptor complex	*k9**[pIL4/R/JAK]	*k9*=0.2 min^{-1}	Fitted
v10	Shuttling of IL-4 receptor complex to lysosomes	*k10**[pIL4/R/JAK_i]	*k10*=1 min^{-1}	Fitted
v11	Degradation of IL-4 and IL-4R in lysosomes	*k11**[IL4/R_lyso]	*k11*=0.3 min^{-1}	Fitted
v12	Recycling of IL-4R	*k12**[IL4/R_lyso]	*k12*=1 min^{-1}	Fitted
v13	Binding of STAT6 with ligand-activated IL-4 receptor complex	*kf13**[STAT6]*[pIL4/R/JAK_i]-*kr13**[pIL4/R/JAK/STAT6]	*kf13*=0.1 min^{-1}, *kr13*=10 min^{-1}	Fitted
v14	Activation of STAT6 by phosphorylation	*k14**[pIL4/R/JAK/STAT6]	*k14*=8 min^{-1}	Fitted
v15	Phosphorylation of AKT	*k15**[AKT]*(1-[PTEN]/([PTEN]+ka15))*([pIL4/R/JAK]/([pIL4/R/JAK]+kb15))	*k15*=1.16 min^{-1}, *ka15*=5000, *kb15*=4	Fitted
v16	AKT dephosphorylation	*k16**[pAKT]	*k16*=0.015 min^{-1}	(29)
v17	Internalized IL-4 receptor complex binds SOCS1	*kf17**[pIL4/R/JAK_i]*[SOCS1]-*kr17**[IL4/R/JAK/SOCS1_i]	*kf17*=0.008 min^{-1}, *kr17*=0.2 min^{-1}	Fitted
v18	SOCS1 inhibits IL-4 signaling and shuttles IL-4 and receptor to lysosomes	*k10**[IL4/R/JAK/SOCS1_i]		*
v19	SOCS1 sequesters JAK from the IL-4 receptor complex	*k19**[IL4/R/JAK/SOCS1_i]	*k19*=0.03 min^{-1}	Fitted
v20	Shuttling of internalized IL-4 and receptor to lysosomes	*k10**[IL4/R]		*
v21	SOCS1 targets JAK for degradation	*k21**[SOCS1/JAK]	*k21*=0.1 min^{-1}	Fitted
v22	Internalized IL-4 and receptor associate with JAK to reactivate signaling	*kf6**[IL4/R]*[JAK]-*kr6**[pIL4/R/JAK_i]		*
v23	Internalized IL-4 receptor complex binds SOCS3	*kf23**[pIL4/R/JAK_i]*[SOCS3]-*kr17**[IL4/R/JAK/SOCS3_i],	*kf23*=0.0004 min^{-1}	Fitted *

v24	SOCS3 inhibits IL-4 signaling and shuttles IL-4 and receptor to lysosomes	*k10**[IL4/R/JAK/SOCS3_i]		*
v25	SOCS3 sequesters JAK from the IL-4 receptor complex	*k19**[IL4/R/JAK/SOCS3_i]		*
v26	SOCS3 targets JAK for degradation	*k26**[SOCS3/JAK]	*k26*=0.01 min^{-1}	Fitted
v27	Surface IL-4 receptor complex binds SOCS1	*kf17**[IL4/R/JAK]*[SOCS1]-*kr17**[IL4/R/JAK/SOCS1]		*
v28	SOCS1-mediated shuttling of surface IL-4 and receptor to lysosomes	*k28**[IL4/R/JAK/SOCS1]	*k28*=0.1 min^{-1}	Fitted
v29	Surface IL-4 receptor complex binds SOCS3	*kf23**[IL4/R/JAK]*[SOCS3]-*kr17**[IL4/R/JAK/SOCS3]		*
v30	SOCS3-mediated shuttling of surface IL-4 and receptor to lysosomes	*k28**[IL4/R/JAK/SOCS3]		*
v31	pSTAT6 dimerization	*kf31**[pSTAT6]*[pSTAT6]-*kr31**[pSTAT6D]	*kf31*=0.002 min^{-1}, *kr31*=1 min^{-1}	Fitted
v32	Activated STAT6 dimer transports to nucleus	*k32**[pSTAT6D]	*k32*=0.4 min^{-1}	Fitted
v33	Dephosphorylation of nuclear STAT6 dimer	*k33**[pSTAT6D_n]	*k33*=0.01 min^{-1}	(30)
v34	Dissociation of STAT6 dimer in nucleus	*kr31**[STAT6D_n]		*
v35	Nuclear export of STAT6	*k35**[STAT6_n]	*k35*=0.05 min^{-1}	(31)
v36	Dephosphorylation of STAT6	*k33**[pSTAT6]		*
v37	IFN-γ production	*k37**([HIF1α/β_n]+0.2*[HIF2α/β_n])*(1-[pSTAT6D_n]/([pSTAT6D_n]+*ka37*))	*k37*=0.0288 min^{-1}, *ka37*=100	Fitted
v38	IFNGR production	*k38*	*k38*=1.22 min^{-1}	Fitted
v39	IFNGR constitutive degradation	*k39**[IFNGR]	*k39*=0.006 min^{-1}	(32)
v40	IFNGR pre-associates with JAK	*kf6**[IFNGR]*[JAK]-*kr40**[IFNGR/JAK]	*kr40*=0.002 min^{-1}	Fitted *
v41	IFN-γ binds receptor	*kf41**[IFNγ]*[IFNGR/JAK]-*kr7**[IFNγ/R/JAK]	*kf41*=1.6e-7 min^{-1}	(33, 34)*
v42	IFN-γ receptor complex phosphorylation	*kf42**[IFNγ/R/JAK]-kr42*[pIFNγ/R/JAK]	*kf42*=3 min^{-1}, k*r42*=10 min^{-1}	Fitted
v43	IFN-γ receptor complex binds SOCS1	*kf17**[IFNγ/R/JAK]*[SOCS1]-*kr17**[IFNγ/R/JAK/SOCS1]		*
v44	STAT1 binds ligand-activated IFN-γ receptor complex	*kf44**[STAT1]*[pIFNγ/R/JAK]-*kr44**[pIFNγ/R/JAK/STAT1]	*kf44*=0.03 min^{-1}, *kr44*=3 min^{-1}	Fitted
v45	STAT1 activation by phosphorylation	*k45**[pIFNγ/R/JAK/STAT1]	*k45*=2 min^{-1}	Fitted
v46	Shuttling of IFN-γ receptor complex to lysosomes	*k46**[pIFNγ/R/JAK]	*k46*=0.1 min^{-1}	Fitted

v47	Degradation of IFN-γ and IFNGR in lysosomes	*k47**[IFNγ/R_lyso]	*k47*=1 min^{-1}	Fitted
v48	Recycling of IFNGR	*k12**[IFNγ/R_lyso]		*
v49	SOCS1 inhibits IFN-γ signaling and shuttles IFN-γ and receptor to lysosomes	*k46**[IFNγ/R/JAK/SOCS1]		*
v50	SOCS1 sequesters JAK from the IFN-γ receptor complex	*k19**[IFNγ/R/JAK/SOCS1]		*
v51	IFN-γ and receptor bind JAK to reactivate signaling	*kf6**[IFNγ/R]*[JAK]-*kr40**[IFNγ/R/JAK],		*
v52	IFN-γ receptor complex binds SOCS3	*kf52**[IFNγ/R/JAK]*[SOCS3]-*kr17**[IFNγ/R/JAK/SOCS3]	*kf52*=0.004 min^{-1}	Fitted *
v53	SOCS3 inhibits IFN-γ signaling and shuttles IFN-γ and receptor to lysosomes	*k46**[IFNγ/R/JAK/SOCS3]		*
v54	SOCS3 sequesters JAK from the IFN-γ receptor complex	*k19**[IFNγ/R/JAK/SOCS3]		*
v55	pSTAT1 dimerization	*kf55**[pSTAT1]*[pSTAT1]-*kr31**[pSTAT1D]	*kf55*=0.1 min^{-1}	Fitted *
v56	Activated STAT1 dimer transports to nucleus	*k56**[pSTAT1D]	*k56*=1 min^{-1}	Fitted
v57	Dephosphorylation of nuclear STAT1 dimer	*k57**[pSTAT1D_n]	*k57*=0.03 min^{-1}	(35)
v58	Dissociation of STAT1 dimer in nucleus	*kr31**[STAT1D_n]		*
v59	Nuclear export of STAT1	*k59**[STAT1_n]	*k59*=0.1 min^{-1}	Fitted
v60	Dephosphorylation of STAT1	*k57**[pSTAT1]		*
v61	HIF-1α production is promoted by TNFα signaling and downregulated by miR-93	*k61**([TNFα]+*ka61*)*(1.4-[miR93]/([miR93]+*kb61*))	*k61*=0.0187 min^{-1}, *ka61*=800, *kb61*=1000	Fitted
v62	HIF-2α production is promoted by PPARγ	*k62**([aPPARG]/([aPPARG]+*ka62*))	*k62*=178 min^{-1}, *ka62*=10000	Fitted
v63	HIF-1α transport to nucleus	*kf63**[HIF1α]-*kr63**[HIF1α_n]	*kf63*=0.005 min^{-1}, *kr63*=0.018 min^{-1}	(36)
v64	HIF-2α transport to nucleus	*kf63**[HIF2α]-*kr63**[HIF2α_n]		*
v65	Nuclear HIF-1α binds HIF-1β	*kf64**[HIF1α_n]*[HIF1β_n]-*kr64**[HIF1α/β_n]	*kf64*=5e-7 min^{-1}, *kr64*=0.03 min^{-1}	Fitted; (36)
v66	Nuclear HIF-2α binds HIF-1β	*kf64**[HIF2α_n]*[HIF1β_n]-*kr64**[HIF2α/β_n]		*
v67	PHD production	*k67**(0.0001+[HIF1α/β_n]^2/([HIF1α/β_n]^2+*ka67*)*[HIF2α/β_n]^2/([HIF2α/β_n]^2+*kb67*))	*k67*=602 min^{-1}, *ka67*=32600, *kb67*=25400	Fitted
v68	PHD degradation	*k68**[PHD]	*k68*=0.0008 min^{-1}	(27)
v69	Itaconate influences PHD activation	*kf69**[PHD]*[Itaconate]-*kr69**[aPHD]	*kf69*=8e-9 min^{-1}, *kr69*=0.07 min^{-1}	Fitted
v70	PHD binds oxygen	*kf70**[aPHD]*[O_2]-*kr70**[O_2/aPHD]	*kf70*=7.14e-9 min^{-1}, *kr70*=10.8 min^{-1}	Fitted; (36)
v71	HIF1α hydroxylation	*k71**[O_2/aPHD]*[HIF-1α]	*k71*=1.33e-5 min^{-1}	Fitted

v72	HIF1α de-ubiquitination	*k72**[HIF1α_OH]	*k72*=0.03 min^{-1}	Fitted
v73	Degradation of hydroxylated HIF-1α	*k73**[HIF1α_OH]	*k73*=0.6 min^{-1}	Fitted
v74	HIF2α hydroxylation	*k74**[O_2/aPHD]*[HIF-2α]	*k74*=5e-6 min^{-1}	Fitted
v75	HIF2α de-ubiquitination	*k72**[HIF2α_OH]		*
v76	Degradation of hydroxylated HIF-2α	*k73**[HIF2α_OH]		*
v77	STAT1 and STAT6 regulate IRF-1 production	*k77**(([pSTAT1D_n]+[pSTAT1D/IRF9_n])/[pSTAT6D_n])/(([pSTAT1D_n]+[pSTAT1D/IRF9_n])/[pSTAT6D_n]+*ka77*)	*k77*=106 min^{-1}, *ka77*=50	Fitted
v78	IRF-1 degradation	*k78**[IRF1]	*k78*=0.022 min^{-1}	(37)
v79	IFN-γ (through IRF-1) inhibits miR-3473 production	*k79**(1-$[IRF1]^2$/($[IRF1]^2$+*ka79*))	*k79*=14.8 min^{-1}, *ka79*=40000	Fitted
v80	Degradation of miR-3473	*k80**[miR3473]	*k80*=0.0012 min^{-1}	(38)
v81	miR-3473 binds PTEN mRNA	*kf81**[miR3473]*[mPTEN]-*kr81**[miR3473/mPTEN]	*kf81*=0.0001 min^{-1}, *kr81*=0.06 min^{-1}	(39)
v82	Degradation of miR3473-bound PTEN mRNA	*k82**[miR3473/mPTEN]	*k82*=0.1 min^{-1}	Fitted
v83	Production of PTEN mRNA	*k83*	*k83*=0.64 min^{-1}	Fitted
v84	PTEN translation	*k84**[mPTEN]	*k84*=2.32 min^{-1}	Fitted
v85	PTEN degradation	*k85**[PTEN]	*k85*=0.0003 min^{-1}	(27)
v86	PTEN mRNA constitutive degradation	*k86**[mPTEN]	*k86*=0.003 min^{-1}	(40)
v87	miR-93 production is downregulated by IFN-γ (represented by STAT1) and TNFα signaling	*k87**(1-[TNFα]*$[pSTAT1D\_n]^2$/([TNFα] *$[pSTAT1D\_n]^2$+*ka87*))	*k87*=6050 min^{-1}, *ka87*=1000	Fitted
v88	miR-93 degradation	*k88**[miR93]	*k88*=0.0018 min^{-1}	(38)
v89	miR-93 binds IRF9 mRNA	*kf81**[miR93]*[mIRF9]-*kr*81*[miR93/mIRF9]		*
v90	Degradation of miR93-bound IRF9 mRNA	*k82**[miR93/mIRF9]		*
v91	IRF9 mRNA production	*k91*	*k91*=0.5 min^{-1}	Fitted
v92	IRF9 mRNA constitutive degradation	*k86**[mIRF9]		*
v93	IRF9 translation	*k93**[mIRF9]	*k93*=1.18 min^{-1}	Fitted
v94	IRF9 degradation	*k94**[IRF9]	*k94*=0.004 min^{-1}	Fitted
v95	IRF9 binds activated STAT1 dimer in cytoplasm	*kf95**[IRF9]*[pSTAT1D]-*kr95**[pSTAT1D/IRF9]	*kf95*=0.00013 min^{-1}, *kr95*=1 min^{-1}	Fitted
v96	Nuclear translocation of STAT1/IRF9 complex	*k96**[pSTAT1D/IRF9]	*k96*=0.1 min^{-1}	Fitted
v97	IRF9 binds activated STAT1 dimer in nucleus	*kf95**[IRF9_n]*[pSTAT1D_n]-*kr95**[pSTAT1D/IRF9_n]		*
v98	IRF9 translocation to nucleus	*k96**[IRF9]-*kr98**[IRF9_n]	*kr98*=0.01 min^{-1}	Fitted
v99	Deactivation of STAT1/IRF9 complex in nucleus	*k99**[pSTAT1D/IRF9_n]	*k99*=0.4 min^{-1}	Fitted

v100	IRF4 production regulated by STAT6 and AKT	*k100**[pSTAT6D_n]/([pSTAT6D_n]+*ka100*)*[pAKT]/([pAKT]+*kb100*)	*k100*=6840 min^{-1}, *ka10*0=30000, *kb100*=20000	Fitted
v101	IRF4 degradation	*k101**[IRF4]	*k101*=0.0002 min^{-1}	(27)
v102	STAT6 promotes PPARγ production	*k102**[pSTAT6D_n]/([pSTAT6D_n]+*ka102*)	*k102*=107 min^{-1}, *ka102*=1000	Fitted
v103	AKT promotes PPARγ activation	*k103**[PPARG]*[pAKT]/([pAKT]+*ka103*)	*k103*=50 min^{-1}, *ka103*=10000	Fitted
v104	PPARγ deactivation	*k104**[aPPARG]	*k104*=50 min^{-1}	Fitted
v105	Degradation of activated PPARγ	*k105**[aPPARG]	*k105*=0.0018 min^{-1}	(41)
v106	Degradation of PPARγ	*k105**[PPARG]		*
v107	IRF9 and IRF1 promotes IRG-1 production	*k107**[IRF1]*([IRF9_n]+[pSTAT1D/IRF9_n])^2	*k107*=4.77e-12 min^{-1}	Fitted
v108	IRG1 degradation	*k108**[IRG1]	*k108*=6.2e-4 min^{-1}	(27)
v109	IRG1 promotes itaconate production	*k109**[IRG1]^2	*k109*=0.025 min^{-1}	Fitted
v110	Itaconate degradation	*k110**[Itaconate]	*k110*=0.005 min^{-1}	Fitted
v111	HIF1/2 and AKT induce VEGF production	*k111**([HIF1α/β_n]*[HIF2α/β_n]+*ka111*)*([pAKT]+*kb111*)	*k111*=3e-9 min^{-1}, *ka111*=29200, *kb111*=42700	Fitted
v112	Removal of secreted VEGF	*k112**[VEGF]	*k112*=0.001 min^{-1}	Fitted
v113	iNOS production is dependent on HIF1, IRF1 and type I IFN signaling (represented by IRF9)	*k113**(0.02+[HIF1α/β_n]/([HIF1α/β_n]+*ka113*))*([IRF1]/([IRF1]+*kb113*))*([IRF9T]+15000)	*k113*=0.036 min^{-1}, *ka113*=4000, *kb113*=9000	Fitted
v114	iNOS degradation	*k114**[iNOS]	*k114*=0.006 min^{-1}	(42)
v115	ARG1 production is promoted by STAT6, IRF4 and HIFs while downregulated by TNFα	*k115**([pSTAT6D_n]*[IRF4]+*ka115*)*([HIF1α/β_n]+2*[HIF2α/β_n])^2*(1.2-[TNFα]^2/([TNFα]^2+*kb115*))	*k115*=3.76e-9 min^{-1}, *ka115*=1.2e7, *kb115*=100000	Fitted
v116	ARG1 degradation	*k116**[ARG1]	*k116*=0.0006 min^{-1}	(43, 44)
v117	IRF1 and type I IFNs (represented by IRF9) can induce TNFα production	*k117** [IRF1]^2/([IRF1]^2+*ka117*)*(30000+[IRF9T])^2	*k117*=7.2e-10 min^{-1}, *ka117*=1.6e6	Fitted
v118	Removal of secreted TNFα	*k118**[TNFα]	*k118*=0.0008 min^{-1}	Fitted
v119	IL-10 production is promoted by AKT, type I IFNs (represented by IRF9) and STAT3 (represented by secreted IL-10)	*k119**[pAKT]*(2000+[IL10])*([IRF9T]+15000)	*k119*=6.22e-13 min^{-1}	Fitted
v120	Removal of secreted IL-10	*k120**[IL10]	*k120*=0.0015 min^{-1}	Fitted
v121	IRF1 promotes IL-12 production	*k121**[IRF1]^2	*k121*=5e-8 min^{-1}	Fitted
v122	Removal of secreted IL-12	*k122**[IL12]	*k122*=0.00033 min^{-1}	Fitted
v123	STAT1 induces CXCL9 synthesis	*k123**[pSTAT1D_n]^2	*k123*=1.67e-6 min^{-1}	Fitted
v124	Removal of secreted CXCL9	*k124**[CXCL9]	*k124*=0.0002 min^{-1}	Fitted

v125	STAT1/IRF9 complex induces CXCL10 mRNA production	*k125**[pSTAT1D/IRF9_n]^2	*k125*=1e-6 min^{-1}	Fitted
v126	CXCL10 mRNA degradation	*k126**[mCXCL10]	*k126*=0.0035 min^{-1}	(40)
v127	STAT1 and STAT6 induce SOCS1 production	*k127**[pSTAT1D_n]/([pSTAT1D_n]+*ka127*)*[pSTAT6D_n]/([pSTAT6D_n]+*kb127*)	*k127*=13342 min^{-1}, *ka127*=6000, *kb127*=500	Fitted
v128	Degradation of SOCS1	*k128**[SOCS1]	*k128*=0.004 min^{-1}	(45)
v129	STAT1 induces SOCS3 production	*k129**[pSTAT1D_n]/([pSTAT1D_n]+*ka129*)	*k129*=6571 min^{-1}, *ka129*=6000	Fitted
v130	Degradation of SOCS3	*k130**[SOCS3]	*k130*=0.05 min^{-1}	Fitted

Table S1. Complete list of model reactions and parameter values. Reactions are formulated mechanistically based on literature evidence (the labels *v#* here match with the labels in Figure S1 and Table S2). As shown in the last column, numerical values of model parameters (~140 in total) are either estimated from relevant published data (e.g. experimental measurements, prior models) or estimated computationally through whole-model optimization (‘fitted’). Certain parameter values are shared by more than one reaction fluxes (marked by * in the last column).

Table S2

Name of model node	Initial condition (in # of molecules)	Equation (d[x]/dt=)	Initial condition Refs.
VEGF	5223	v111-v112	Estimated from (46)
IFNγ	358	v37-v41	Estimated from (47)
IFNGR/JAK	20376	v40-v41	Total IFNGR estimated from (48, 49)
IFNGR	106	-v40+v48	Fitted
IFNγ/R	1	v50+v54-v51	Fitted
IFNγ/R/JAK	0	v41-v42-v43+v51-v52	Fitted
pIFNγ/R/JAK	0	v42-v44	Fitted
STAT1	397262	-v44+v59+v60	Total STAT1 estimated from (49, 50)
pIFNγ/R/JAK/STAT1	84	v44-v45	Fitted
pSTAT1	41	v45-v60-2*v55	Fitted
pSTAT1D	81	v55-v56-v95	Fitted
pSTAT1D_n	80	v56-v57-v97	Fitted
STAT1D_n	84	v57+v99-v58	Fitted
STAT1_n	1673	2*v58-v59	Fitted
SOCS1	3169	v127-v128-v17+v18+v21-v27+v28-v43+v49	Total SOCS1 estimated from (51)
IFNγ/R/JAK/SOCS1	9	v43-v49-v50	Fitted
IFNγ/R_lyso	1	v46-v47-v48+v49+v53	Fitted
SOCS1/JAK	3	v50+v19-v21	Fitted
IRF9	2674	v93-v94-v95-v98	Total IRF9 estimated from (49)
IRF9_n	27001	v98-v97+v99	Fitted
pSTAT1D/IRF9	26	v95-v96	Fitted
pSTAT1D/IRF9_n	203	v96-v99+v97	Fitted
SOCS3	1735	v129-v130-v23+v24+v26-v29+v30-v52+v53	Total SOCS3 estimated from (51)
IFNγ/R/JAK/SOCS3	3	v52-v53-v54	Fitted
SOCS3/JAK	8	v54+v25-v26	Fitted
IL4	288	v1-v7	Estimated from (52)
IL4R	16	v2-v6+v12	Fitted
IL4/R	0	v19+v25-v22	Fitted
IL4R/JAK	3478	v6-v7	Total IL4R estimated from (49)
IL4/R/JAK	0	v7-v8	Fitted
pIL4/R/JAK	0	v8-v9	Fitted
pIL4/R/JAK_i	0	v9-v10-v13+v14-v17+v22-v23	Fitted
IL4/R/JAK/SOCS1_i	0	v17-v18-v19	Fitted

IL4/R_lyso	0	*v10-v11-v12+v18+v20+v24+v28+v30*	Fitted
IL4/R/JAK/SOCS3_i	0	*v23-v24-v25*	Fitted
IL4/R/JAK/SOCS1	2	*v27-v28*	Fitted
IL4/R/JAK/SOCS3	0	*v29-v30*	Fitted
STAT6	59874	*v35+v36-v13*	Total STAT6 estimated from (49, 51)
pIL4/R/JAK/STAT6	0	*v13-v14*	Fitted
pSTAT6	26	*v14-v36-2*v31*	Fitted
pSTAT6D	1	*v31-v32*	Fitted
pSTAT6D_n	39	*v32-v33*	Fitted
STAT6D_n	0	*v33-v34*	Fitted
STAT6_n	16	*2*v34-v35*	Fitted
PTEN	64899	*v84-v85*	Estimated from (49)
IRF4	16460	*v100-v101*	Estimated from (49)
IL10	571	*v119-v120*	Estimated from (53)
JAK	99297	*v4-v5-v6+v10+v18-v22+v24+v28+v30-v40+v46+v49+v53*	Total JAK estimated from (49)
ARG1	875428	*v115-v116*	Estimated from (49)
miR3473	1180	*v79-v80-v81+v82*	Estimated from (54)
mPTEN	8	*v83-v86-v81*	Total mPTEN estimated from (55)
miR3473/mPTEN	6	*v81-v82*	Fitted
iNOS	538	*v113-v114*	Fitted
HIF1α	640	*v61-v63-v71+v72*	Total HIF1α estimated from (56)
HIF1α_n	178	*v63-v65*	Fitted
HIF1α/β_n	44	*v65*	Fitted
HIF1α_OH	42	*v71-v72-v73*	Fitted
HIF2α	878	*v62-v64-v74+v75*	Total HIF2α estimated from (56)
HIF2α_n	244	*v64-v66*	Fitted
HIF2α/β_n	61	*v66*	Fitted
HIF2α_OH	22	*v74-v75-v76*	Fitted
HIF1β_n	14897	*-v65-v66*	Total HIF1β estimated from (49)
PPARG	1437	*v102-v103+v104-v106*	Total PPARG estimated from (57)
aPPARG	782	*v103-v104-v105*	Fitted
IRF1	614	*v77-v78*	Estimated from (49)
PHD	5470	*v67-v68-v69*	Total PHD estimated from (49)
O_2	1.204e8	*Constant*	Estimated from (56)
O_2/aPHD	3088	*v70-v71-v74*	Fitted
aPHD	38846	*v69-v70+v71+v74*	Fitted

TNFα	619	*v117-v118*	Estimated from (58-60)
CXCL9	54	*v123-v124*	Fitted
mIRF9	9	*v91-v92-v89*	Total mIRF9 estimated from (61)
miR93/mIRF9	5	*v89-v90*	Fitted
miR93	841	*v87-v88-v89+v90*	Fitted
IL12	58	*v121-v122*	Estimated from (62)
mCXCL10	12	*v125-v126*	Estimated from (61)
AKT	198060	*-v15+v16*	Total AKT estimated from (49)
pAKT	11942	*v15-v16*	Fitted
IRG1	3522	*v107-v108*	Estimated from (49)
Itaconate	6.214e7	*v109-v110*	Estimated from (63)
IRF9T (repeated assignment)	29904	*[IRF9T]=[IRF9]+[IRF9_n]+ [pSTAT1D/IRF9]+[pSTAT1D/IRF9_n]*	Fitted

Table S2. Differential equations and initial conditions of all model nodes. A summary of the model equations and initial conditions in terms of absolute copy numbers of all 80 model nodes (proteins, RNAs, complexes, etc.) derived from the 34 functionally unique species. The initial levels (and also the steady state levels) of 31 out of 34 unique species are estimated and calibrated with respect to literature data (using both direct measurements and indirect observations), and the model is simulated in control condition without external stimulation until equilibrium to obtain the initial levels (e.g. copy numbers) of all 80 model nodes. A cell volume of 1 pL is assumed when doing unit conversion calculations for O_2 and itaconate (64, 65). To reduce model complexity, it is assumed that transcription factors and enzymes in Hill-type reactions are not consumed, mRNAs are not consumed during translation, and oxygen level is constant during each simulation run.

Table S3

Description of Data Used in Calibration	*PMIDs of Sources*
**IFN-γ module (in response to IFN-γ treatments unless noted otherwise below)**	
Surface-bound IFN-γ	2953810
Phosphorylation of JAK	12667213
Phosphorylation of STAT1	16473883, 26882544, 26299368, 28280036, 10490990
Expression of IRF-1	17293456, 18802049
Expression of iNOS	18655171, 9667738
TNFα secretion	8802049
IL-12 secretion	25950470
CXCL-9 secretion	25950470
CXCL-10 mRNA expression	25918247
Expression of miR-3473b	25092892
Itaconate expression	26829557
PTEN expression upon miR-3473b overexpression	25092892
HIF1α expression upon IFN-γ treatment and hypoxia	20194441
SOCS1 mRNA expression	17093501
SOCS3 mRNA expression	17093501
HIF2α expression upon IFN-γ treatment and hypoxia	20194441
**IL-4 module (in response to IL-4 treatments unless noted otherwise below)**	
STAT6 phosphorylation	27731330, 17093501, 26894960, 26883801, 27464342, 25175012
Phosphorylated STAT6 in nucleus	23913966
IRF-4 expression	29871928, 23287596, 20580461
AKT activation	26894960, 27731330, 27507812
PPARγ expression	29203644, 24385430
Arg-1 expression	23287596, 27117406, 23913966
Arg-1 activity	22348056
IL-10 secretion	28903394, 21753147
VEGF secretion	28842601
VEGF expression (intracellular)	28903394
TNFα secretion	28903394, 21753147
HIF2α expression upon IL-4 treatment and hypoxia	20194441
SOCS1 mRNA expression	17093501
HIF1α expression upon IL-4 treatment and hypoxia	20194441
**Hypoxia module (in response to hypoxia unless noted otherwise below)**	
HIF1a stabilization	24301659, 16533170, 20644254, 19454749
HIF2a stabilization	16533170, 20644254, 19454749
iNOS expression	28211523
Arg-1 expression	28211523

TNFα secretion	22566835
IFN-γ secretion	19234213
VEGF secretion	17065555
VEGF expression (intracellular)	28903394
miR-93 expression	28356443
IFN-γ secretion upon miR-93 overexpression and hypoxia	28356443
PHD2 expression	12912907
IRF-1 expression	11313373
IRF-9 mRNA expression	28356443
IRG-1 mRNA expression	28356443
IRG-1 mRNA expression upon miR-93 overexpression	28356443
TNFα secretion upon miR-93 overexpression and hypoxia	28356443

Table S3. Summary of literature sources used in model calibration. Listed here are the descriptions and PMIDs of all the literature sources from which the model calibration datasets were extracted.

Protocol S1: Additional information regarding model formulation and analysis

Model Formulation

Macrophage polarization is a complex, dynamic multi-pathway process with numerous feedbacks and cross-talks, which also makes its a highly suitable topic for systems biology modeling. Given the many relevant pathways and the large number of mechanistic details that govern macrophage polarization, it makes sense to approach this problem in a stepwise manner (e.g. first build a “beginner model”, then gradually enrich the model with more pathways of high importance in disease contexts). After careful analysis of literature knowledge, we ended up choosing three pathways (IFN-γ, IL-4, hypoxia) to model in the beginning step (as presented in this paper), since IFN-γ and IL-4 are representative inducers of M1 and M2 phenotypes and many experimental studies have tried to elucidate their downstream signaling and gene regulation mechanisms (which provided a wealth of quantitative data that can be used to calibrate our “beginner model”), and also because that hypoxia is a key signature in the disease areas that we are interested in (namely peripheral arterial disease and cancer) while its direct impact on macrophage polarization and connections with other macrophage pathways have not been systematically characterized before.

Model Calibration

During model calibration, values of some model parameters are derived directly from literature data and previous models (as shown in Table S1); for the remaining parameters with no literature reference, we put in tentative values first and then hand-tuned the entire model extensively (by adjusting parameter values and observing model response) until the model simulations achieved good visual agreements with all the respective calibration datasets (from the sources listed in Table S3) simultaneously. In the meantime, the initial conditions of “unique” species in the model have to stay within the allowed ranges (0.5x-2x of concentration values estimated from literature as listed in Table S2, except for total HIF-1α and HIF-2α which we set that their resting concentrations per cell should be less than a few nanomolar according to (56)). Then we performed preliminary sensitivity analysis (for three cases, IL-4 or IFN-γ or hypoxia stimulation) and collectively identified 101 parameters that have statistically significant ($p<0.05$) PRCC values. Among the 101 parameters, 82 had no literature reference and global optimization using

patternsearch in MATLAB was then performed for those 82 parameter values (with 0.5x-2x as the allowed ranges) with respect to all the calibration datasets (along with the initial condition checks in every iteration) to generate the final parameter values. We rounded the final values to three significant digits for all parameters (as listed in Table S1).

For the initial condition checks, we simulated the model for 100000 minutes to obtain species endpoint values (and check if they are within appropriate ranges as described above) and use these endpoint values as new initial conditions to generate simulations and calculate squared errors with respect to the literature data in every iteration of *patternsearch* optimization. Since we selected this very long time span, in each iteration these new initial conditions obtained would represent a set of species equilibrium states (of macrophages under normoxia without externally added stimuli) that can be compared with quantitative literature data.

Model Sensitivity Analysis and Uncertainty Quantification

For model sensitivity analysis, we used Latin Hypercube Sampling method with parameter ranges of 0.5x-2x to calculate the PRCC values (with p=0.05 as the cutoff for statistical significance) based on the algorithm and code published in (66). The output of interest in PRCC calculations are "M1/M2 scores", which are the multiplication of six M1 markers ([iNOS]*[IFNγ]*[TNFα]*[IL12]*[CXCL9]*[mCXCL10]; [IFNγ] is removed when calculating PRCCs in scenarios of IFN-γ stimulation) divided by the multiplication of three M2 markers ([ARG1]*[VEGF]*[IL10]). For uncertainty quantification, each of the 50 re-sampled datasets has 229 individual datapoints that covered all the experimental conditions used in model calibration. For each datapoint, we assumed its value is within a distinct normal distribution with a mean and a standard deviation (whenever possible, the mean and standard deviations are calculated from the corresponding literature data that we gathered; for datapoints that only had one value and no repeat, we considered that single value as the mean and assumed that the standard deviation equals to 10% of that value). Then the 229 datapoint values in each re-sampled dataset were compiled in order by generating random numbers from the 229 normal distributions. The 50 re-sampled datasets were then fed into the optimization algorithm to obtain 50 sets of new parameter estimates (as described in Materials and Methods).

References for Supporting Information

1. Majoros A, Platanitis E, Szappanos D, Cheon H, Vogl C, Shukla P, et al. Response to interferons and antibacterial innate immunity in the absence of tyrosine-phosphorylated STAT1. EMBO Rep. 2016;17(3):367-82.
2. Kim HS, Kim DC, Kim HM, Kwon HJ, Kwon SJ, Kang SJ, et al. STAT1 deficiency redirects IFN signalling toward suppression of TLR response through a feedback activation of STAT3. Sci Rep. 2015;5:13414.
3. Li X, Zhang Z, Li L, Gong W, Lazenby AJ, Swanson BJ, et al. Myeloid-derived cullin 3 promotes STAT3 phosphorylation by inhibiting OGT expression and protects against intestinal inflammation. J Exp Med. 2017;214(4):1093-109.
4. Ting LM, Kim AC, Cattamanchi A, Ernst JD. Mycobacterium tuberculosis inhibits IFN-gamma transcriptional responses without inhibiting activation of STAT1. J Immunol. 1999;163(7):3898-906.
5. Dickensheets H, Vazquez N, Sheikh F, Gingras S, Murray PJ, Ryan JJ, et al. Suppressor of cytokine signaling-1 is an IL-4-inducible gene in macrophages and feedback inhibits IL-4 signaling. Genes Immun. 2007;8(1):21-7.
6. Vila-del Sol V, Punzon C, Fresno M. IFN-gamma-induced TNF-alpha expression is regulated by interferon regulatory factors 1 and 8 in mouse macrophages. J Immunol. 2008;181(7):4461-70.
7. Ohata T, Fukuda K, Murakami A, Ohigashi H, Sugimura T, Wakabayashi K. Inhibition by 1'-acetoxychavicol acetate of lipopolysaccharide- and interferon-gamma-induced nitric oxide production through suppression of inducible nitric oxide synthase gene expression in RAW264 cells. Carcinogenesis. 1998;19(6):1007-12.
8. Wu C, Xue Y, Wang P, Lin L, Liu Q, Li N, et al. IFN-gamma primes macrophage activation by increasing phosphatase and tensin homolog via downregulation of miR-3473b. J Immunol. 2014;193(6):3036-44.
9. Takeda N, O'Dea EL, Doedens A, Kim JW, Weidemann A, Stockmann C, et al. Differential activation and antagonistic function of HIF-{alpha} isoforms in macrophages are essential for NO homeostasis. Genes Dev. 2010;24(5):491-501.
10. Wormald S, Zhang JG, Krebs DL, Mielke LA, Silver J, Alexander WS, et al. The comparative roles of suppressor of cytokine signaling-1 and -3 in the inhibition and desensitization of cytokine signaling. J Biol Chem. 2006;281(16):11135-43.
11. Covarrubias AJ, Aksoylar HI, Yu J, Snyder NW, Worth AJ, Iyer SS, et al. Akt-mTORC1 signaling regulates Acly to integrate metabolic input to control of macrophage activation. Elife. 2016;5.
12. Yao Y, Wang Y, Zhang Z, He L, Zhu J, Zhang M, et al. Chop Deficiency Protects Mice Against Bleomycin-induced Pulmonary Fibrosis by Attenuating M2 Macrophage Production. Mol Ther. 2016;24(5):915-25.
13. Rex J, Albrecht U, Ehlting C, Thomas M, Zanger UM, Sawodny O, et al. Model-Based Characterization of Inflammatory Gene Expression Patterns of Activated Macrophages. PLoS Comput Biol. 2016;12(7):e1005018.
14. Zhu L, Yang T, Li L, Sun L, Hou Y, Hu X, et al. TSC1 controls macrophage polarization to prevent inflammatory disease. Nat Commun. 2014;5:4696.
15. Sheldon KE, Shandilya H, Kepka-Lenhart D, Poljakovic M, Ghosh A, Morris SM, Jr. Shaping the murine macrophage phenotype: IL-4 and cyclic AMP synergistically activate the arginase I promoter. J Immunol. 2013;191(5):2290-8.
16. El Chartouni C, Schwarzfischer L, Rehli M. Interleukin-4 induced interferon regulatory factor (Irf) 4 participates in the regulation of alternative macrophage priming. Immunobiology. 2010;215(9-10):821-5.
17. Kimura T, Nada S, Takegahara N, Okuno T, Nojima S, Kang S, et al. Polarization of M2 macrophages requires Lamtor1 that integrates cytokine and amino-acid signals. Nat Commun. 2016;7:13130.

18. McCormick SM, Gowda N, Fang JX, Heller NM. Suppressor of Cytokine Signaling (SOCS)1 Regulates Interleukin-4 (IL-4)-activated Insulin Receptor Substrate (IRS)-2 Tyrosine Phosphorylation in Monocytes and Macrophages via the Proteasome. J Biol Chem. 2016;291(39):20574-87.
19. Zanin RF, Braganhol E, Bergamin LS, Campesato LF, Filho AZ, Moreira JC, et al. Differential macrophage activation alters the expression profile of NTPDase and ecto-5'-nucleotidase. PLoS One. 2012;7(2):e31205.
20. Zhang W, Xu W, Xiong S. Macrophage differentiation and polarization via phosphatidylinositol 3-kinase/Akt-ERK signaling pathway conferred by serum amyloid P component. J Immunol. 2011;187(4):1764-77.
21. Lee C, Bae SS, Joo H, Bae H. Melittin suppresses tumor progression by regulating tumor-associated macrophages in a Lewis lung carcinoma mouse model. Oncotarget. 2017;8(33):54951-65.
22. Fang HY, Hughes R, Murdoch C, Coffelt SB, Biswas SK, Harris AL, et al. Hypoxia-inducible factors 1 and 2 are important transcriptional effectors in primary macrophages experiencing hypoxia. Blood. 2009;114(4):844-59.
23. Berra E, Benizri E, Ginouves A, Volmat V, Roux D, Pouyssegur J. HIF prolyl-hydroxylase 2 is the key oxygen sensor setting low steady-state levels of HIF-1alpha in normoxia. EMBO J. 2003;22(16):4082-90.
24. Carta L, Pastorino S, Melillo G, Bosco MC, Massazza S, Varesio L. Engineering of macrophages to produce IFN-gamma in response to hypoxia. J Immunol. 2001;166(9):5374-80.
25. Mei Y, Thompson MD, Shiraishi Y, Cohen RA, Tong X. Sarcoplasmic/endoplasmic reticulum Ca2+ ATPase C674 promotes ischemia- and hypoxia-induced angiogenesis via coordinated endothelial cell and macrophage function. J Mol Cell Cardiol. 2014;76:275-82.
26. Ganta VC, Choi MH, Kutateladze A, Fox TE, Farber CR, Annex BH. A MicroRNA93-Interferon Regulatory Factor-9-Immunoresponsive Gene-1-Itaconic Acid Pathway Modulates M2-Like Macrophage Polarization to Revascularize Ischemic Muscle. Circulation. 2017;135(24):2403-25.
27. Cambridge SB, Gnad F, Nguyen C, Bermejo JL, Kruger M, Mann M. Systems-wide proteomic analysis in mammalian cells reveals conserved, functional protein turnover. J Proteome Res. 2011;10(12):5275-84.
28. Bankaitis KV, Fingleton B. Targeting IL4/IL4R for the treatment of epithelial cancer metastasis. Clin Exp Metastasis. 2015;32(8):847-56.
29. Gao T, Furnari F, Newton AC. PHLPP: a phosphatase that directly dephosphorylates Akt, promotes apoptosis, and suppresses tumor growth. Mol Cell. 2005;18(1):13-24.
30. Hanson EM, Dickensheets H, Qu CK, Donnelly RP, Keegan AD. Regulation of the dephosphorylation of Stat6. Participation of Tyr-713 in the interleukin-4 receptor alpha, the tyrosine phosphatase SHP-1, and the proteasome. J Biol Chem. 2003;278(6):3903-11.
31. Chen HC, Reich NC. Live cell imaging reveals continuous STAT6 nuclear trafficking. J Immunol. 2010;185(1):64-70.
32. Londino JD, Gulick DL, Lear TB, Suber TL, Weathington NM, Masa LS, et al. Post-translational modification of the interferon-gamma receptor alters its stability and signaling. Biochem J. 2017;474(20):3543-57.
33. Ping Z, Qi J, Sun Y, Lu G, Shi Y, Wang X, et al. Crystal structure of the interferon gamma receptor alpha chain from chicken reveals an undetected extra helix compared with the human counterparts. J Interferon Cytokine Res. 2014;34(1):41-51.
34. Nickoloff BJ. Binding of 125I-gamma interferon to cultured human keratinocytes. J Invest Dermatol. 1987;89(2):132-5.
35. Zhu W, Mustelin T, David M. Arginine methylation of STAT1 regulates its dephosphorylation by T cell protein tyrosine phosphatase. J Biol Chem. 2002;277(39):35787-90.
36. Zhao C, Isenberg JS, Popel AS. Transcriptional and Post-Transcriptional Regulation of Thrombospondin-1 Expression: A Computational Model. PLoS Comput Biol. 2017;13(1):e1005272.

37. Eckhardt I, Weigert A, Fulda S. Identification of IRF1 as critical dual regulator of Smac mimetic-induced apoptosis and inflammatory cytokine response. Cell Death Dis. 2014;5:e1562.
38. Marzi MJ, Ghini F, Cerruti B, de Pretis S, Bonetti P, Giacomelli C, et al. Degradation dynamics of microRNAs revealed by a novel pulse-chase approach. Genome Res. 2016;26(4):554-65.
39. Wang X, Li Y, Xu X, Wang YH. Toward a system-level understanding of microRNA pathway via mathematical modeling. Biosystems. 2010;100(1):31-8.
40. Sharova LV, Sharov AA, Nedorezov T, Piao Y, Shaik N, Ko MS. Database for mRNA half-life of 19 977 genes obtained by DNA microarray analysis of pluripotent and differentiating mouse embryonic stem cells. DNA Res. 2009;16(1):45-58.
41. Waite KJ, Floyd ZE, Arbour-Reily P, Stephens JM. Interferon-gamma-induced regulation of peroxisome proliferator-activated receptor gamma and STATs in adipocytes. J Biol Chem. 2001;276(10):7062-8.
42. Kolodziejski PJ, Koo JS, Eissa NT. Regulation of inducible nitric oxide synthase by rapid cellular turnover and cotranslational down-regulation by dimerization inhibitors. Proc Natl Acad Sci U S A. 2004;101(52):18141-6.
43. Setty BA, Jin Y, Houghton PJ, Yeager ND, Gross TG, Nelin LD. Hypoxic Proliferation of Osteosarcoma Cells Depends on Arginase II. Cell Physiol Biochem. 2016;39(2):802-13.
44. Schimke RT. The Importance of Both Synthesis and Degradation in the Control of Arginase Levels in Rat Liver. J Biol Chem. 1964;239:3808-17.
45. Zimmer J, Weitnauer M, Boutin S, Kublbeck G, Thiele S, Walker P, et al. Nuclear Localization of Suppressor of Cytokine Signaling-1 Regulates Local Immunity in the Lung. Front Immunol. 2016;7:514.
46. Eubank TD, Roda JM, Liu H, O'Neil T, Marsh CB. Opposing roles for HIF-1alpha and HIF-2alpha in the regulation of angiogenesis by mononuclear phagocytes. Blood. 2011;117(1):323-32.
47. Yu Y, Cui Y, Zhao Y, Liu S, Song G, Jiao P, et al. The binding capability of plasma phospholipid transfer protein, but not HDL pool size, is critical to repress LPS induced inflammation. Sci Rep. 2016;6:20845.
48. Crouse JA, Mitchell WM. Interferon-gamma receptor: mRNA half-life, receptor mass, and abundance on A431 human epidermoid carcinoma cells. J Interferon Res. 1992;12(1):23-5.
49. Azimifar SB, Nagaraj N, Cox J, Mann M. Cell-type-resolved quantitative proteomics of murine liver. Cell Metab. 2014;20(6):1076-87.
50. Precious BL, Carlos TS, Goodbourn S, Randall RE. Catalytic turnover of STAT1 allows PIV5 to dismantle the interferon-induced anti-viral state of cells. Virology. 2007;368(1):114-21.
51. Brenes A, Afzal V, Kent R, Lamond AI. The Encyclopedia of Proteome Dynamics: a big data ecosystem for (prote)omics. Nucleic Acids Res. 2018;46(D1):D1202-D9.
52. Mukherjee S, Chen LY, Papadimos TJ, Huang S, Zuraw BL, Pan ZK. Lipopolysaccharide-driven Th2 cytokine production in macrophages is regulated by both MyD88 and TRAM. J Biol Chem. 2009;284(43):29391-8.
53. Yang HL, Zhou WJ, Chang KK, Mei J, Huang LQ, Wang MY, et al. The crosstalk between endometrial stromal cells and macrophages impairs cytotoxicity of NK cells in endometriosis by secreting IL-10 and TGF-beta. Reproduction. 2017;154(6):815-25.
54. Bissels U, Wild S, Tomiuk S, Holste A, Hafner M, Tuschl T, et al. Absolute quantification of microRNAs by using a universal reference. RNA. 2009;15(12):2375-84.
55. Schwanhausser B, Busse D, Li N, Dittmar G, Schuchhardt J, Wolf J, et al. Global quantification of mammalian gene expression control. Nature. 2011;473(7347):337-42.
56. Tuckerman JR, Zhao Y, Hewitson KS, Tian YM, Pugh CW, Ratcliffe PJ, et al. Determination and comparison of specific activity of the HIF-prolyl hydroxylases. FEBS Lett. 2004;576(1-2):145-50.
57. Simicevic J, Schmid AW, Gilardoni PA, Zoller B, Raghav SK, Krier I, et al. Absolute quantification of transcription factors during cellular differentiation using multiplexed targeted proteomics. Nat Methods. 2013;10(6):570-6.

58. Xaus J, Comalada M, Valledor AF, Lloberas J, Lopez-Soriano F, Argiles JM, et al. LPS induces apoptosis in macrophages mostly through the autocrine production of TNF-alpha. Blood. 2000;95(12):3823-31.

59. Barbera-Cremades M, Gomez AI, Baroja-Mazo A, Martinez-Alarcon L, Martinez CM, de Torre-Minguela C, et al. P2X7 Receptor Induces Tumor Necrosis Factor-alpha Converting Enzyme Activation and Release to Boost TNF-alpha Production. Front Immunol. 2017;8:862.

60. Cudejko C, Wouters K, Fuentes L, Hannou SA, Paquet C, Bantubungi K, et al. p16INK4a deficiency promotes IL-4-induced polarization and inhibits proinflammatory signaling in macrophages. Blood. 2011;118(9):2556-66.

61. Marinov GK, Williams BA, McCue K, Schroth GP, Gertz J, Myers RM, et al. From single-cell to cell-pool transcriptomes: stochasticity in gene expression and RNA splicing. Genome Res. 2014;24(3):496-510.

62. Su Z, Yang R, Zhang W, Xu L, Zhong Y, Yin Y, et al. The synergistic interaction between the calcineurin B subunit and IFN-gamma enhances macrophage antitumor activity. Cell Death Dis. 2015;6:e1740.

63. Michelucci A, Cordes T, Ghelfi J, Pailot A, Reiling N, Goldmann O, et al. Immune-responsive gene 1 protein links metabolism to immunity by catalyzing itaconic acid production. Proc Natl Acad Sci U S A. 2013;110(19):7820-5.

64. Krombach F, Munzing S, Allmeling AM, Gerlach JT, Behr J, Dorger M. Cell size of alveolar macrophages: an interspecies comparison. Environ Health Perspect. 1997;105 Suppl 5:1261-3.

65. Swanson JA, Lee M, Knapp PE. Cellular dimensions affecting the nucleocytoplasmic volume ratio. J Cell Biol. 1991;115(4):941-8.

66. Marino S, Hogue IB, Ray CJ, Kirschner DE. A methodology for performing global uncertainty and sensitivity analysis in systems biology. J Theor Biol. 2008;254(1):178-96.
