# Supplementary material for: A mechanistic integrative computational model of macrophage polarization: Implications in human pathophysiology
Source: PLoS Comput Biol. 2019 Nov 18;15(11):e1007468. doi: 10.1371/journal.pcbi.1007468 (PMC6860420; doi:10.1371/journal.pcbi.1007468)
Supplement: S1 Fig — (PDF) [file pcbi.1007468.s002.pdf]

Figure S1

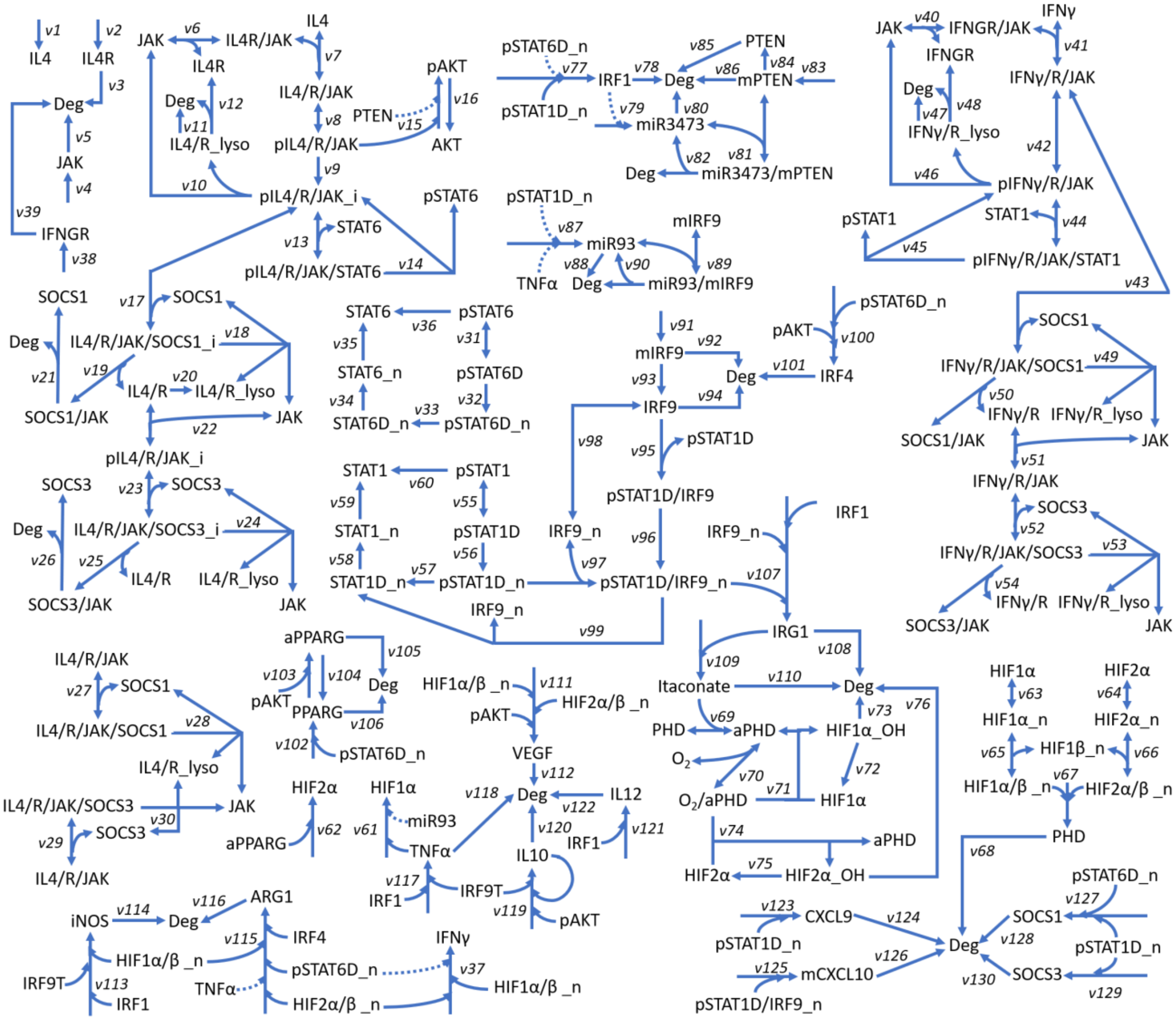

Figure S1. Complete model diagram with all nodes and reactions. Reaction rates are labeled (*v#*) and correspond to the rate laws described in detail in Table S1. For the names of model nodes (reaction species): 'X/Y' – complex formed by X and Y, 'pX' – phosphorylated form of species X, 'X_n' – species X in nucleus, 'mX' – mRNA of species X, 'aX' – species X in its activated form, 'Deg' – degradation, dashed arrows – inhibition. Reaction descriptions, parameters, rates and differential equations for all model nodes are summarized in Tables S1 and S2.
