# Supplementary material for: A mechanistic integrative computational model of macrophage polarization: Implications in human pathophysiology
Source: PLoS Comput Biol. 2019 Nov 18;15(11):e1007468. doi: 10.1371/journal.pcbi.1007468 (PMC6860420; doi:10.1371/journal.pcbi.1007468)
Supplement: S2 Fig — (A-I) Additional model calibration data of IFN-γ-driven pathway. (PDF) [file pcbi.1007468.s003.pdf]

Figure S2

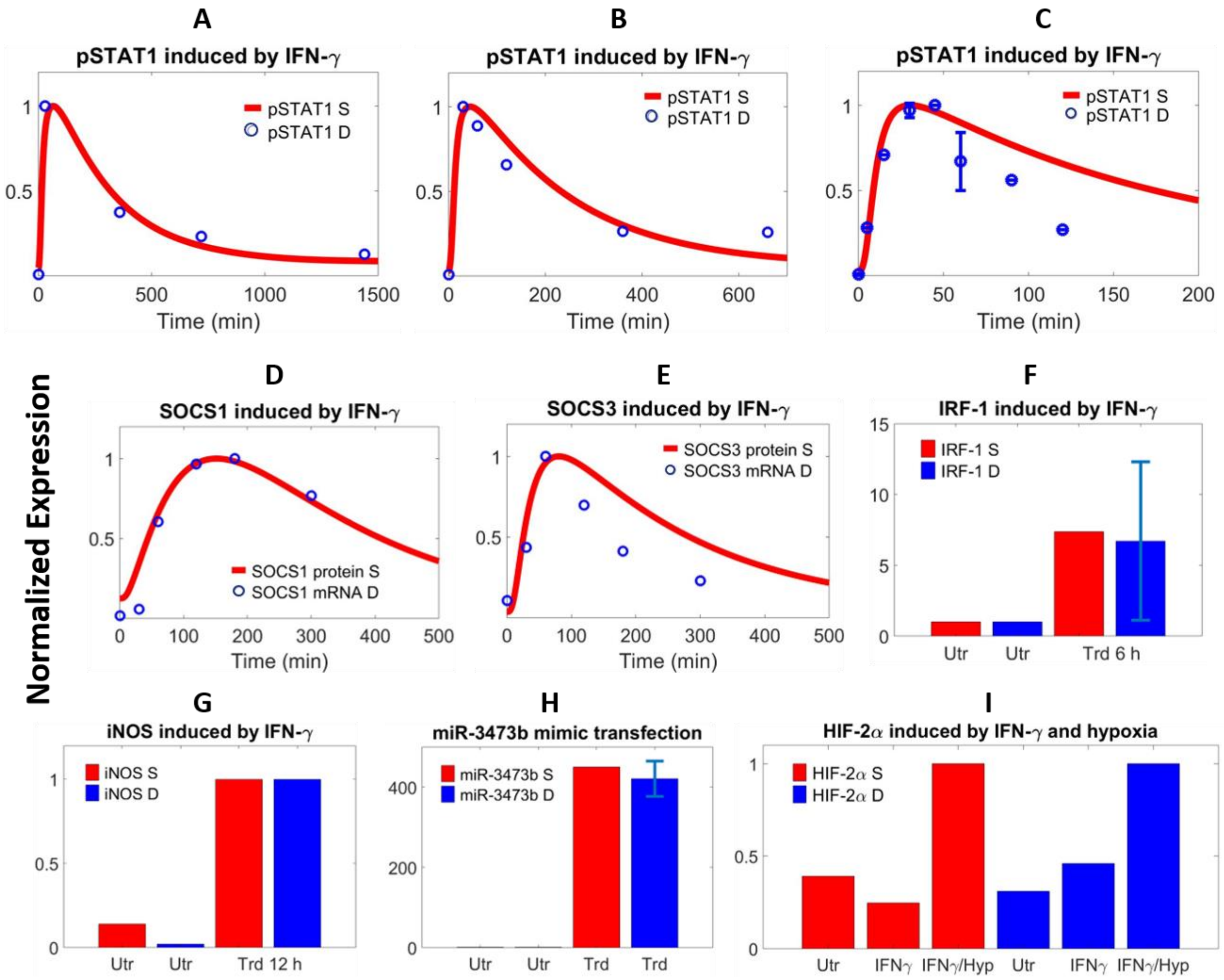

Figure S2. Additional model calibration data of IFN-γ-driven pathway. Comparisons between model simulations and literature experimental data of IFN-γ-induced (A-C) STAT1 activation at 5 ng/ml (1), 10 ng/ml (2), and 20 ng/ml of IFN-γ (3, 4), (D-E) upregulation of SOCS1 and SOCS3 (data is mRNA expression) (5), (F) increase in IRF-1 expression at 6 h (6), and (G) increase in iNOS expression at 12 h (7). (H) Data and simulated expression level of miR-3473b at 24 h after mimic transfection (8). (I) Hypoxia in combination with IFN-γ can significantly induce HIF-2α expression (for IFN-γ alone, simulation suggested a mild decrease while data suggested an insignificant increase in HIF-2α) (9). (A-I) All experimental data are measured in macrophage cell lines and values are for protein levels unless noted otherwise. Y-axes show normalized expression respectively (A-E: simulations and data are normalized to the maximum expression; F, H: normalized to the no-treatment/time 0 expression; G: normalized to the expression at 12 h; I: normalized to the expression under IFN-γ treatment with hypoxia). (D-E) For induction of SOCS1/3, data in terms of SOCS1/3 mRNA expression are compared with simulation (SOCS1/3 protein level), given that SOCS proteins are highly labile (direct protein measurements are scarce) and that Wormald et al. reported a tight temporal correlation between signaling-induced expression of SOCS1/3 protein and mRNA (10). S – simulation, D – literature data, Utr – untreated, Trd – IFN-γ treated, Hyp – hypoxia.

References

1. Majoros A, Platanitis E, Szappanos D, Cheon H, Vogl C, Shukla P, et al. Response to interferons and antibacterial innate immunity in the absence of tyrosine-phosphorylated STAT1. EMBO Rep. 2016;17(3):367-82.
2. Kim HS, Kim DC, Kim HM, Kwon HJ, Kwon SJ, Kang SJ, et al. STAT1 deficiency redirects IFN signalling toward suppression of TLR response through a feedback activation of STAT3. Sci Rep. 2015;5:13414.
3. Li X, Zhang Z, Li L, Gong W, Lazenby AJ, Swanson BJ, et al. Myeloid-derived cullin 3 promotes STAT3 phosphorylation by inhibiting OGT expression and protects against intestinal inflammation. J Exp Med. 2017;214(4):1093-109.
4. Ting LM, Kim AC, Cattamanchi A, Ernst JD. Mycobacterium tuberculosis inhibits IFN-gamma transcriptional responses without inhibiting activation of STAT1. J Immunol. 1999;163(7):3898-906.
5. Dickensheets H, Vazquez N, Sheikh F, Gingras S, Murray PJ, Ryan JJ, et al. Suppressor of cytokine signaling-1 is an IL-4-inducible gene in macrophages and feedback inhibits IL-4 signaling. Genes Immun. 2007;8(1):21-7.
6. Vila-del Sol V, Punzon C, Fresno M. IFN-gamma-induced TNF-alpha expression is regulated by interferon regulatory factors 1 and 8 in mouse macrophages. J Immunol. 2008;181(7):4461-70.
7. Ohata T, Fukuda K, Murakami A, Ohigashi H, Sugimura T, Wakabayashi K. Inhibition by 1'-acetoxychavicol acetate of lipopolysaccharide- and interferon-gamma-induced nitric oxide production through suppression of inducible nitric oxide synthase gene expression in RAW264 cells. Carcinogenesis. 1998;19(6):1007-12.
8. Wu C, Xue Y, Wang P, Lin L, Liu Q, Li N, et al. IFN-gamma primes macrophage activation by increasing phosphatase and tensin homolog via downregulation of miR-3473b. J Immunol. 2014;193(6):3036-44.
9. Takeda N, O'Dea EL, Doedens A, Kim JW, Weidemann A, Stockmann C, et al. Differential activation and antagonistic function of HIF-{alpha} isoforms in macrophages are essential for NO homeostasis. Genes Dev. 2010;24(5):491-501.
10. Wormald S, Zhang JG, Krebs DL, Mielke LA, Silver J, Alexander WS, et al. The comparative roles of suppressor of cytokine signaling-1 and -3 in the inhibition and desensitization of cytokine signaling. J Biol Chem. 2006;281(16):11135-43.
