# Supplementary material for: A mechanistic integrative computational model of macrophage polarization: Implications in human pathophysiology
Source: PLoS Comput Biol. 2019 Nov 18;15(11):e1007468. doi: 10.1371/journal.pcbi.1007468 (PMC6860420; doi:10.1371/journal.pcbi.1007468)
Supplement: S3 Fig — (A-N) Additional model calibration data of IL-4-driven pathway. (PDF) [file pcbi.1007468.s004.pdf]

Figure S3

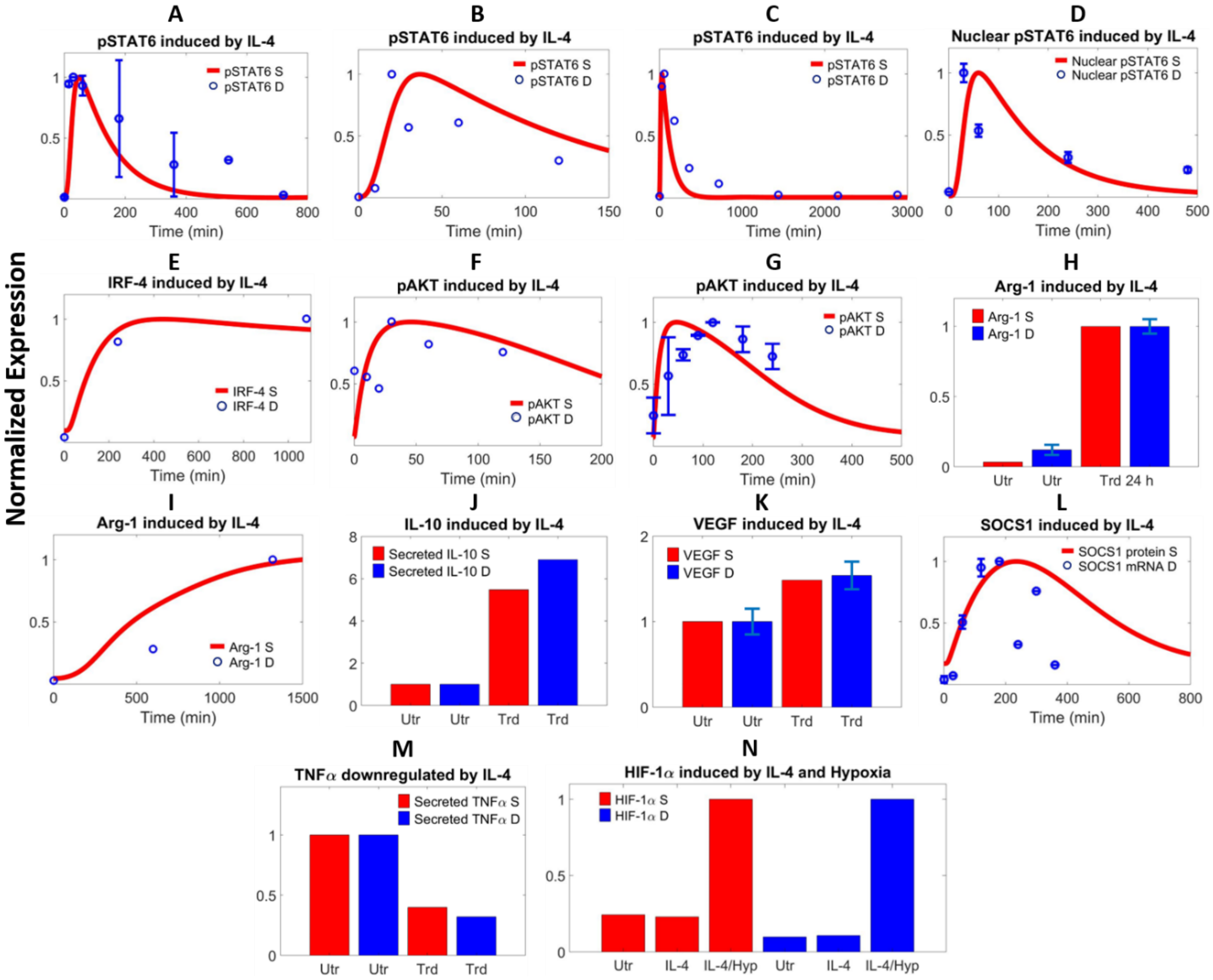

Figure S3. Additional model calibration data of IL-4-driven pathway. Comparisons between model simulations and published experimental data of IL-4-induced (A-C) STAT6 activation at 10 ng/ml (1-3), 50 ng/ml (4) and 100 nM IL-4 (5), (D) phosphorylated STAT6 in nucleus (6), (E) increase in IRF-4 expression (7), (F-G) AKT activation at 50 ng/ml (8) and 20 ng/ml IL-4 (9), (H-I) increase in Arg-1 production (6, 10), (J) increase in IL-10 production at 24 h (11), (K) increase in VEGF production (12), (L) SOCS1 induction (2), and (M) inhibition of TNFα production at 24 h (11). (N) Simulation and data show that hypoxia can stabilize HIF-1α protein while IL-4 stimulation has no effect (13). (A-N) All experimental data are measured in macrophage cell lines and values are for protein levels unless noted otherwise. Y-axes show normalized expression respectively (A-G, I, L: simulations and data are normalized to the maximum expression; J, K, M: normalized to the no-treatment/time 0 expression; H: normalized to the expression at 24 h; N: normalized to the expression under IL-4 treatment with hypoxia). (H) For Arg-1 production, data in terms of Arg-1 activity (formation of urea from arginine) is compared with simulation (Arg-1 protein level). (K) For VEGF production, data in terms of intracellular VEGF level is compared with simulation (secreted VEGF level). (L) For SOCS1 induction, data in terms of

SOCS1 mRNA level is compared with simulation (SOCS1 protein level), given the reasoning stated in Figures S2D-E. S – simulation, D – literature data, Utr – untreated, Trd – IL-4 treated, Hyp – hypoxia.

References

1. Covarrubias AJ, Aksoylar HI, Yu J, Snyder NW, Worth AJ, Iyer SS, et al. Akt-mTORC1 signaling regulates Acly to integrate metabolic input to control of macrophage activation. Elife. 2016;5.
2. Dickensheets H, Vazquez N, Sheikh F, Gingras S, Murray PJ, Ryan JJ, et al. Suppressor of cytokine signaling-1 is an IL-4-inducible gene in macrophages and feedback inhibits IL-4 signaling. Genes Immun. 2007;8(1):21-7.
3. Yao Y, Wang Y, Zhang Z, He L, Zhu J, Zhang M, et al. Chop Deficiency Protects Mice Against Bleomycin-induced Pulmonary Fibrosis by Attenuating M2 Macrophage Production. Mol Ther. 2016;24(5):915-25.
4. Rex J, Albrecht U, Ehlting C, Thomas M, Zanger UM, Sawodny O, et al. Model-Based Characterization of Inflammatory Gene Expression Patterns of Activated Macrophages. PLoS Comput Biol. 2016;12(7):e1005018.
5. Zhu L, Yang T, Li L, Sun L, Hou Y, Hu X, et al. TSC1 controls macrophage polarization to prevent inflammatory disease. Nat Commun. 2014;5:4696.
6. Sheldon KE, Shandilya H, Kepka-Lenhart D, Poljakovic M, Ghosh A, Morris SM, Jr. Shaping the murine macrophage phenotype: IL-4 and cyclic AMP synergistically activate the arginase I promoter. J Immunol. 2013;191(5):2290-8.
7. El Chartouni C, Schwarzfischer L, Rehli M. Interleukin-4 induced interferon regulatory factor (Irf) 4 participates in the regulation of alternative macrophage priming. Immunobiology. 2010;215(9-10):821-5.
8. Kimura T, Nada S, Takegahara N, Okuno T, Nojima S, Kang S, et al. Polarization of M2 macrophages requires Lamtor1 that integrates cytokine and amino-acid signals. Nat Commun. 2016;7:13130.
9. McCormick SM, Gowda N, Fang JX, Heller NM. Suppressor of Cytokine Signaling (SOCS)1 Regulates Interleukin-4 (IL-4)-activated Insulin Receptor Substrate (IRS)-2 Tyrosine Phosphorylation in Monocytes and Macrophages via the Proteasome. J Biol Chem. 2016;291(39):20574-87.
10. Zanin RF, Braganhol E, Bergamin LS, Campesato LF, Filho AZ, Moreira JC, et al. Differential macrophage activation alters the expression profile of NTPDase and ecto-5'-nucleotidase. PLoS One. 2012;7(2):e31205.
11. Zhang W, Xu W, Xiong S. Macrophage differentiation and polarization via phosphatidylinositol 3-kinase/Akt-ERK signaling pathway conferred by serum amyloid P component. J Immunol. 2011;187(4):1764-77.
12. Lee C, Bae SS, Joo H, Bae H. Melittin suppresses tumor progression by regulating tumor-associated macrophages in a Lewis lung carcinoma mouse model. Oncotarget. 2017;8(33):54951-65.
13. Takeda N, O'Dea EL, Doedens A, Kim JW, Weidemann A, Stockmann C, et al. Differential activation and antagonistic function of HIF-{alpha} isoforms in macrophages are essential for NO homeostasis. Genes Dev. 2010;24(5):491-501.
