# Supplementary material for: A mechanistic integrative computational model of macrophage polarization: Implications in human pathophysiology
Source: PLoS Comput Biol. 2019 Nov 18;15(11):e1007468. doi: 10.1371/journal.pcbi.1007468 (PMC6860420; doi:10.1371/journal.pcbi.1007468)
Supplement: S4 Fig — (A-I) Additional model calibration data of hypoxia-driven pathway. (PDF) [file pcbi.1007468.s005.pdf]

Figure S4

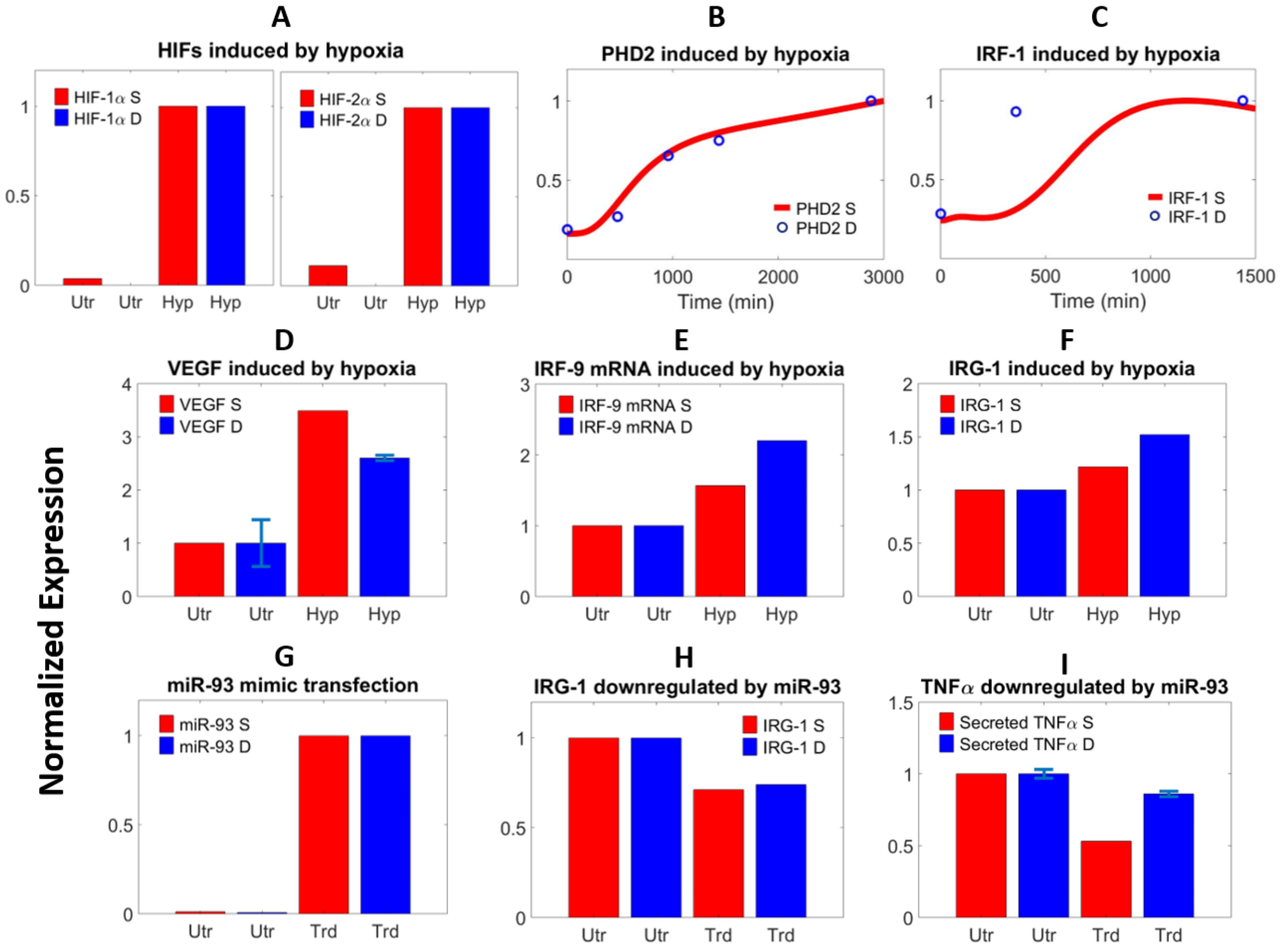

Figure S4. Additional model calibration data of hypoxia-driven pathway. Comparisons between model simulations and literature experimental data of hypoxia-induced (A) stabilization of HIF-1α and HIF-2α at 18 h (1), (B) PHD upregulation (2), (C) IRF-1 induction (3), (D) VEGF production at 24 h (4), (E) de-suppression of IRF-9 (5) and (F) downstream upregulation of IRG-1 at 12 h (5). (G) Data and simulated expression level of miR-93 before and after 24 h of mimic transfection, which leads to downregulation of (H) IRG-1 abundance at 24 h (5). (I) Transfection of miR-93 mimic decreases TNFα production at 12 h under hypoxia (5). (A-I) All experimental data are measured in macrophage cell lines (except for B, which is in Hela cells) and values are for protein levels unless noted otherwise. Y-axes show normalized expression respectively (A: simulation and data are normalized to the expression under hypoxia; B, C: normalized to the maximum expression; D, E, F, H: normalized to the no-treatment expression; G: normalized to the miR-93 expression at 24 h after transfection; I: normalized to the expression under 12 h of hypoxia without miR-93 mimic). (D) For VEGF production, data in terms of intracellular VEGF level is compared with simulation (secreted VEGF level). (F and H) For IRG-1 regulation, data in terms of IRG-1 mRNA level is compared with simulation (IRG-1 protein level). S – simulation, D – literature data, Utr – untreated, Trd – treated with miR-93 mimic, Hyp – hypoxia.

References

1. Fang HY, Hughes R, Murdoch C, Coffelt SB, Biswas SK, Harris AL, et al. Hypoxia-inducible factors 1 and 2 are important transcriptional effectors in primary macrophages experiencing hypoxia. Blood. 2009;114(4):844-59.
2. Berra E, Benizri E, Ginouves A, Volmat V, Roux D, Pouyssegur J. HIF prolyl-hydroxylase 2 is the key oxygen sensor setting low steady-state levels of HIF-1alpha in normoxia. EMBO J. 2003;22(16):4082-90.
3. Carta L, Pastorino S, Melillo G, Bosco MC, Massazza S, Varesio L. Engineering of macrophages to produce IFN-gamma in response to hypoxia. J Immunol. 2001;166(9):5374-80.
4. Mei Y, Thompson MD, Shiraishi Y, Cohen RA, Tong X. Sarcoplasmic/endoplasmic reticulum Ca2+ ATPase C674 promotes ischemia- and hypoxia-induced angiogenesis via coordinated endothelial cell and macrophage function. J Mol Cell Cardiol. 2014;76:275-82.
5. Ganta VC, Choi MH, Kutateladze A, Fox TE, Farber CR, Annex BH. A MicroRNA93-Interferon Regulatory Factor-9-Immunoresponsive Gene-1-Itaconic Acid Pathway Modulates M2-Like Macrophage Polarization to Revascularize Ischemic Muscle. Circulation. 2017;135(24):2403-25.
