# Supplementary material for: A mechanistic integrative computational model of macrophage polarization: Implications in human pathophysiology
Source: PLoS Comput Biol. 2019 Nov 18;15(11):e1007468. doi: 10.1371/journal.pcbi.1007468 (PMC6860420; doi:10.1371/journal.pcbi.1007468)
Supplement: S5 Fig — (A-J) Additional in silico investigation of pathway feedback within M1-M2 network. (PDF) [file pcbi.1007468.s006.pdf]

Figure S5

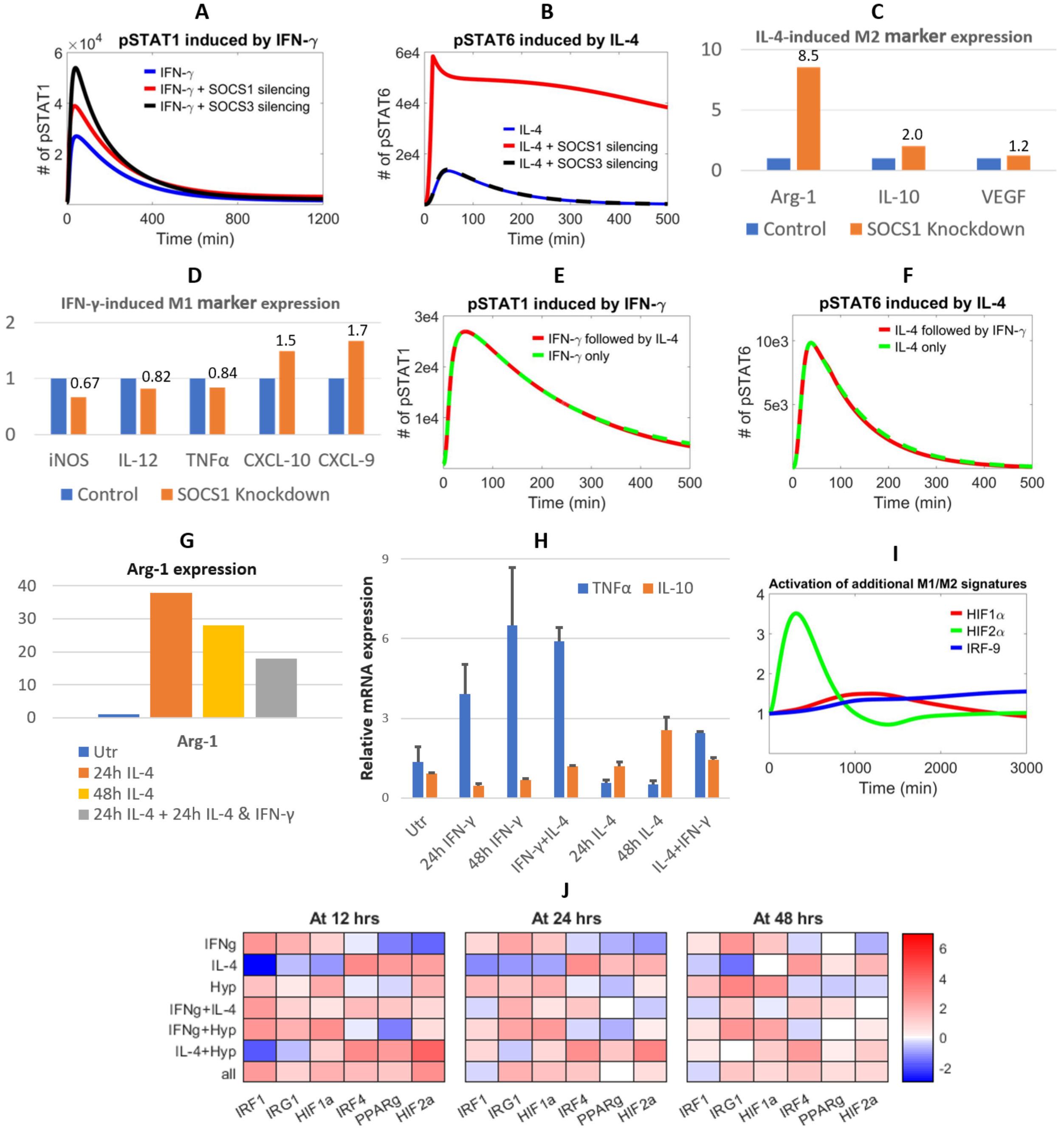

Figure S5. Additional *in silico* investigation of pathway feedback within M1-M2 network. (A) Silencing of SOCS1 or SOCS3 in macrophages can promote activation of STAT1 by IFN-γ. (B) SOCS1 silencing, but not SOCS3 silencing, can markedly boost STAT6 activation by IL-4. (A-B) Silencing is modeled as 0x initial level with 0x production. Knockdown of SOCS1 (modeled as 0.3x initial level with 0.3x production) (C) promotes IL-4-induced M2 marker expression and (D) differentially influences M1 marker expression in response to IFN-γ. In the scenarios of IFN-γ stimulation followed by the addition of IL-4 (at 4 h), or IL-4 stimulation followed by the addition of IFN-γ (at 1 hr), there is no obvious change of (E) STAT1 or (F) STAT6 activation. (G) The addition of a second stimulus IFN-γ (at 24 h post IL-4 stimulation) can antagonize the expression pattern of Arg-1 induced by IL-4. (H) RT-qPCR analysis

(results presented as mean + SEM, n=3) of TNFα and IL-10 gene expression in THP-1 cells stimulated with 24 and 48 h of IFN-γ (or IL-4), and 24 h of IFN-γ (or IL-4) then another 24 h of IFN-γ plus IL-4 (labeled as IFNγ+IL-4 or IL-4+IFNγ). (I) Temporal expression profiles of HIFs and IRF-9 when macrophages are stimulated with IFN-γ and IL-4 simultaneously. (J) Temporal relative protein expression patterns of six M1-M2 signature transcription factors in macrophages under seven different stimulation conditions (‘A+B’ means simultaneous stimulation, ‘all’ means IFN-γ+IL-4+hypoxia, all expression levels are normalized to the untreated/time 0 levels and then log2 transformed). (A-J) All simulation results are protein levels (except CXCL10 is mRNA level). (C,D,G,I) Y-axes show relative expression (normalized to untreated/control/time 0 levels). Simulated treatment doses are 10 ng/ml IFN-γ and 10 ng/ml IL-4 for (A-D), 10 ng/ml IFN-γ and 20 ng/ml IL-4 for (E-F), 20 ng/ml IFN-γ and 20 ng/ml IL-4 for (G), 10 ng/ml IFN-γ and 5 ng/ml IL-4 for (I-J). Utr – untreated, Hyp – hypoxia (2% oxygen for J).
