# Supplementary material for: A mechanistic integrative computational model of macrophage polarization: Implications in human pathophysiology
Source: PLoS Comput Biol. 2019 Nov 18;15(11):e1007468. doi: 10.1371/journal.pcbi.1007468 (PMC6860420; doi:10.1371/journal.pcbi.1007468)
Supplement: S6 Fig — (A-E) Temporal response of M2 markers and transcription factors under hypoxia. (PDF) [file pcbi.1007468.s007.pdf]

Figure S6

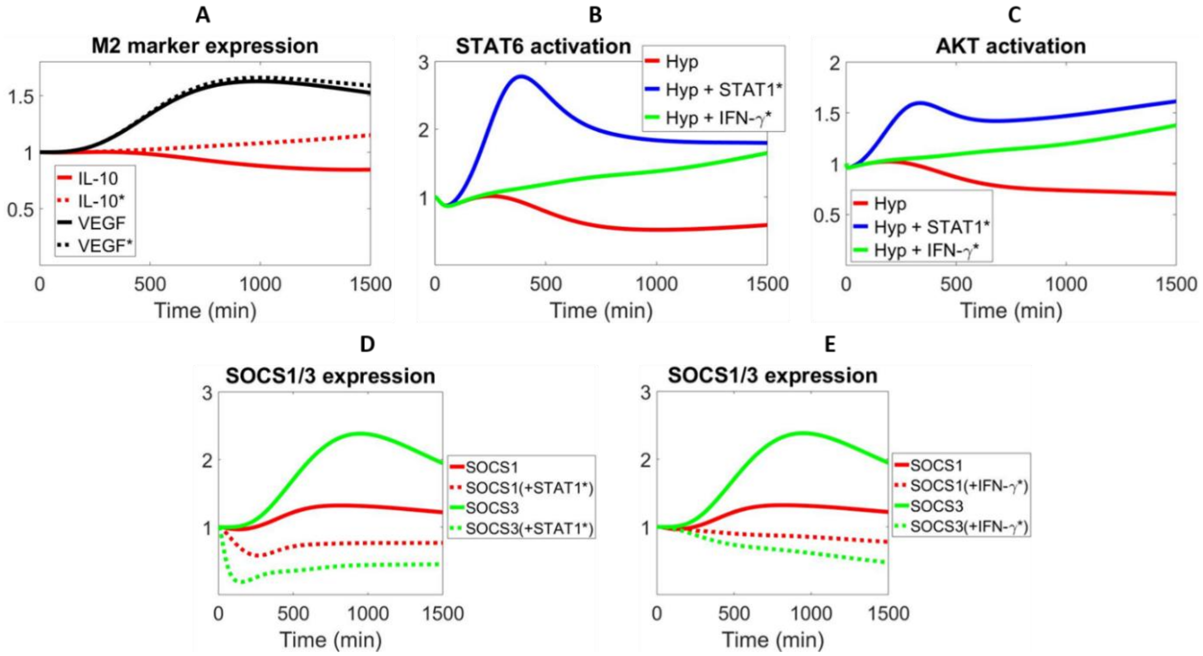

Figure S6. Temporal response of M2 markers and transcription factors under hypoxia. (A) Inhibition of IFN-γ production under hypoxia (dashed lines) can upregulation the expression of M2 markers IL-10 and VEGF (marginal effect), compared to hypoxia alone (solid lines). Simulations show stronger temporal activation of (B) pSTAT6 and (C) pAKT under hypoxia in combination with IFN-γ inhibition or STAT1 inhibition, compared to hypoxia alone. SOCS1 and SOCS3 expression are reduced under hypoxia with either (D) STAT1 or (E) IFN-γ inhibition (dashed lines), compared to hypoxia alone (solid lines). STAT1* means inhibition of STAT1 activation, IFN-γ* means inhibition of IFN-γ production. Inhibition of IFN-γ is simulated by setting its production rate to 10% of the original value; STAT1 inhibition is simulated as a 90% decrease in the binding rate between STAT1 and activated IFN-γ receptor complex. (A-E) Expression levels are normalized to their respective t=0 values (e.g. normoxia, unstimulated). Hyp – hypoxia. All simulation results are protein levels.
