# Supplementary material for: A mechanistic integrative computational model of macrophage polarization: Implications in human pathophysiology
Source: PLoS Comput Biol. 2019 Nov 18;15(11):e1007468. doi: 10.1371/journal.pcbi.1007468 (PMC6860420; doi:10.1371/journal.pcbi.1007468)
Supplement: S7 Fig — (A-E) Parameter sensitivities under high IL-4 production. (PDF) [file pcbi.1007468.s008.pdf]

Figure S7

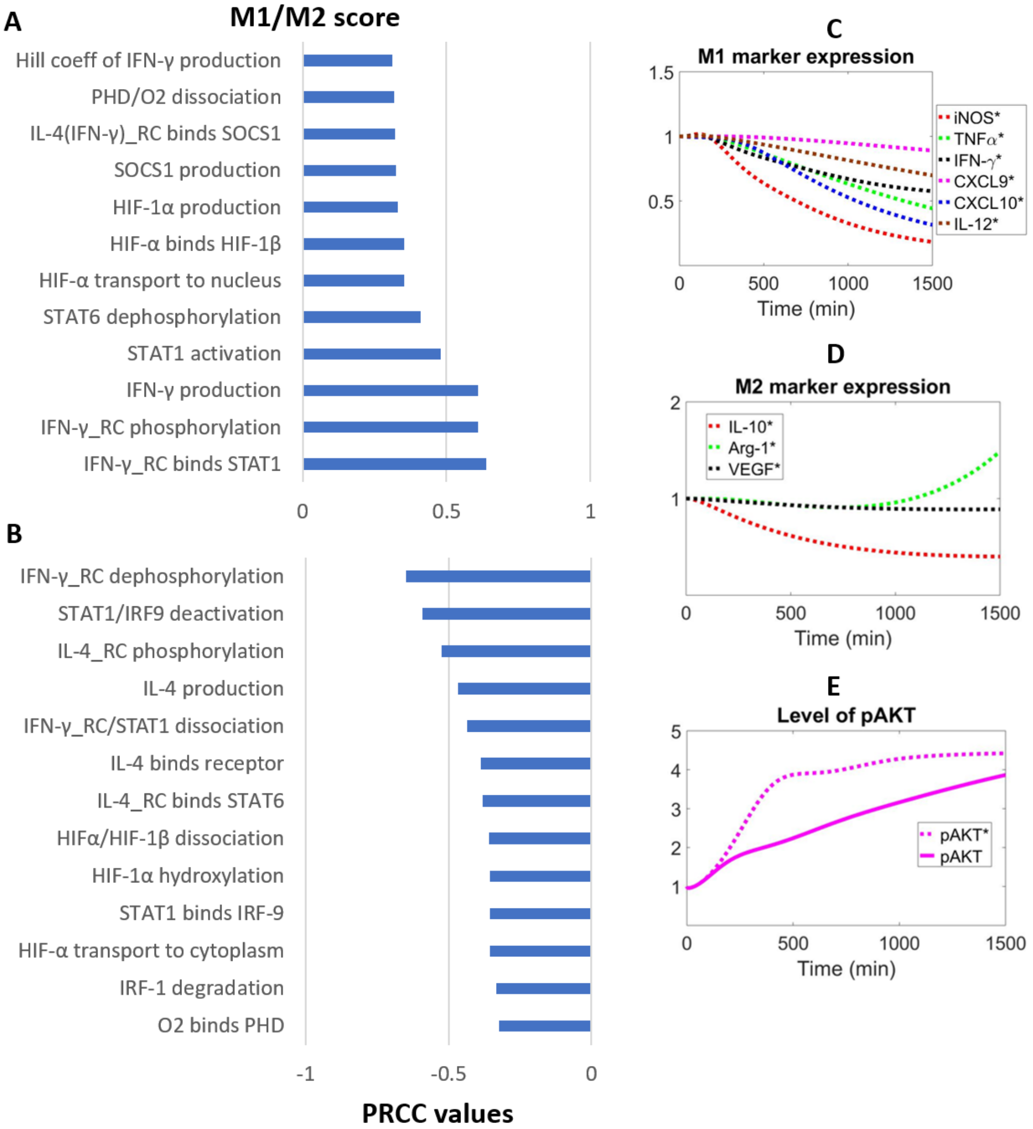

Figure S7. Parameter sensitivities under high IL-4 production. (A-B) Sensitivity indices (top 25 positive and negative PRCC values with $p<0.05$) of model parameters that control M1 and M2 marker expression in terms of the M1/M2 score under high IL-4 production (10x). In the parameter descriptions, 'X_RC' means receptor complex formed by ligand X, receptor and JAK, 'X/Y' means complex formed by X and Y. (C-D) Simulated relative time-course expression (dashed lines) of M1 and M2 markers when macrophages are subjected to AKT inhibition (simulated as a 90% decrease in the AKT activation rate) under high IL-4 production. (E) Under the scenario of high IL-4 production, inhibition of STAT6 triggers increased activation of AKT as a compensatory mechanism to further upregulate M2 marker expression. (C-E) Marker expression levels are normalized to their respective t=0 values (e.g. normal IL-4 production, unstimulated). All simulation results are protein levels (except CXCL10 is mRNA level). (A-B) More details about the parameters listed can be found in Table S1 using the labels (positive – *ka37, kr70, kf17, k127, k61, kf64, kf63, k33, k45, k37, kf42, kf44*; negative – *kr42, k99, kf8, k1, kr44, kf7, kf13, kr64, k71, kf95, kr63, k78, kf70*; order is from top to bottom as displayed).
