# Supplementary material for: A mechanistic integrative computational model of macrophage polarization: Implications in human pathophysiology
Source: PLoS Comput Biol. 2019 Nov 18;15(11):e1007468. doi: 10.1371/journal.pcbi.1007468 (PMC6860420; doi:10.1371/journal.pcbi.1007468)
Supplement: S8 Fig — (PDF) [file pcbi.1007468.s009.pdf]

Figure S8

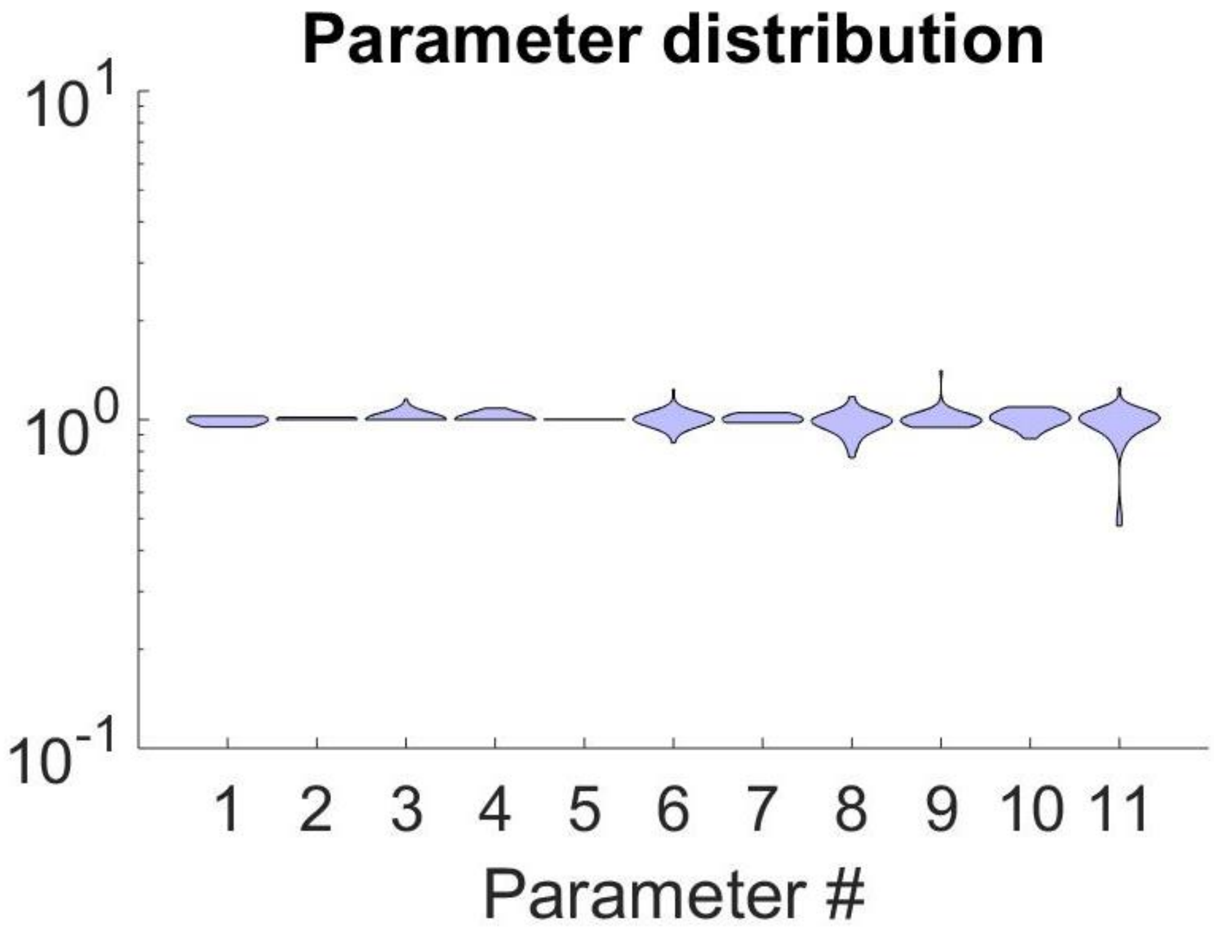

Figure S8. Parameter distribution after bootstrapping. Parameter estimate distributions (represented by violin plots) of the top 11 most sensitive parameters. The 11 parameters were re-estimated 50 times following the resampling procedures described in the Materials and Methods section (all parameter values are normalized to their respective original values for display, y-axis in log scale). During bootstrapping, parameter values are allowed to vary from 0.1x to 10x (of their original values). Parameter descriptions – #1. IFN-γ receptor dephosphorylation rate; 2. forward binding rate of activated IFN-γ receptor complex with STAT1; 3. deactivation rate of STAT1/IRF9 complex in nucleus; 4. IFN-γ receptor phosphorylation rate; 5. reverse binding (e.g. dissociation) rate of activated IFN-γ receptor complex with STAT1; 6. forward binding rate of IL-4 (or IFN-γ) receptor complex with SOCS1; 7. dephosphorylation rate of STAT6; 8. degradation rate of SOCS1/3-bound IL-4 (or IFN-γ) receptor complex; 9. rate of IL-4 receptor phosphorylation; 10. STAT1 activation rate; 11. forward binding rate of IFN-γ receptor complex with SOCS3. The 11 parameters are chosen based on the overall ranking of their absolute PRCC values (from high to low) derived from the sensitivity analysis in three scenarios (IL-4 stimulation, IFN-γ stimulation, and hypoxia).
