# Supplementary material for: A mechanistic integrative computational model of macrophage polarization: Implications in human pathophysiology
Source: PLoS Comput Biol. 2019 Nov 18;15(11):e1007468. doi: 10.1371/journal.pcbi.1007468 (PMC6860420; doi:10.1371/journal.pcbi.1007468)
Supplement: S1 Table — (PDF) [file pcbi.1007468.s010.pdf]

Table S1

No.	Reaction descriptions	Reaction flux details (v=)	Parameter values	Refs.
v1	IL-4 production	*k1*	*k1*=0.2 min^{-1}	Fitted
v2	IL-4R production	*k2*	*k2*=0.16 min^{-1}	Fitted
v3	IL-4R constitutive degradation	*k3**[IL4R]	*k3*=0.007 min^{-1}	Fitted
v4	JAK production	*k4*	*k4*=50 min^{-1}	Fitted
v5	JAK constitutive degradation	*k5**[JAK]	*k5*=0.0005 min^{-1}	(1)
v6	IL-4R pre-associates with JAK	*kf6**[JAK]*[IL4R]- *kr6**[IL4R/JAK]	*kf6*=4e-6 min^{-1}, *kr6*=0.0018 min^{-1}	Fitted
v7	IL-4 binds receptor	*kf7**[IL4]*[IL4R/JAK]- *kr7**[IL4/R/JAK]	*kf7*=2e-7 min^{-1}, *kr7*=0.01 min^{-1}	(2)
v8	IL-4 receptor complex phosphorylation	*kf8**[IL4/R/JAK]- *kr8**[pIL4/R/JAK]	*kf8*=0.6 min^{-1}, *kr8*=0.1 min^{-1}	Fitted
v9	Internalization of phosphorylated IL-4 receptor complex	*k9**[pIL4/R/JAK]	*k9*=0.2 min^{-1}	Fitted
v10	Shuttling of IL-4 receptor complex to lysosomes	*k10**[pIL4/R/JAK_i]	*k10*=1 min^{-1}	Fitted
v11	Degradation of IL-4 and IL-4R in lysosomes	*k11**[IL4/R_lyso]	*k11*=0.3 min^{-1}	Fitted
v12	Recycling of IL-4R	*k12**[IL4/R_lyso]	*k12*=1 min^{-1}	Fitted
v13	Binding of STAT6 with ligand-activated IL-4 receptor complex	*kf13**[STAT6]*[pIL4/R/JAK _i]- *kr13**[pIL4/R/JAK/STAT6]	*kf13*=0.1 min^{-1}, *kr13*=10 min^{-1}	Fitted
v14	Activation of STAT6 by phosphorylation	*k14**[pIL4/R/JAK/STAT6]	*k14*=8 min^{-1}	Fitted
v15	Phosphorylation of AKT	*k15**[AKT]*(1-[PTEN]/([PTEN]+ka15))*([pIL4/R/JAK]/([pIL4/R/JAK]+kb15))	*k15*=1.16 min^{-1}, *ka15*=5000, *kb15*=4	Fitted
v16	AKT dephosphorylation	*k16**[pAKT]	*k16*=0.015 min^{-1}	(3)
v17	Internalized IL-4 receptor complex binds SOCS1	*kf17**[pIL4/R/JAK_i]*[SOCS1]- *kr17**[IL4/R/JAK/SOCS1_i]	*kf17*=0.008 min^{-1}, *kr17*=0.2 min^{-1}	Fitted
v18	SOCS1 inhibits IL-4 signaling and shuttles IL-4 and receptor to lysosomes	*k10**[IL4/R/JAK/SOCS1_i]		*
v19	SOCS1 sequesters JAK from the IL-4 receptor complex	*k19**[IL4/R/JAK/SOCS1_i]	*k19*=0.03 min^{-1}	Fitted
v20	Shuttling of internalized IL-4 and receptor to lysosomes	*k10**[IL4/R]		*
v21	SOCS1 targets JAK for degradation	*k21**[SOCS1/JAK]	*k21*=0.1 min^{-1}	Fitted
v22	Internalized IL-4 and receptor associate with JAK to reactivate signaling	*kf6**[IL4/R]*[JAK]- *kr6**[pIL4/R/JAK_i]		*
v23	Internalized IL-4 receptor complex binds SOCS3	*kf23**[pIL4/R/JAK_i]*[SOCS3]- *kr17**[IL4/R/JAK/SOCS3_i],	*kf23*=0.0004 min^{-1}	Fitted *

v24	SOCS3 inhibits IL-4 signaling and shuttles IL-4 and receptor to lysosomes	*k10**[IL4/R/JAK/SOCS3_i]		*
v25	SOCS3 sequesters JAK from the IL-4 receptor complex	*k19**[IL4/R/JAK/SOCS3_i]		*
v26	SOCS3 targets JAK for degradation	*k26**[SOCS3/JAK]	*k26*=0.01 min^{-1}	Fitted
v27	Surface IL-4 receptor complex binds SOCS1	*kf17**[IL4/R/JAK]*[SOCS1]-*kr17**[IL4/R/JAK/SOCS1]		*
v28	SOCS1-mediated shuttling of surface IL-4 and receptor to lysosomes	*k28**[IL4/R/JAK/SOCS1]	*k28*=0.1 min^{-1}	Fitted
v29	Surface IL-4 receptor complex binds SOCS3	*kf23**[IL4/R/JAK]*[SOCS3]-*kr17**[IL4/R/JAK/SOCS3]		*
v30	SOCS3-mediated shuttling of surface IL-4 and receptor to lysosomes	*k28**[IL4/R/JAK/SOCS3]		*
v31	pSTAT6 dimerization	*kf31**[pSTAT6]*[pSTAT6]-*kr31**[pSTAT6D]	*kf31*=0.002 min^{-1}, *kr31*=1 min^{-1}	Fitted
v32	Activated STAT6 dimer transports to nucleus	*k32**[pSTAT6D]	*k32*=0.4 min^{-1}	Fitted
v33	Dephosphorylation of nuclear STAT6 dimer	*k33**[pSTAT6D_n]	*k33*=0.01 min^{-1}	(4)
v34	Dissociation of STAT6 dimer in nucleus	*kr31**[STAT6D_n]		*
v35	Nuclear export of STAT6	*k35**[STAT6_n]	*k35*=0.05 min^{-1}	(5)
v36	Dephosphorylation of STAT6	*k33**[pSTAT6]		*
v37	IFN-γ production	*k37**([HIF1α/β_n]+0.2*[HIF2α/β_n])*(1-[pSTAT6D_n]/([pSTAT6D_n]+*ka37*))	*k37*=0.0288 min^{-1}, *ka37*=100	Fitted
v38	IFNGR production	*k38*	*k38*=1.22 min^{-1}	Fitted
v39	IFNGR constitutive degradation	*k39**[IFNGR]	*k39*=0.006 min^{-1}	(6)
v40	IFNGR pre-associates with JAK	*kf6**[IFNGR]*[JAK]-*kr40**[IFNGR/JAK]	*kr40*=0.002 min^{-1}	Fitted *
v41	IFN-γ binds receptor	*kf41**[IFNγ]*[IFNGR/JAK]-*kr7**[IFNγ/R/JAK]	*kf41*=1.6e-7 min^{-1}	(7, 8)*
v42	IFN-γ receptor complex phosphorylation	*kf42**[IFNγ/R/JAK]-kr42*[pIFNγ/R/JAK]	*kf42*=3 min^{-1}, k*r42*=10 min^{-1}	Fitted
v43	IFN-γ receptor complex binds SOCS1	*kf17**[IFNγ/R/JAK]*[SOCS1]-*kr17**[IFNγ/R/JAK/SOCS1]		*
v44	STAT1 binds ligand-activated IFN-γ receptor complex	*kf44**[STAT1]*[pIFNγ/R/JAK]-*kr44**[pIFNγ/R/JAK/STAT1]	*kf44*=0.03 min^{-1}, *kr44*=3 min^{-1}	Fitted
v45	STAT1 activation by phosphorylation	*k45**[pIFNγ/R/JAK/STAT1]	*k45*=2 min^{-1}	Fitted
v46	Shuttling of IFN-γ receptor complex to lysosomes	*k46**[pIFNγ/R/JAK]	*k46*=0.1 min^{-1}	Fitted

v47	Degradation of IFN-γ and IFNGR in lysosomes	k47*[IFNγ/R_lyso]	k47=1 min^{-1}	Fitted
v48	Recycling of IFNGR	k12*[IFNγ/R_lyso]		*
v49	SOCS1 inhibits IFN-γ signaling and shuttles IFN-γ and receptor to lysosomes	k46*[IFNγ/R/JAK/SOCS1]		*
v50	SOCS1 sequesters JAK from the IFN-γ receptor complex	k19*[IFNγ/R/JAK/SOCS1]		*
v51	IFN-γ and receptor bind JAK to reactivate signaling	kf6*[IFNγ/R]*[JAK]-kr40*[IFNγ/R/JAK],		*
v52	IFN-γ receptor complex binds SOCS3	kf52*[IFNγ/R/JAK]*[SOCS3]-kr17*[IFNγ/R/JAK/SOCS3]	kf52=0.004 min^{-1}	Fitted *
v53	SOCS3 inhibits IFN-γ signaling and shuttles IFN-γ and receptor to lysosomes	k46*[IFNγ/R/JAK/SOCS3]		*
v54	SOCS3 sequesters JAK from the IFN-γ receptor complex	k19*[IFNγ/R/JAK/SOCS3]		*
v55	pSTAT1 dimerization	kf55*[pSTAT1]*[pSTAT1]-kr31*[pSTAT1D]	kf55=0.1 min^{-1}	Fitted *
v56	Activated STAT1 dimer transports to nucleus	k56*[pSTAT1D]	k56=1 min^{-1}	Fitted
v57	Dephosphorylation of nuclear STAT1 dimer	k57*[pSTAT1D_n]	k57=0.03 min^{-1}	(9)
v58	Dissociation of STAT1 dimer in nucleus	kr31*[STAT1D_n]		*
v59	Nuclear export of STAT1	k59*[STAT1_n]	k59=0.1 min^{-1}	Fitted
v60	Dephosphorylation of STAT1	k57*[pSTAT1]		*
v61	HIF-1α production is promoted by TNFα signaling and downregulated by miR-93	k61*([TNFα]+ka61)*(1.4-[miR93]/([miR93]+kb61))	k61=0.0187 min^{-1}, ka61=800, kb61=1000	Fitted
v62	HIF-2α production is promoted by PPARγ	k62*([aPPARG]/([aPPARG]+ka62))	k62=178 min^{-1}, ka62=10000	Fitted
v63	HIF-1α transport to nucleus	kf63*[HIF1α]-kr63*[HIF1α_n]	kf63=0.005 min^{-1}, kr63=0.018 min^{-1}	(10)
v64	HIF-2α transport to nucleus	kf63*[HIF2α]-kr63*[HIF2α_n]		*
v65	Nuclear HIF-1α binds HIF-1β	kf64*[HIF1α_n]*[HIF1β_n]-kr64*[HIF1α/β_n]	kf64=5e-7 min^{-1}, kr64=0.03 min^{-1}	Fitted; (10)
v66	Nuclear HIF-2α binds HIF-1β	kf64*[HIF2α_n]*[HIF1β_n]-kr64*[HIF2α/β_n]		*
v67	PHD production	k67*(0.0001+[HIF1α/β_n]^2/([HIF1α/β_n]^2+ka67)*[HIF2α/β_n]^2/([HIF2α/β_n]^2+kb67))	k67=602 min^{-1}, ka67=32600, kb67=25400	Fitted
v68	PHD degradation	k68*[PHD]	k68=0.0008 min^{-1}	(1)
v69	Itaconate influences PHD activation	kf69*[PHD]*[Itaconate]-kr69*[aPHD]	kf69=8e-9 min^{-1}, kr69=0.07 min^{-1}	Fitted
v70	PHD binds oxygen	kf70*[aPHD]*[O_2]-kr70*[O_2/aPHD]	kf70=7.14e-9 min^{-1}, kr70=10.8 min^{-1}	Fitted; (10)
v71	HIF1α hydroxylation	k71*[O_2/aPHD]*[HIF-1α]	k71=1.33e-5 min^{-1}	Fitted

v72	HIF1α de-ubiquitination	*k72**[HIF1α_OH]	*k72*=0.03 min^{-1}	Fitted
v73	Degradation of hydroxylated HIF-1α	*k73**[HIF1α_OH]	*k73*=0.6 min^{-1}	Fitted
v74	HIF2α hydroxylation	*k74**[O_2/aPHD]*[HIF-2α]	*k74*=5e-6 min^{-1}	Fitted
v75	HIF2α de-ubiquitination	*k72**[HIF2α_OH]		*
v76	Degradation of hydroxylated HIF-2α	*k73**[HIF2α_OH]		*
v77	STAT1 and STAT6 regulate IRF-1 production	*k77**(([pSTAT1D_n]+[pSTAT1D/IRF9_n])/[pSTAT6D_n])/(([pSTAT1D_n]+[pSTAT1D/IRF9_n])/[pSTAT6D_n]+*ka77*)	*k77*=106 min^{-1}, *ka77*=50	Fitted
v78	IRF-1 degradation	*k78**[IRF1]	*k78*=0.022 min^{-1}	(11)
v79	IFN-γ (through IRF-1) inhibits miR-3473 production	*k79**(1-$[IRF1]^2$/($[IRF1]^2$+*ka79*))	*k79*=14.8 min^{-1}, *ka79*=40000	Fitted
v80	Degradation of miR-3473	*k80**[miR3473]	*k80*=0.0012 min^{-1}	(12)
v81	miR-3473 binds PTEN mRNA	*kf81**[miR3473]*[mPTEN]-*kr81**[miR3473/mPTEN]	*kf81*=0.0001 min^{-1}, *kr81*=0.06 min^{-1}	(13)
v82	Degradation of miR3473-bound PTEN mRNA	*k82**[miR3473/mPTEN]	*k82*=0.1 min^{-1}	Fitted
v83	Production of PTEN mRNA	*k83*	*k83*=0.64 min^{-1}	Fitted
v84	PTEN translation	*k84**[mPTEN]	*k84*=2.32 min^{-1}	Fitted
v85	PTEN degradation	*k85**[PTEN]	*k85*=0.0003 min^{-1}	(1)
v86	PTEN mRNA constitutive degradation	*k86**[mPTEN]	*k86*=0.003 min^{-1}	(14)
v87	miR-93 production is downregulated by IFN-γ (represented by STAT1) and TNFα signaling	*k87**(1-[TNFα]*$[pSTAT1D\_n]^2$/([TNFα] *$[pSTAT1D\_n]^2$+*ka87*))	*k87*=6050 min^{-1}, *ka87*=1000	Fitted
v88	miR-93 degradation	*k88**[miR93]	*k88*=0.0018 min^{-1}	(12)
v89	miR-93 binds IRF9 mRNA	*kf81**[miR93]*[mIRF9]-*kr*81*[miR93/mIRF9]		*
v90	Degradation of miR93-bound IRF9 mRNA	*k82**[miR93/mIRF9]		*
v91	IRF9 mRNA production	*k91*	*k91*=0.5 min^{-1}	Fitted
v92	IRF9 mRNA constitutive degradation	*k86**[mIRF9]		*
v93	IRF9 translation	*k93**[mIRF9]	*k93*=1.18 min^{-1}	Fitted
v94	IRF9 degradation	*k94**[IRF9]	*k94*=0.004 min^{-1}	Fitted
v95	IRF9 binds activated STAT1 dimer in cytoplasm	*kf95**[IRF9]*[pSTAT1D]-*kr95**[pSTAT1D/IRF9]	*kf95*=0.00013 min^{-1}, *kr95*=1 min^{-1}	Fitted
v96	Nuclear translocation of STAT1/IRF9 complex	*k96**[pSTAT1D/IRF9]	*k96*=0.1 min^{-1}	Fitted
v97	IRF9 binds activated STAT1 dimer in nucleus	*kf95**[IRF9_n]*[pSTAT1D_n]-*kr95**[pSTAT1D/IRF9_n]		*
v98	IRF9 translocation to nucleus	*k96**[IRF9]-*kr98**[IRF9_n]	*kr98*=0.01 min^{-1}	Fitted
v99	Deactivation of STAT1/IRF9 complex in nucleus	*k99**[pSTAT1D/IRF9_n]	*k99*=0.4 min^{-1}	Fitted

v100	IRF4 production regulated by STAT6 and AKT	*k100**[pSTAT6D_n]/([pSTAT6D_n]+*ka100*)*[pAKT]/([pAKT]+*kb100*)	*k100*=6840 min^{-1}, *ka100*=30000, *kb100*=20000	Fitted
v101	IRF4 degradation	*k101**[IRF4]	*k101*=0.0002 min^{-1}	(1)
v102	STAT6 promotes PPARγ production	*k102**[pSTAT6D_n]/([pSTAT6D_n]+*ka102*)	*k102*=107 min^{-1}, *ka102*=1000	Fitted
v103	AKT promotes PPARγ activation	*k103**[PPARG]*[pAKT]/([pAKT]+*ka103*)	*k103*=50 min^{-1}, *ka103*=10000	Fitted
v104	PPARγ deactivation	*k104**[aPPARG]	*k104*=50 min^{-1}	Fitted
v105	Degradation of activated PPARγ	*k105**[aPPARG]	*k105*=0.0018 min^{-1}	(15)
v106	Degradation of PPARγ	*k105**[PPARG]		*
v107	IRF9 and IRF1 promotes IRG-1 production	*k107**[IRF1]*([IRF9_n]+[pSTAT1D/IRF9_n])^2	*k107*=4.77e-12 min^{-1}	Fitted
v108	IRG1 degradation	*k108**[IRG1]	*k108*=6.2e-4 min^{-1}	(1)
v109	IRG1 promotes itaconate production	*k109**[IRG1]^2	*k109*=0.025 min^{-1}	Fitted
v110	Itaconate degradation	*k110**[Itaconate]	*k110*=0.005 min^{-1}	Fitted
v111	HIF1/2 and AKT induce VEGF production	*k111**([HIF1α/β_n]*[HIF2α/β_n]+*ka111*)*([pAKT]+*kb111*)	*k111*=3e-9 min^{-1}, *ka111*=29200, *kb111*=42700	Fitted
v112	Removal of secreted VEGF	*k112**[VEGF]	*k112*=0.001 min^{-1}	Fitted
v113	iNOS production is dependent on HIF1, IRF1 and type I IFN signaling (represented by IRF9)	*k113**(0.02+[HIF1α/β_n]/([HIF1α/β_n]+*ka113*))*([IRF1]/([IRF1]+*kb113*))*([IRF9T]+15000)	*k113*=0.036 min^{-1}, *ka113*=4000, *kb113*=9000	Fitted
v114	iNOS degradation	*k114**[iNOS]	*k114*=0.006 min^{-1}	(16)
v115	ARG1 production is promoted by STAT6, IRF4 and HIFs while downregulated by TNFα	*k115**([pSTAT6D_n]*[IRF4]+*ka115*)*([HIF1α/β_n]+2*[HIF2α/β_n])^2*(1.2-[TNFα]^2/([TNFα]^2+*kb115*))	*k115*=3.76e-9 min^{-1}, *ka115*=1.2e7, *kb115*=100000	Fitted
v116	ARG1 degradation	*k116**[ARG1]	*k116*=0.0006 min^{-1}	(17, 18)
v117	IRF1 and type I IFNs (represented by IRF9) can induce TNFα production	*k117** [IRF1]^2/([IRF1]^2+*ka117*)*(30000+[IRF9T])^2	*k117*=7.2e-10 min^{-1}, *ka117*=1.6e6	Fitted
v118	Removal of secreted TNFα	*k118**[TNFα]	*k118*=0.0008 min^{-1}	Fitted
v119	IL-10 production is promoted by AKT, type I IFNs (represented by IRF9) and STAT3 (represented by secreted IL-10)	*k119**[pAKT]*(2000+[IL10])*([IRF9T]+15000)	*k119*=6.22e-13 min^{-1}	Fitted
v120	Removal of secreted IL-10	*k120**[IL10]	*k120*=0.0015 min^{-1}	Fitted
v121	IRF1 promotes IL-12 production	*k121**[IRF1]^2	*k121*=5e-8 min^{-1}	Fitted
v122	Removal of secreted IL-12	*k122**[IL12]	*k122*=0.00033 min^{-1}	Fitted
v123	STAT1 induces CXCL9 synthesis	*k123**[pSTAT1D_n]^2	*k123*=1.67e-6 min^{-1}	Fitted
v124	Removal of secreted CXCL9	*k124**[CXCL9]	*k124*=0.0002 min^{-1}	Fitted

v125	STAT1/IRF9 complex induces CXCL10 mRNA production	*k125**[pSTAT1D/IRF9_n]^2	*k125*=1e-6 min^{-1}	Fitted
v126	CXCL10 mRNA degradation	*k126**[mCXCL10]	*k126*=0.0035 min^{-1}	(14)
v127	STAT1 and STAT6 induce SOCS1 production	*k127**[pSTAT1D_n]/([pSTAT1D_n]+*ka127*)*[pSTAT6D_n]/([pSTAT6D_n]+*kb127*)	*k127*=13342 min^{-1}, *ka127*=6000, *kb127*=500	Fitted
v128	Degradation of SOCS1	*k128**[SOCS1]	*k128*=0.004 min^{-1}	(19)
v129	STAT1 induces SOCS3 production	*k129**[pSTAT1D_n]/([pSTAT1D_n]+*ka129*)	*k129*=6571 min^{-1}, *ka129*=6000	Fitted
v130	Degradation of SOCS3	*k130**[SOCS3]	*k130*=0.05 min^{-1}	Fitted

Table S1. Complete list of model reactions and parameter values. Reactions are formulated mechanistically based on literature evidence (the labels *v#* here match with the labels in Figure S1 and Table S2). As shown in the last column, numerical values of model parameters (~140 in total) are either estimated from relevant published data (e.g. experimental measurements, prior models) or estimated computationally through whole-model optimization (‘fitted’). Certain parameter values are shared by more than one reaction fluxes (marked by * in the last column).

References

1. Cambridge SB, Gnad F, Nguyen C, Bermejo JL, Kruger M, Mann M. Systems-wide proteomic analysis in mammalian cells reveals conserved, functional protein turnover. J Proteome Res. 2011;10(12):5275-84.
2. Bankaitis KV, Fingleton B. Targeting IL4/IL4R for the treatment of epithelial cancer metastasis. Clin Exp Metastasis. 2015;32(8):847-56.
3. Gao T, Furnari F, Newton AC. PHLPP: a phosphatase that directly dephosphorylates Akt, promotes apoptosis, and suppresses tumor growth. Mol Cell. 2005;18(1):13-24.
4. Hanson EM, Dickensheets H, Qu CK, Donnelly RP, Keegan AD. Regulation of the dephosphorylation of Stat6. Participation of Tyr-713 in the interleukin-4 receptor alpha, the tyrosine phosphatase SHP-1, and the proteasome. J Biol Chem. 2003;278(6):3903-11.
5. Chen HC, Reich NC. Live cell imaging reveals continuous STAT6 nuclear trafficking. J Immunol. 2010;185(1):64-70.
6. Londino JD, Gulick DL, Lear TB, Suber TL, Weathington NM, Masa LS, et al. Post-translational modification of the interferon-gamma receptor alters its stability and signaling. Biochem J. 2017;474(20):3543-57.
7. Ping Z, Qi J, Sun Y, Lu G, Shi Y, Wang X, et al. Crystal structure of the interferon gamma receptor alpha chain from chicken reveals an undetected extra helix compared with the human counterparts. J Interferon Cytokine Res. 2014;34(1):41-51.
8. Nickoloff BJ. Binding of 125I-gamma interferon to cultured human keratinocytes. J Invest Dermatol. 1987;89(2):132-5.
9. Zhu W, Mustelin T, David M. Arginine methylation of STAT1 regulates its dephosphorylation by T cell protein tyrosine phosphatase. J Biol Chem. 2002;277(39):35787-90.

10. Zhao C, Isenberg JS, Popel AS. Transcriptional and Post-Transcriptional Regulation of Thrombospondin-1 Expression: A Computational Model. PLoS Comput Biol. 2017;13(1):e1005272.
11. Eckhardt I, Weigert A, Fulda S. Identification of IRF1 as critical dual regulator of Smac mimetic-induced apoptosis and inflammatory cytokine response. Cell Death Dis. 2014;5:e1562.
12. Marzi MJ, Ghini F, Cerruti B, de Pretis S, Bonetti P, Giacomelli C, et al. Degradation dynamics of microRNAs revealed by a novel pulse-chase approach. Genome Res. 2016;26(4):554-65.
13. Wang X, Li Y, Xu X, Wang YH. Toward a system-level understanding of microRNA pathway via mathematical modeling. Biosystems. 2010;100(1):31-8.
14. Sharova LV, Sharov AA, Nedorezov T, Piao Y, Shaik N, Ko MS. Database for mRNA half-life of 19 977 genes obtained by DNA microarray analysis of pluripotent and differentiating mouse embryonic stem cells. DNA Res. 2009;16(1):45-58.
15. Waite KJ, Floyd ZE, Arbour-Reily P, Stephens JM. Interferon-gamma-induced regulation of peroxisome proliferator-activated receptor gamma and STATs in adipocytes. J Biol Chem. 2001;276(10):7062-8.
16. Kolodziejski PJ, Koo JS, Eissa NT. Regulation of inducible nitric oxide synthase by rapid cellular turnover and cotranslational down-regulation by dimerization inhibitors. Proc Natl Acad Sci U S A. 2004;101(52):18141-6.
17. Setty BA, Jin Y, Houghton PJ, Yeager ND, Gross TG, Nelin LD. Hypoxic Proliferation of Osteosarcoma Cells Depends on Arginase II. Cell Physiol Biochem. 2016;39(2):802-13.
18. Schimke RT. The Importance of Both Synthesis and Degradation in the Control of Arginase Levels in Rat Liver. J Biol Chem. 1964;239:3808-17.
19. Zimmer J, Weitnauer M, Boutin S, Kublbeck G, Thiele S, Walker P, et al. Nuclear Localization of Suppressor of Cytokine Signaling-1 Regulates Local Immunity in the Lung. Front Immunol. 2016;7:514.
