# Supplementary material for: A mechanistic integrative computational model of macrophage polarization: Implications in human pathophysiology
Source: PLoS Comput Biol. 2019 Nov 18;15(11):e1007468. doi: 10.1371/journal.pcbi.1007468 (PMC6860420; doi:10.1371/journal.pcbi.1007468)
Supplement: S2 Table — (PDF) [file pcbi.1007468.s011.pdf]

Table S2

Name of model node	Initial condition (in # of molecules)	Equation (d[x]/dt=)	Initial condition Refs.
VEGF	5223	*v111-v112*	Estimated from (1)
IFNγ	358	*v37-v41*	Estimated from (2)
IFNGR/JAK	20376	*v40-v41*	Total IFNGR estimated from (3, 4)
IFNGR	106	*-v40+v48*	Fitted
IFNγ/R	1	*v50+v54-v51*	Fitted
IFNγ/R/JAK	0	*v41-v42-v43+v51-v52*	Fitted
pIFNγ/R/JAK	0	*v42-v44*	Fitted
STAT1	397262	*-v44+v59+v60*	Total STAT1 estimated from (4, 5)
pIFNγ/R/JAK/STAT1	84	*v44-v45*	Fitted
pSTAT1	41	*v45-v60-2*v55*	Fitted
pSTAT1D	81	*v55-v56-v95*	Fitted
pSTAT1D_n	80	*v56-v57-v97*	Fitted
STAT1D_n	84	*v57+v99-v58*	Fitted
STAT1_n	1673	*2*v58-v59*	Fitted
SOCS1	3169	*v127-v128-v17+v18+v21-v27+v28-v43+v49*	Total SOCS1 estimated from (6)
IFNγ/R/JAK/SOCS1	9	*v43-v49-v50*	Fitted
IFNγ/R_lyso	1	*v46-v47-v48+v49+v53*	Fitted
SOCS1/JAK	3	*v50+v19-v21*	Fitted
IRF9	2674	*v93-v94-v95-v98*	Total IRF9 estimated from (4)
IRF9_n	27001	*v98-v97+v99*	Fitted
pSTAT1D/IRF9	26	*v95-v96*	Fitted
pSTAT1D/IRF9_n	203	*v96-v99+v97*	Fitted
SOCS3	1735	*v129-v130-v23+v24+v26-v29+v30-v52+v53*	Total SOCS3 estimated from (6)
IFNγ/R/JAK/SOCS3	3	*v52-v53-v54*	Fitted
SOCS3/JAK	8	*v54+v25-v26*	Fitted
IL4	288	*v1-v7*	Estimated from (7)
IL4R	16	*v2-v6+v12*	Fitted
IL4/R	0	*v19+v25-v22*	Fitted
IL4R/JAK	3478	*v6-v7*	Total IL4R estimated from (4)
IL4/R/JAK	0	*v7-v8*	Fitted
pIL4/R/JAK	0	*v8-v9*	Fitted
pIL4/R/JAK_i	0	*v9-v10-v13+v14-v17+v22-v23*	Fitted
IL4/R/JAK/SOCS1_i	0	*v17-v18-v19*	Fitted
IL4/R_lyso	0	*v10-v11-v12+v18+v20+v24+v28+v30*	Fitted

IL4/R/JAK/SOCS3_i	0	*v23-v24-v25*	Fitted
IL4/R/JAK/SOCS1	2	*v27-v28*	Fitted
IL4/R/JAK/SOCS3	0	*v29-v30*	Fitted
STAT6	59874	*v35+v36-v13*	Total STAT6 estimated from (4, 6)
pIL4/R/JAK/STAT6	0	*v13-v14*	Fitted
pSTAT6	26	*v14-v36-2*v31*	Fitted
pSTAT6D	1	*v31-v32*	Fitted
pSTAT6D_n	39	*v32-v33*	Fitted
STAT6D_n	0	*v33-v34*	Fitted
STAT6_n	16	*2*v34-v35*	Fitted
PTEN	64899	*v84-v85*	Estimated from (4)
IRF4	16460	*v100-v101*	Estimated from (4)
IL10	571	*v119-v120*	Estimated from (8)
JAK	99297	*v4-v5-v6+v10+v18-v22+v24+v28+v30-v40+v46+v49+v53*	Total JAK estimated from (4)
ARG1	875428	*v115-v116*	Estimated from (4)
miR3473	1180	*v79-v80-v81+v82*	Estimated from (9)
mPTEN	8	*v83-v86-v81*	Total mPTEN estimated from (10)
miR3473/mPTEN	6	*v81-v82*	Fitted
iNOS	538	*v113-v114*	Fitted
HIF1α	640	*v61-v63-v71+v72*	Total HIF1α estimated from (11)
HIF1α_n	178	*v63-v65*	Fitted
HIF1α/β_n	44	*v65*	Fitted
HIF1α_OH	42	*v71-v72-v73*	Fitted
HIF2α	878	*v62-v64-v74+v75*	Total HIF2α estimated from (11)
HIF2α_n	244	*v64-v66*	Fitted
HIF2α/β_n	61	*v66*	Fitted
HIF2α_OH	22	*v74-v75-v76*	Fitted
HIF1β_n	14897	*-v65-v66*	Total HIF1β estimated from (4)
PPARG	1437	*v102-v103+v104-v106*	Total PPARG estimated from (12)
aPPARG	782	*v103-v104-v105*	Fitted
IRF1	614	*v77-v78*	Estimated from (4)
PHD	5470	*v67-v68-v69*	Total PHD estimated from (4)
O_2	1.204e8	*Constant*	Estimated from (11)
O_2/aPHD	3088	*v70-v71-v74*	Fitted
aPHD	38846	*v69-v70+v71+v74*	Fitted
TNFα	619	*v117-v118*	Estimated from (13-15)
CXCL9	54	*v123-v124*	Fitted

mIRF9	9	*v91-v92-v89*	Total mIRF9 estimated from (16)
miR93/mIRF9	5	*v89-v90*	Fitted
miR93	841	*v87-v88-v89+v90*	Fitted
IL12	58	*v121-v122*	Estimated from (17)
mCXCL10	12	*v125-v126*	Estimated from (16)
AKT	198060	*-v15+v16*	Total AKT estimated from (4)
pAKT	11942	*v15-v16*	Fitted
IRG1	3522	*v107-v108*	Estimated from (4)
Itaconate	6.214e7	*v109-v110*	Estimated from (18)
IRF9T (repeated assignment)	29904	*[IRF9T]=[IRF9]+[IRF9_n]+ [pSTAT1D/IRF9]+[pSTAT1D/IRF9_n]*	Fitted

Table S2. Differential equations and initial conditions of all model nodes. A summary of the model equations and initial conditions in terms of absolute copy numbers of all 80 model nodes (proteins, RNAs, complexes, etc.) derived from the 34 functionally unique species. The initial levels (and also the steady state levels) of 31 out of 34 unique species are estimated and calibrated with respect to literature data (using both direct measurements and indirect observations), and the model is simulated in control condition without external stimulation until equilibrium to obtain the initial levels (e.g. copy numbers) of all 80 model nodes. A cell volume of 1 pL is assumed when doing unit conversion calculations for O_2 and itaconate (19, 20). To reduce model complexity, it is assumed that transcription factors and enzymes in Hill-type reactions are not consumed, mRNAs are not consumed during translation, and oxygen level is constant during each simulation run.

References

1. Eubank TD, Roda JM, Liu H, O'Neil T, Marsh CB. Opposing roles for HIF-1alpha and HIF-2alpha in the regulation of angiogenesis by mononuclear phagocytes. Blood. 2011;117(1):323-32.
2. Yu Y, Cui Y, Zhao Y, Liu S, Song G, Jiao P, et al. The binding capability of plasma phospholipid transfer protein, but not HDL pool size, is critical to repress LPS induced inflammation. Sci Rep. 2016;6:20845.
3. Crouse JA, Mitchell WM. Interferon-gamma receptor: mRNA half-life, receptor mass, and abundance on A431 human epidermoid carcinoma cells. J Interferon Res. 1992;12(1):23-5.
4. Azimifar SB, Nagaraj N, Cox J, Mann M. Cell-type-resolved quantitative proteomics of murine liver. Cell Metab. 2014;20(6):1076-87.
5. Precious BL, Carlos TS, Goodbourn S, Randall RE. Catalytic turnover of STAT1 allows PIV5 to dismantle the interferon-induced anti-viral state of cells. Virology. 2007;368(1):114-21.
6. Brenes A, Afzal V, Kent R, Lamond AI. The Encyclopedia of Proteome Dynamics: a big data ecosystem for (prote)omics. Nucleic Acids Res. 2018;46(D1):D1202-D9.

7. Mukherjee S, Chen LY, Papadimos TJ, Huang S, Zuraw BL, Pan ZK. Lipopolysaccharide-driven Th2 cytokine production in macrophages is regulated by both MyD88 and TRAM. J Biol Chem. 2009;284(43):29391-8.
8. Yang HL, Zhou WJ, Chang KK, Mei J, Huang LQ, Wang MY, et al. The crosstalk between endometrial stromal cells and macrophages impairs cytotoxicity of NK cells in endometriosis by secreting IL-10 and TGF-beta. Reproduction. 2017;154(6):815-25.
9. Bissels U, Wild S, Tomiuk S, Holste A, Hafner M, Tuschl T, et al. Absolute quantification of microRNAs by using a universal reference. RNA. 2009;15(12):2375-84.
10. Schwanhausser B, Busse D, Li N, Dittmar G, Schuchhardt J, Wolf J, et al. Global quantification of mammalian gene expression control. Nature. 2011;473(7347):337-42.
11. Tuckerman JR, Zhao Y, Hewitson KS, Tian YM, Pugh CW, Ratcliffe PJ, et al. Determination and comparison of specific activity of the HIF-prolyl hydroxylases. FEBS Lett. 2004;576(1-2):145-50.
12. Simicevic J, Schmid AW, Gilardoni PA, Zoller B, Raghav SK, Krier I, et al. Absolute quantification of transcription factors during cellular differentiation using multiplexed targeted proteomics. Nat Methods. 2013;10(6):570-6.
13. Xaus J, Comalada M, Valledor AF, Lloberas J, Lopez-Soriano F, Argiles JM, et al. LPS induces apoptosis in macrophages mostly through the autocrine production of TNF-alpha. Blood. 2000;95(12):3823-31.
14. Barbera-Cremades M, Gomez AI, Baroja-Mazo A, Martinez-Alarcon L, Martinez CM, de Torre-Minguela C, et al. P2X7 Receptor Induces Tumor Necrosis Factor-alpha Converting Enzyme Activation and Release to Boost TNF-alpha Production. Front Immunol. 2017;8:862.
15. Cudejko C, Wouters K, Fuentes L, Hannou SA, Paquet C, Bantubungi K, et al. p16INK4a deficiency promotes IL-4-induced polarization and inhibits proinflammatory signaling in macrophages. Blood. 2011;118(9):2556-66.
16. Marinov GK, Williams BA, McCue K, Schroth GP, Gertz J, Myers RM, et al. From single-cell to cell-pool transcriptomes: stochasticity in gene expression and RNA splicing. Genome Res. 2014;24(3):496-510.
17. Su Z, Yang R, Zhang W, Xu L, Zhong Y, Yin Y, et al. The synergistic interaction between the calcineurin B subunit and IFN-gamma enhances macrophage antitumor activity. Cell Death Dis. 2015;6:e1740.
18. Michelucci A, Cordes T, Ghelfi J, Pailot A, Reiling N, Goldmann O, et al. Immune-responsive gene 1 protein links metabolism to immunity by catalyzing itaconic acid production. Proc Natl Acad Sci U S A. 2013;110(19):7820-5.
19. Krombach F, Munzing S, Allmeling AM, Gerlach JT, Behr J, Dorger M. Cell size of alveolar macrophages: an interspecies comparison. Environ Health Perspect. 1997;105 Suppl 5:1261-3.
20. Swanson JA, Lee M, Knapp PE. Cellular dimensions affecting the nucleocytoplasmic volume ratio. J Cell Biol. 1991;115(4):941-8.
