# Supplementary material for: A mechanistic integrative computational model of macrophage polarization: Implications in human pathophysiology
Source: PLoS Comput Biol. 2019 Nov 18;15(11):e1007468. doi: 10.1371/journal.pcbi.1007468 (PMC6860420; doi:10.1371/journal.pcbi.1007468)
Supplement: S3 Table — (PDF) [file pcbi.1007468.s012.pdf]

Table S3

Description of Data Used in Calibration	*PMIDs of Sources*
**IFN-γ module (in response to IFN-γ treatments unless noted otherwise below)**	
Surface-bound IFN-γ	2953810
Phosphorylation of JAK	12667213
Phosphorylation of STAT1	16473883, 26882544, 26299368, 28280036, 10490990
Expression of IRF-1	17293456, 18802049
Expression of iNOS	18655171, 9667738
TNFα secretion	8802049
IL-12 secretion	25950470
CXCL-9 secretion	25950470
CXCL-10 mRNA expression	25918247
Expression of miR-3473b	25092892
Itaconate expression	26829557
PTEN expression upon miR-3473b overexpression	25092892
HIF1α expression upon IFN-γ treatment and hypoxia	20194441
SOCS1 mRNA expression	17093501
SOCS3 mRNA expression	17093501
HIF2α expression upon IFN-γ treatment and hypoxia	20194441
**IL-4 module (in response to IL-4 treatments unless noted otherwise below)**	
STAT6 phosphorylation	27731330, 17093501, 26894960, 26883801, 27464342, 25175012
Phosphorylated STAT6 in nucleus	23913966
IRF-4 expression	29871928, 23287596, 20580461
AKT activation	26894960, 27731330, 27507812
PPARγ expression	29203644, 24385430
Arg-1 expression	23287596, 27117406, 23913966
Arg-1 activity	22348056
IL-10 secretion	28903394, 21753147
VEGF secretion	28842601
VEGF expression (intracellular)	28903394
TNFα secretion	28903394, 21753147
HIF2α expression upon IL-4 treatment and hypoxia	20194441
SOCS1 mRNA expression	17093501
HIF1α expression upon IL-4 treatment and hypoxia	20194441
**Hypoxia module (in response to hypoxia unless noted otherwise below)**	
HIF1a stabilization	24301659, 16533170, 20644254, 19454749
HIF2a stabilization	16533170, 20644254, 19454749
iNOS expression	28211523
Arg-1 expression	28211523

TNFα secretion	22566835
IFN-γ secretion	19234213
VEGF secretion	17065555
VEGF expression (intracellular)	28903394
miR-93 expression	28356443
IFN-γ secretion upon miR-93 overexpression and hypoxia	28356443
PHD2 expression	12912907
IRF-1 expression	11313373
IRF-9 mRNA expression	28356443
IRG-1 mRNA expression	28356443
IRG-1 mRNA expression upon miR-93 overexpression	28356443
TNFα secretion upon miR-93 overexpression and hypoxia	28356443

Table S3. Summary of literature sources used in model calibration. Listed here are the descriptions and PMIDs of all the literature sources from which the model calibration datasets were extracted.
