# Supplementary material for: A mechanistic integrative computational model of macrophage polarization: Implications in human pathophysiology
Source: PLoS Comput Biol. 2019 Nov 18;15(11):e1007468. doi: 10.1371/journal.pcbi.1007468 (PMC6860420; doi:10.1371/journal.pcbi.1007468)
Supplement: S1 Protocol — (PDF) [file pcbi.1007468.s013.pdf]

Protocol S1: Additional information regarding model formulation and analysis

Model Formulation

Macrophage polarization is a complex, dynamic multi-pathway process with numerous feedbacks and cross-talks, which also makes its a highly suitable topic for systems biology modeling. Given the many relevant pathways and the large number of mechanistic details that govern macrophage polarization, it makes sense to approach this problem in a stepwise manner (e.g. first build a "beginner model", then gradually enrich the model with more pathways of high importance in disease contexts). After careful analysis of literature knowledge, we ended up choosing three pathways (IFN-γ, IL-4, hypoxia) to model in the beginning step (as presented in this paper), since IFN-γ and IL-4 are representative inducers of M1 and M2 phenotypes and many experimental studies have tried to elucidate their downstream signaling and gene regulation mechanisms (which provided a wealth of quantitative data that can be used to calibrate our "beginner model"), and also because that hypoxia is a key signature in the disease areas that we are interested in (namely peripheral arterial disease and cancer) while its direct impact on macrophage polarization and connections with other macrophage pathways have not been systematically characterized before.

Model Calibration

During model calibration, values of some model parameters are derived directly from literature data and previous models (as shown in Table S1); for the remaining parameters with no literature reference, we put in tentative values first and then hand-tuned the entire model extensively (by adjusting parameter values and observing model response) until the model simulations achieved good visual agreements with all the respective calibration datasets (from the sources listed in Table S3) simultaneously. In the meantime, the initial conditions of "unique" species in the model have to stay within the allowed ranges (0.5x-2x of concentration values estimated from literature as listed in Table S2, except for total HIF-1α and HIF-2α which we set that their resting concentrations per cell should be less than a few nanomolar according to (1)). Then we performed preliminary sensitivity analysis (for three cases, IL-4 or IFN-γ or hypoxia stimulation) and collectively identified 101 parameters that have statistically significant ($p<0.05$) PRCC values. Among the 101 parameters, 82 had no literature reference and global optimization using *patternsearch* in MATLAB was then performed for those 82 parameter values (with 0.5x-2x as the allowed ranges) with respect to all the calibration datasets (along with the initial condition

checks in every iteration) to generate the final parameter values. We rounded the final values to three significant digits for all parameters (as listed in Table S1).

For the initial condition checks, we simulated the model for 100000 minutes to obtain species endpoint values (and check if they are within appropriate ranges as described above) and use these endpoint values as new initial conditions to generate simulations and calculate squared errors with respect to the literature data in every iteration of *patternsearch* optimization. Since we selected this very long time span, in each iteration these new initial conditions obtained would represent a set of species equilibrium states (of macrophages under normoxia without externally added stimuli) that can be compared with quantitative literature data.

Model Sensitivity Analysis and Uncertainty Quantification

For model sensitivity analysis, we used Latin Hypercube Sampling method with parameter ranges of 0.5x-2x to calculate the PRCC values (with p=0.05 as the cutoff for statistical significance) based on the algorithm and code published in (2). The output of interest in PRCC calculations are "M1/M2 scores", which are the multiplication of six M1 markers ([iNOS]*[IFNγ]*[TNFα]*[IL12]*[CXCL9]*[mCXCL10]; [IFNγ] is removed when calculating PRCCs in scenarios of IFN-γ stimulation) divided by the multiplication of three M2 markers ([ARG1]*[VEGF]*[IL10]). For uncertainty quantification, each of the 50 re-sampled datasets has 229 individual datapoints that covered all the experimental conditions used in model calibration. For each datapoint, we assumed its value is within a distinct normal distribution with a mean and a standard deviation (whenever possible, the mean and standard deviations are calculated from the corresponding literature data that we gathered; for datapoints that only had one value and no repeat, we considered that single value as the mean and assumed that the standard deviation equals to 10% of that value). Then the 229 datapoint values in each re-sampled dataset were compiled in order by generating random numbers from the 229 normal distributions. The 50 re-sampled datasets were then fed into the optimization algorithm to obtain 50 sets of new parameter estimates (as described in Materials and Methods).

References

1. Tuckerman JR, Zhao Y, Hewitson KS, Tian YM, Pugh CW, Ratcliffe PJ, et al. Determination and comparison of specific activity of the HIF-prolyl hydroxylases. FEBS Lett. 2004;576(1-2):145-50.
2. Marino S, Hogue IB, Ray CJ, Kirschner DE. A methodology for performing global uncertainty and sensitivity analysis in systems biology. J Theor Biol. 2008;254(1):178-96.
